# Global distribution of α/β hydrolase family macrolide esterases in Gram-positive bacteria

**DOI:** 10.1093/ismejo/wraf261

**Published:** 2025-11-26

**Authors:** Yang Zhou, Yongqiang Yang, Yuqi Mao, Zhangqun Hou, Yiyang Xu, Kelei Zhao, Yiwen Chu, Xinrong Wang, Can Wang, Shun Li, Fei Xu, Likai Hao, Binbin Xie, Jiafu Lin, Tao Song

**Affiliations:** Antibiotics Research and Re-evaluation Key Laboratory of Sichuan Province, Sichuan Industrial Institute of Antibiotics, School of pharmacy, Chengdu University, Chengdu, Sichuan 610106, China; Center of Infectious Diseases, Center for Pathogen Research, West China Hospital, Sichuan University, Chengdu, Sichuan 610106, China; Antibiotics Research and Re-evaluation Key Laboratory of Sichuan Province, Sichuan Industrial Institute of Antibiotics, School of pharmacy, Chengdu University, Chengdu, Sichuan 610106, China; Antibiotics Research and Re-evaluation Key Laboratory of Sichuan Province, Sichuan Industrial Institute of Antibiotics, School of pharmacy, Chengdu University, Chengdu, Sichuan 610106, China; Antibiotics Research and Re-evaluation Key Laboratory of Sichuan Province, Sichuan Industrial Institute of Antibiotics, School of pharmacy, Chengdu University, Chengdu, Sichuan 610106, China; Antibiotics Research and Re-evaluation Key Laboratory of Sichuan Province, Sichuan Industrial Institute of Antibiotics, School of pharmacy, Chengdu University, Chengdu, Sichuan 610106, China; Antibiotics Research and Re-evaluation Key Laboratory of Sichuan Province, Sichuan Industrial Institute of Antibiotics, School of pharmacy, Chengdu University, Chengdu, Sichuan 610106, China; Antibiotics Research and Re-evaluation Key Laboratory of Sichuan Province, Sichuan Industrial Institute of Antibiotics, School of pharmacy, Chengdu University, Chengdu, Sichuan 610106, China; State Key Laboratory of Geohazard Prevention and Geoenvironment Protection, College of Ecology and Environment, Chengdu University of Technology, Chengdu, Sichuan 610106, China; State Key Laboratory of Microbial Technology, Shandong University, Qingdao, Shandong 266237, China; Key Laboratory of Bio-Resource and Eco-Environment of Ministry of Education, College of Life Sciences, Sichuan University, Chengdu, Sichuan 610106, PR China; State Key Laboratory of Environmental Geochemistry, Institute of Geochemistry, CAS, Guiyang, Guizhou 550081, PR China; College of Resources and Environment, University of Chinese Academy of Sciences, Beijing 100049, PR China; State Key Laboratory of Microbial Technology, Shandong University, Qingdao, Shandong 266237, China; Antibiotics Research and Re-evaluation Key Laboratory of Sichuan Province, Sichuan Industrial Institute of Antibiotics, School of pharmacy, Chengdu University, Chengdu, Sichuan 610106, China; Antibiotics Research and Re-evaluation Key Laboratory of Sichuan Province, Sichuan Industrial Institute of Antibiotics, School of pharmacy, Chengdu University, Chengdu, Sichuan 610106, China

**Keywords:** antibiotic resistance, macrolide esterases, gram-positive bacteria

## Abstract

Macrolide antibiotics are vital for controlling infections in humans, animals, and agriculture, yet their effectiveness is increasingly compromised by antimicrobial resistance. Macrolide esterases (MLEs) are key mediators of macrolide resistance but have only been detected in Gram-negative bacteria, with no evidence in Gram-positive species. Here, we mined over 500 000 Gram-positive genomes and identified 8707 candidate proteins. Six representative MLEs were functionally validated, conferring resistance to 16-membered macrolides and increasing minimum inhibitory concentrations (MICs) up to 16-fold in *Escherichia coli* and 128-fold in *Bacillus subtilis*. Moreover, two exhibited broad-spectrum activity against all clinically and veterinary relevant 16-membered macrolides. Temporal analysis revealed that Gram-positive MLEs originated at least 2.7 million years ago, contrasting with their emergence in Gram-negative bacteria after the introduction of antibiotics. Genomic surveys further demonstrated the global distribution of MLE-carrying Gram-positive bacteria across 97 countries and diverse ecosystems, including clinical, food, agricultural, and natural environments. These findings highlight Gram-positive MLEs as an underrecognized risk and underscore the need for a One Health–oriented strategy to monitor, assess, and mitigate the spread of macrolide resistance across interconnected ecosystems.

## Introduction

Macrolide antibiotics are the second most widely used class of antibiotics worldwide [1]. They exert their antimicrobial effects by binding to the bacterial 50S ribosomal subunit, thereby inhibiting protein synthesis [2–4]. These antibiotics are particularly effective against Gram-positive bacteria due to their high molecular weight and lipophilicity, which enable better penetration of the Gram-positive bacterial cell wall compared to Gram-negative bacteria [5]. However, the effectiveness of macrolides is increasingly compromised by resistance mechanisms, including antibiotic inactivation [6–8], target alteration [9, 10], efflux [11, 12], and target protection [13, 14]. For example, erythromycin rRNA methylases (erm) are found in *Streptococcus pyogenes* [15], macrolide phosphotransferases in *Bacillus* species [16], and macrolide glycosyltransferases in *Streptomyces antibioticus* [17].

Macrolide esterase (MLE) represents an important resistance mechanism against macrolide antibiotics, functioning by hydrolyzing the macrolide ring structure and thereby disabling their antimicrobial activity. The known MLEs primarily belong to the Erythromycin esterase (Ere) family and the α/β hydrolase superfamily. The Ere family—identified in the 1980s and currently comprising five members—is primarily responsible for degrading 14- and 15-membered macrolides [18–21]. These enzymes are commonly found in clinical strains of Gram-negative bacteria, such as *Escherichia coli* [18], multidrug-resistant *Klebsiella pneumoniae* [20], and *Riemerella anatipestifer* [21]. In contrast, MLEs from the α/β hydrolase superfamily primarily target 16-membered macrolides and include only two recently discovered enzymes, EstT and EstX. Both EstT and EstX are derived from Gram-negative bacteria. EstT was discovered in 2023 in a multidrug-resistant *Sphingobacterium faecium* WB1 strain isolated from farm water bowls [8], while EstX, identified in 2024, was found in *E. coli*, *K. pneumoniae*, and *Salmonella* isolates with a global distribution [22]. Although MLE activity has been detected in Gram-positive bacteria, the genetic basis for this activity remains unidentified [23]. This knowledge gap concerning MLEs in Gram-positive pathogens poses a significant public health concern.

The distribution of MLEs among Gram-positive and Gram-negative bacteria differs from that of other known macrolide resistance mechanisms. Most macrolide resistance mechanisms are found in Gram-positive bacteria, with a few reports in Gram-negative bacteria [15, 24, 25]. Researchers have hypothesized that naturally resistant Gram-negative bacteria may acquire macrolide resistance genes from Gram-positive bacteria, thereby further enhancing their inherent resistance [26]. For example, the presence of macrolide resistance genes in Gram-negative bacteria may elevate the minimum inhibitory concentration (MIC) to mg/ml levels to adapt exceptionally high concentrations of macrolides in human intestinal environment [26]. However, MLEs have thus far been identified exclusively in Gram-negative pathogens, but not in Gram-positive bacteria, which is unusual.

In recent decades, efforts to expand the known members of MLEs have encountered challenges. MLEs from the Ere family show limited homologues in available databases and attempts to functionally characterize distant homologues have been unsuccessful [27]. Recently identified MLEs belong to the α/β hydrolase superfamily, which contains 23 more fold members than the Ere family (according to InterPro data). It remains to be explored whether this family contains additional MLEs beyond the two already identified from Gram-negative bacteria, particularly those from Gram-positive bacteria. To address this, we employed genomic mining and phylogenetic analyses to select and biochemically characterize representative MLEs from Gram-positive bacteria. Additionally, we investigated the global distribution and emergence timeline of bacteria carrying MLE homologues to elucidate their possible transmission pathways, thereby providing insights into the dissemination mechanisms of MLEs. When interpreted within an integrated “One Health” framework (which considers the interconnected health of humans, animals, and the environment), these analyses reveal how microbial interactions across diverse ecological and host-associated niches collectively shape the global landscape of macrolide resistance [28, 29].

## Materials and methods

### Database construction and gene mining

To mine MLEs from Gram-positive bacteria, we selected members primarily from the *Firmicutes* and *Actinobacteria* phyla, which are predominantly Gram-positive. Bacterial genome data from *Firmicutes* (*n =* 448 178) and *Actinobacteria* (*n =* 53 829) were retrieved from the NCBI GenBank database (https://www.ncbi.nlm.nih.gov/genbank/) on June 15, 2023. The database comprises a total of 502 007 bacterial genomes, including 411 370 contigs, 68 317 scaffolds, 19 621 complete genomes, and 2699 chromosomes, and covers genomes from both isolated strains (478192) and metagenomic assemblies (23815) (Supplementary material 1). The corresponding protein sequences were extracted and compiled into a single FASTA file for downstream analysis. Low-quality genomes were filtered out using BUSCO to ensure a sequence completeness of over 90% and a contamination rate below 10% before analysis [30]. Experimentally characterized MLE sequences were collected to serve as query sequences. Potential MLE homologs were then identified using DIAMOND with the following parameters: an e-value threshold of 1e-10, a minimum protein identity of 40%, and a sequence coverage of at least 80% [31]. A sequence identity threshold of 40% is commonly employed to exclude proteins that are likely to differ in function [32, 33]. Consistent with this, our preliminary experiments also showed that when sequence identity fell below 40%, no detectable MLE activity was observed (Supplementary material 2). Bacterial genomes carrying MLEs were taxonomically classified with GTDB-Tk v2.2.0 to confirm their species assignments, referencing the Genome Taxonomy Database (GTDB) [34].

### Phylogenetic tree analysis and representative sequences selection

Potential MLE homologs together with experimentally characterized MLE sequences and outgroup proteins (Aclacinomycin methylesterase RdmC, Q54528.1, and Rhodomycin D methylesterase DnrP, Q54809.1), were used to construct a phylogenetic tree. We used CD-HIT [35] to remove redundant sequences with over 99% sequence similarity and over 99% sequence coverage. IQ-TREE [36] was employed for tree inference, with ModelFinder selecting Q.pfam+G4 as the best-fit model. To assess the robustness of the tree, 1000 bootstrap replicates were performed. Finally, the phylogenetic tree was visualized using the Chiplot program (https://www.chiplot.online/). To further reduce the number of sequences to be selected and choose representative sequences, we clustered the sequences using a relaxed criteria (80% sequence similarity and 80% sequence coverage), then examined the topological structure and placement of the selected representative sequences on the phylogenetic tree, removing sequences that were located within the same subclade. An 80% amino-acid sequence identity threshold is used as an empirical guideline for defining gene families, based on previous studies [37, 38].

### Construction of *E.coli* and *B. subtilis* carrying macrolide esterases

The genes encoding six MLEs (*mle*_BCA-1_, *mle*_BCB-1_, *mle*_BRB-1_, *mle*_PBB-1_, *mle*_CBF-1_, and *mle*_PBA-1_; protein accession numbers: PQ676745, PQ676746, PQ676747, PQ676748, PQ676749, PQ623322) were codon-optimized, synthesized and transformed into *E. coli* and *B. subtilis*, respectively. For the *E. coli* expression system, the target gene was cloned between the *Hind*III and *Not*I restriction sites of the pET-28a(+) vector. This construct utilizes the T7 promoter to drive the expression of the gene of interest and incorporates a C-terminal 6 × His tag. The recombinant plasmid was then transformed into the *E. coli* BL21(DE3) host strain for protein expression. For the *B. subtilis* expression system, the pHT43 vector was employed. The target gene was inserted into the *Bam*HI site of the vector using a seamless cloning strategy. This construct leverages the Pgrac promoter to drive the transcription of the gene of interest. Subsequently, the recombinant plasmid was introduced into the *B. subtilis* WB800N strain via natural transformation [39] (Supplementary material 3 and Supplementary materials 18–21).

### Protein expression in *E.coli*

The transformed bacteria were cultured in LB broth containing 100 μg/ml kanamycin. When the optical density at 600 nm (OD600) reached 0.6–0.8, 1 mM IPTG was added to induce protein expression, and the cultures were incubated overnight at 25°C with shaking at 180 rpm. After harvesting by centrifugation, the cell pellet was resuspended in Buffer A (20 mM Tris–HCl, 500 mM NaCl, pH 7.0) and disrupted by ultrasonication. The cell lysate was centrifuged for 20 min, and the supernatant was applied to a HisTrap column for immobilized metal affinity chromatography (IMAC) to purify the target protein. The purified protein was dialyzed three times against 20 mM Tris–HCl (pH 7.0) to remove any remaining impurities and stored at 4°C for further experiments. Protein purity was confirmed by SDS-PAGE, and protein concentration was determined by measuring absorbance at 280 nm, using the calculated molar extinction coefficient.

### Determination of minimum inhibitory concentration

The MIC was determined by the broth microdilution method. The macrolide antibiotics tested included erythromycin, roxithromycin, clarithromycin (14-membered ring), azithromycin, tulathromycin, gamithromycin (15-membered ring), tylosin, tilmicosin, tildipirosin, kitasamycin, josamycin, spiramycin, midecamycin, and acetylspiramycin (16-membered ring). The MIC of antibiotics against *E. coli* and *B. subtilis* harboring MLE plasmids was determined using the broth microdilution method. *E. coli* was cultured overnight in Mueller-Hinton (MH) broth supplemented with 100 μg/ml kanamycin and 1 mM IPTG, whereas *B. subtilis* was cultured in the same medium containing 5 μg/ml chloramphenicol and 1 mM IPTG. The cultures were then adjusted to an optical density (OD) of 1.0 at 600 nm and diluted 1:1000 into fresh MH broth containing 1 mM IPTG, resulting in ~10^5^ colony-forming units (CFUs) per well. Serial two-fold dilutions of each antibiotic were prepared in the wells of a 96-well microtiter plate, and 100 μL of the diluted bacterial culture was added to each well. The plates were incubated at 37°C with shaking at 180 rpm for 20 h. Bacterial growth was assessed by measuring the OD at 600 nm, and the MIC was defined as the lowest antibiotic concentration that completely inhibited visible growth.

### Inhibition zone analysis of enzyme-degraded macrolide antibiotics

The antibacterial activity of the antibiotic before and after degradation was tested using the macrolide-sensitive strain *Staphylococcus aureus* ATCC 25923. Briefly, 15 μL of an overnight culture of *S. aureus* (OD600 ≈ 1.0) was mixed with 15 ml of LB containing 1.5% agar, preheated to ~40°C. After thoroughly mixing, the agar was poured into 90-mm Petri dishes. Once the agar solidified, 4.5 mm diameter holes were punched into the agar using a sterile puncher. Subsequently, 30 μL of the enzyme-treated macrolide antibiotic solution (and the corresponding control) was added to the wells. The plates were incubated at 37°C for 24 h, and the diameters of the inhibition zones were measured to evaluate antibacterial activity.

### Mass spectrometry analysis of enzyme-degraded macrolide antibiotics

900 μL of a 50 μg/ml macrolide antibiotic solution was individually mixed with each of the six MLEs (0.30 mg/ml). The reaction mixture was incubated overnight at 37°C, with inactivated enzyme used as the control. The reaction was terminated by heat inactivation of the enzyme at 100°C for 10 min. The antibiotic degradation products were analyzed by electrospray ionisation mass spectrometry (ESI-MS). The analysis was performed using a Waters Xevo G2-XS Q-TOF mass spectrometer equipped with an ESI source operated in positive mode. Mass spectrometry was performed in the scanning range of 100–1200 *m/z*. The ion source parameters were as follows: curtain gas pressure at 40 psi, ion source gas 1 at 30 psi, ion source gas 2 at 30 psi, ESI+ voltage at 5500 V, and ESI− voltage at 4500 V. The drying gas temperature and flow rate were maintained at 450°C and 5 L/min, respectively.

### 3D structural and substrate binding site prediction of macrolide esterase

The 3D structure of MLE was predicted using AlphaFold3 [40], employing the following parameters: number_recycles = 50, max_msa = 512–1024, number_relax = 5, rank_plddt, and number_models = 5. The quality of the predicted MLE structures was evaluated using AlphaFold's PLDDT score, which measures the confidence of each residue's predicted structure based on its local distance difference test score. Additionally, PROCHECK was used to generate Ramachandran plots, assessing the stereochemical quality and overall validity of the models [41]. The structural models were then visualized using Chimera [42]. The binding pocket of MLE was predicted using the PrankWeb server [43] (https://prankweb.cz/). The evolutionary conservation of residues in MLE was predicted using the ConSurf server (https://consurf.tau.ac.il/) [44]. The conservation scores were mapped onto the 3D structure and visualized using Chimera to identify functionally important residues. Domain analysis of the six MLEs was performed using the InterPro web server (http://www.ebi.ac.uk/interpro/), with results visualized through the Chiplot program.

### Site-directed mutagenesis of macrolide esterase

To further explore the catalytic mechanism of MLE, site-directed mutagenesis was employed. The mutants including S102A, D230A, H258A were synthesized and inserted into the *Hind*III and *Not*I restriction sites of the pET-28a(+) expression vector. The resulting recombinant vector was transformed into *E. coli* BL21(DE3) cells, and successful gene integration was verified through colony PCR and sequencing. After confirming the sequence, protein expression was induced, following the same protocols for expression, purification, enzyme activity analysis, and MIC determination as outlined in earlier sections.

### Mobile genetic elements prediction

To further investigate the potential mobility of MLEs, genomic regions surrounding each MLE were analyzed for the presence of mobile genetic elements (MGEs), including insertion sequences (ISs), integrons, and plasmids. For IS and integron prediction, a total of 11 genes were examined for each locus, comprising the MLE gene itself and its five flanking genes upstream and downstream. ISfinder (updated in June 2024) was used to identify ISs [45], and IntegronFinder (version 2.0) was employed to detect integrons [46], both using the latest reference databases. MLEs located on short contigs with fewer than five flanking genes on either side were excluded from IS and integron prediction. In contrast, plasmid prediction was performed using the entire contig containing the MLE, based on replication genes (e.g. *rep*), transfer genes (e.g. *mob*, *tra*), and characteristic plasmid-associated features annotated by the NCBI Prokaryotic Genome Annotation Pipeline (PGAP, version 1.2) [47]. Only contigs longer than 5 kb were included in plasmid prediction to ensure reliable identification of replication and transfer-associated genes.

**Figure 1:**
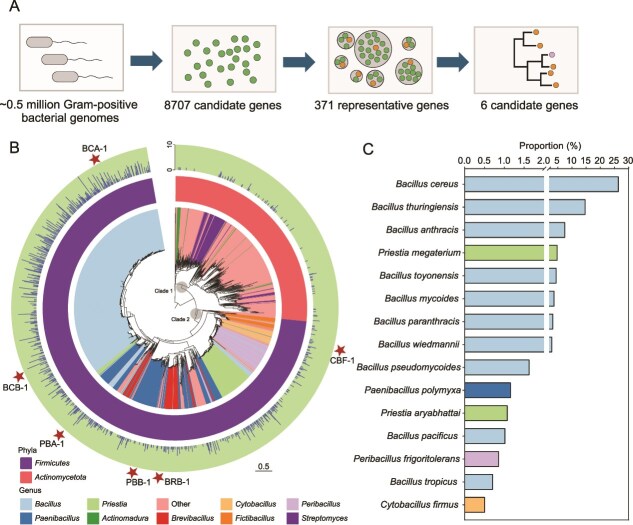
Genome mining of macrolide esterases (MLEs) and taxonomic distribution of MLE-harboring Gram-positive bacteria. (A) Workflow illustrating the mining process used to identify MLEs within Gram-positive bacterial genomes. (B) Maximum-likelihood phylogenetic tree of MLEs identified from Gram-positive bacteria. Each node represents a nonredundant sequence that stands for a cluster of proteins sharing ≥99% sequence identity and ≥99% alignment coverage. Based on the tree topology, two major clades were defined (Clade 1 and Clade 2), which are highlighted by gray circles. The inner ring denotes the genus-level distribution of bacteria carrying the corresponding MLEs; the middle ring indicates their phylum-level assignments; the outer blue bars represent outer blue bars indicate the log₂-transformed cluster size for each node. The scale bar corresponds to 0.5 substitutions per site. (C) Relative abundance of Gram-positive bacterial species harboring MLEs, with the top 15 most abundant species shown.

### Information collection of macrolide esterase-carrying bacteria

To minimize the false-positive rate, we selected six validated MLEs as query sequences. DIAMOND, a high-throughput protein alignment tool, was used to identify MLE homologues. To avoid false positives, only genes encoding proteins with >80% sequence similarity to the characterized MLEs were considered. Then, the information corresponding to the bacterial geographic distribution, discovery time, and ecological environment was obtained from the NCBI database. We acknowledge that NCBI Biosample metadata may reflect current sequencing preferences and therefore contain certain sampling biases. Nevertheless, it remains a comprehensive, systematically curated, and widely used repository of genome-associated metadata.

## Results and discussion

### Screening of macrolide esterases in gram-positive bacteria

To find potential MLEs in Gram-positive bacteria, we collected 502 007 high-quality Gram-positive bacterial genomes from NCBI genome database, including those from *Firmicutes* (*n =* 448 178) and *Actinobacteria* (*n =* 53 829) (Fig. 1A). Enzyme sequences from Ere family and α/β hydrolase superfamily were used for the search, with a cut-off value of 40% protein sequence identity and >80% coverage applied to minimize potential false positives. We obtained 8707 hits from the α/β hydrolase family, but no hits from Ere family (Fig. 1A).

We conducted a phylogenetic tree analysis to infer the MLEs' evolutionary process. Maximum-likelihood phylogenetic analysis of 8707 sequences revealed two distinct clades: Clade 1 from *Actinobacteria* and Clade 2 from *Actinobacteria* and *Firmicutes* (Fig. 1B). Clade 1 and clade 2 were designated based on phylogenetic topology and the species distribution within the clade. Clade 1 predominantly comprises sequences from the genera *Streptomyces*, *Actinomadura*, *Amycolatopsis*, and *Nocardia*. In contrast, Clade 2 is primarily composed of sequences from *Firmicutes*, including *Bacillus*, *Priestia*, *Paenibacillus*, *Clostridium*, and *Brevibacillus*. The short branch lengths of sequences from *Bacillus* species suggest they share high sequence similarities. Many MLEs were present in potential pathogens, including *B. cereus* (26.4%, *n =* 2079), *B. thuringiensis* (14.7%, *n =* 1158), *B. anthracis* (7.5%, *n =* 591), *B. paranthracis* (3.3%, *n =* 259), *C. perfringens* (0.5%, *n =* 40), and *S. arlettae* (0.3%, *n =* 20) (Fig. 1B and Supplementary material 4). Although *Bacillus* species represent only ~2.0% of the *Firmicutes* genome database, they contain more than 52% of all identified MLEs (Supplementary material 1). This pattern indicates that *Bacillus* species form a substantial reservoir of these resistance elements and may, in addition, serve as their natural hosts.

To select representative sequences for functional characterization, 8707 candidate genes were re-clustered into 371 groups using a relaxed criterion (80% sequence identity and coverage) (Fig. 1A and Supplementary data 1). Six representative sequences were chosen from the six largest clusters, which collectively encompassed 7291 sequences (83.7% of the total). These sequences were located in different subclades of Clade 2, the clade containing the highest number of sequences on the phylogenetic tree (Fig. 1B). They were BCA-1 from *B. cereus*, BCB-1 from *B. cereus*, BRB-1 from *Brevibacillus brevis*, PBB-1 from *Paenibacillus* sp., and PBA-1 from *Paenibacillus* sp., CBF-1 from *Cytobacillus firmus*. These six MLEs exhibited limited sequence identity with reference esterases EstT/EstX (42.1%–51.3%) whereas they display substantial divergence among themselves (42.1%–78.8% pairwise identity), with distinct phylogenetic placements across clades and therefore they were selected for further functional characterization (Supplementary material 5).

### Gram-positive macrolide esterase conferred increased resistance to macrolides

**Figure 2:**
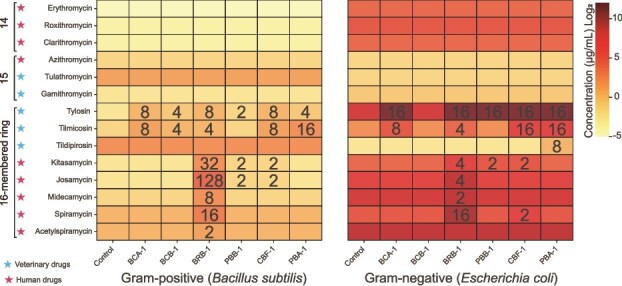
Macrolide susceptibility profiles of Gram-positive and Gram-negative bacteria carrying macrolide esterases (MLEs). Heatmaps display the minimum inhibitory concentrations (MICs) of 14 macrolide antibiotics—including 14-, 15-, and 16-membered macrolides—against *E. coli* and *B. subtilis* strains expressing six representative MLEs. MICs were determined using a broth microdilution assay. Antibiotics approved for human use and veterinary applications are marked with red and blue five-pointed stars, respectively. Numerals within the heatmaps denote the fold change in MIC relative to the corresponding empty-vector control strains.

To investigate whether MLEs could alter bacterial macrolide resistance profiles, six representative MLEs were synthesized and expressed in *Bacillus subtilis* (Gram-positive) and *E. coli* (Gram-negative), respectively. The expression of six MLEs in both *E. coli* and *B. subtilis* led to an increase in MICs against 16-membered macrolide antibiotics, but not for 14- or 15-membered macrolides (Fig. 2). In *B. subtilis*, all six MLEs elevated tylosin MICs by 2- to 8-fold, with five MLEs concurrently increasing tilmicosin MICs by 4- to 16-fold. Similarly, in *E. coli*, five MLEs induced 16-fold increases in tylosin MICs, and four MLEs raised tilmicosin MICs by 4- to 16-fold. The MLE-mediated resistance profiles differed between Gram-negative and Gram-positive hosts. For instance, *B. subtilis* harboring PBA-1 exhibited no change in MIC against the tested macrolides, whereas its expression in *E. coli* resulted in an 8-fold MIC increase. Similar host-dependent disparities were observed for BCB-1, PBB-1, and CBF-1, where resistance potentiation occurred preferentially in Gram-negative *E. coli* (4- to 16-fold increases in MIC) but remained negligible in Gram-positive *B. subtilis* (Fig. 2). The observed resistance phenotypes may be influenced by factors such as gene copy number and promoter strength in the heterologous hosts, which could differ from the expression levels in natural bacterial backgrounds.

*E. coli* and *B. subtilis* carrying empty vectors (without MLEs) exhibited different MIC levels against various macrolides. *E. coli* exhibited high resistance to 14-membered macrolides with MIC values ranging from 16 to 32 μg/ml, which are 512-fold higher than those observed for *B. subtilis* (MIC: 0.03125 to 0.0625 μg/ml). A similar pattern was observed for most 16-membered macrolides, with *E. coli* showing 128- to 1024-fold MIC values compared to that of *B. subtilis*. This is consistent with the intrinsic resistance mechanisms of Gram-negative bacteria, primarily mediated by limited outer membrane permeability. We found that MIC values between *E. coli* (0.25 to 0.5 μg/ml) and *B. subtilis* (0.25 to 2 μg/ml) become comparable against 15-membered macrolides, potentially due to structural features that enhance penetration through the outer membrane in Gram-negative bacteria (Fig. 2).

BRB-1 conferred resistance to a broad-spectrum of 16-membered macrolides. In *B. subtilis*, it enhanced the MICs (2- to 128-fold) for seven of the eight tested antibiotics (excluding tildipirosin), whereas in *E. coli*, it increased MICs (2- to 16-fold) for six antibiotics (excluding tildipirosin and acetylspiramycin) (Fig. 2). This broad antibiotic resistance profile contrasts with the narrow substrate specificity of previously characterized esterases, EstX and EstT, which confer resistance solely to veterinary 16-membered macrolides (tylosin, tilmicosin, and tildipirosin). Whereas EstX also hydrolyzes the human therapeutic agent leucomycin A5 [22], BRB-1's ability to potentiate resistance across both human- and veterinary-use macrolides underscores its broader potential impact on macrolide efficacy.

### Broad substrate spectrum of macrolide esterases

**Figure 3:**
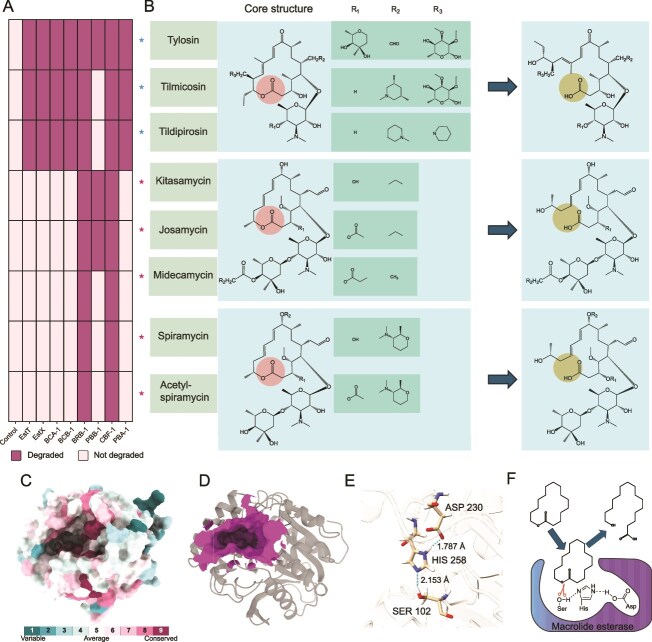
Catalytic mechanism and substrate degradation spectrum of macrolide esterases (MLEs). (A) Heatmap illustrating the substrate degradation profiles of six representative MLEs toward various macrolide antibiotics. Substrate degradation was assessed using agar diffusion (inhibition zone) assays and mass spectrometry analysis. (B) Schematic representation of macrolide hydrolysis by MLEs. Sites altered before and after degradation are marked with red and yellow circles, respectively. (C) ConSurf analysis of esterase BRB-1, shown as a surface-rendered 3D structure color-coded according to residue conservation scores. (D) Cartoon representation of the BRB-1 3D structure, with PrankWeb-predicted ligand-binding pockets displayed as purple surfaces, visualized using ChimeraX. (E) Predicted BRB-1 structure highlighting the catalytic triad (Ser102, Asp230, and His258). Hydrogen bonds are shown as dashed lines, with accompanying numerical labels indicating bond lengths (Å). (F) Proposed catalytic mechanism of MLEs, in which the catalytic triad hydrolyzes the ester bond of 16-membered macrolide antibiotics, leading to cleavage of the macrolactone ring.

Inhibition zone assays were conducted to assess the antimicrobial activity of antibiotics before and after MLE degradation (Fig. 3A). Although all enzymes demonstrated catalytic activity against 16-membered macrolides, their substrate specificities varied. BCA-1, BCB-1, and PBA-1 selectively hydrolyzed veterinary antibiotics (tylosin, tilmicosin, and tildipirosin), similar to the activity of EstT. In contrast, PBB-1 targeted a mixed profile, hydrolyzing tylosin, kitasamycin, and josamycin. BRB-1 and CBF-1 exhibited exceptional catalytic versatility, efficiently degrading eight substrates, including both veterinary antibiotics (tylosin, tilmicosin, tildipirosin) and human therapeutic agents (kitasamycin, spiramycin, josamycin, midecamycin, and acetyl-spiramycin) (Fig. 3A and B).

Mass spectrometry analysis of the degradation products showed a characteristic +18 Da mass shift after hydrolysis, consistent with the opening of the lactone ring via the addition of a water molecule (Fig. 3F and Supplementary materials 6–11). These results were consistent with the inhibition zone assays. Specifically, the molecular masses of antibiotics degraded by BRB-1 and CBF-1 both increased by 18 Da, whereas the other MLEs were able to hydrolyze only three types of 16-membered macrolide antibiotics (Supplementary materials 6–11). Domain analysis revealed that all six identified MLEs belong to the α/β hydrolase superfamily. No transmembrane domains or signal peptides were detected, suggesting that these enzymes are soluble cytosolic enzymes involved in intracellular metabolic processes (Supplementary material 12).

### MLE in gram-positive bacteria uses catalytic triad for macrolide degradation

Although Gram-negative bacterial MLEs are known to utilize a catalytic triad (Ser, Asp, His) for their hydrolytic activity, the catalytic mechanism in Gram-positive bacterial MLEs remains unexplored [8, 22, 48]. Multiple sequence alignment revealed that six MLEs share the same catalytic triad residues as the functionally characterized esterases EstX and EstT (Supplementary material 13). Predicted structures of these six MLEs demonstrated that the conserved residues are predominantly located within the substrate-binding pocket, with the catalytic triads being spatially proximal in all cases (Fig 3C–E and Supplementary material 14). In the BRB-1 structure, the catalytic triad (Ser102, Asp230, His258) is stabilized by specific hydrogen-bond interactions: Ser102 forms a 2.15 Å bond with His258, whereas Asp230 shows a shorter interaction (1.787 Å) with His258 (Fig. 3E).

To validate the functional significance of the catalytic triad in Gram-positive MLEs, we constructed three catalytic triad mutants of BRB-1 (S102A, D230A, and H258A). *E. coli* expressing these mutant variants showed MIC values comparable to those observed in empty vector controls (Supplementary material 15). Biochemical analyses subsequently demonstrated complete ablation of enzymatic activity in the purified mutated proteins, as evidenced by their inability to hydrolyze both the model esterase substrate p-nitrophenyl butyrate (pNPB) and natural 16-membered macrolide substrates. Therefore, the catalytic triad residues (Ser102, Asp230, and His258) are indispensable for the hydrolytic function of Gram-positive bacterial MLEs against 16-membered macrolide antibiotics.

### Global distribution of gram-positive bacteria carrying macrolide esterase genes

**Figure 4:**
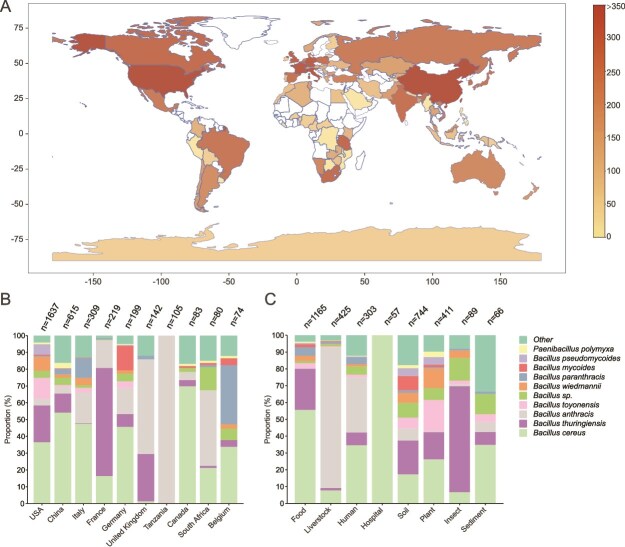
Global and ecological distribution of Gram-positive bacteria harboring macrolide esterases (MLEs). (A) World map depicting the global distribution of Gram-positive bacteria harboring MLEs. Countries without MLE-harboring bacteria are shown in white, whereas color intensity represents the abundance of MLE-harboring bacteria. The x- and y-axes correspond to longitude and latitude, respectively. Only bacterial samples carrying MLEs with ≥80% sequence similarity to characterized MLEs were included. (B) Stacked bar plots showing the proportional distribution of bacterial species harboring MLEs across the top 10 countries ranked by prevalence. (C) Stacked bar chart illustrating the ecological distribution of MLE-harboring bacteria across human-associated and non-human-associated environments.

We investigated the global distribution of Gram-positive bacteria carrying MLEs. Our analysis based on publicly available NCBI Biosample metadata revealed the widespread presence of MLE-harboring Gram-positive bacteria across 97 countries spanning all seven continents (Fig. 4A and Supplementary data 2). The United States (31.9%, *n =* 1637) and China (12.0%, *n =* 615) exhibited the highest prevalence of MLE-carrying bacteria, which aligns with their substantial antibiotic consumption—5747.7 tons and 32776.3 tons in 2020, respectively, both ranking among the top three globally. Other countries with notable prevalence include Italy (6.0%, *n =* 309), France (4.3%, *n =* 219), Germany (3.9%, *n =* 199), the United Kingdom (2.8%, *n =* 142), Tanzania (2.1%, *n =* 105), South Africa (1.6%, *n =* 80), Canada (1.6%, *n =* 83), and Belgium (1.4%, *n =* 74) (Fig. 4B).

Tanzania and South Africa reported high proportions of *B. anthracis* carrying MLEs, with prevalence rates of nearly 100% and 45%, respectively (Fig. 4B). This trend likely reflects the endemic status of anthrax in certain African regions, in contrast to its relatively low incidence in developed countries [49]. These findings provide context for *B. anthracis* distribution but may not directly reflect MLE gene frequency. Among the top 10 countries with the highest prevalence, the proportion of MLE-carrying bacterial strains exceeds 40%, highlighting the widespread distribution of MLE-carrying potential pathogens across different countries.

MLE-carrying bacteria were found not only in the six continents inhabited by humans but also in Antarctica's permafrost and in marine samples from the Atlantic and Indian Oceans (Supplementary material 16). This distribution suggests that MLE-harboring bacteria may occupy a wide range of ecological niches, highlighting their potential for global dispersion and environmental resilience. The global spread of MLE-carrying bacteria underscores the need for a coordinated, ecosystem-level strategy to address resistance reservoirs across interconnected biological and ecological systems.

### Ecological habitats of gram-positive bacteria carrying macrolide esterase genes

We examined the ecological habitats of MLE-carrying Gram-positive bacteria in non-human-associated and human-associated environments. MLE-carrying Gram-positive bacteria are widely distributed across diverse natural habitats, including soil (14.5%, *n =* 744), plants (8.0%, *n =* 411), insects (1.7%, *n =* 89), and sediments (1.3%, *n =* 66) (Fig. 4C). They have also been isolated from extreme environments such as deserts (e.g. GCA_003269305.1), hot springs (e.g. GCA_001619755.1), deep-sea sediments (e.g. GCA_016605985.1), glaciers (e.g. GCA_025809295.1), and even the International Space Station (e.g. GCA_013345865.1). This widespread distribution reflects the remarkable stress resistance of *Bacillus* species, which can form spores under adverse conditions, allowing them to survive and persist in diverse and extreme habitats [50].

MLE-carrying bacteria are also prevalent in human-associated environments, including food (22.7%, *n =* 1165), livestock (8.3%, *n =* 425), humans (5.9%, *n =* 303), and hospitals (1.1%, *n =* 57) (Fig. 4C). In these human-associated environments, the proportion of conditional pathogens exceeds 50%, which is higher than in non-human-associated environments. Among them, *B. cereus* is more prevalent in food sources, whereas *B. anthracis* is most common in animal sources. This likely reflects the role of *B. cereus* in foodborne infection outbreaks and *B. anthracis* as the main agent of anthrax.

We observed that some *Bacillus* strains used in vaccine and pesticide production carry MLE genes. For example, *B. anthracis* (e.g. GCA_000310045.1) is utilized in vaccine development, and *B. thuringiensis* (e.g. GCA_020775255.1, GCA_020775305.1) serves as a biological pesticide producer. Currently, it remains unclear whether the industrial use of MLE-carrying *Bacillus* strains could facilitate the dissemination of these genes across ecosystems. Viewed through a One Health lens that emphasizes cross-sectoral bio-surveillance, systematic monitoring of these industrially applied *Bacillus* strains and assessing their potential for horizontal gene transfer (HGT) are essential for a comprehensive biosafety evaluation.

### Different macrolide esterase dissemination patterns in gram-positive and gram-negative bacteria

**Figure 5:**
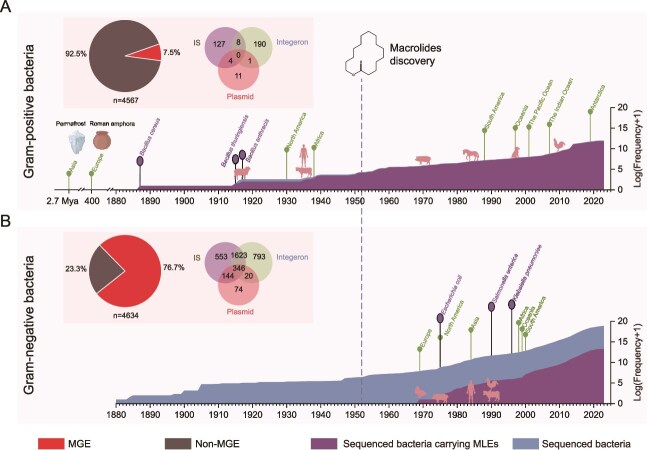
Timeline of the emergence of bacteria harboring macrolide esterase (MLEs). (A) Temporal distribution of macrolide esterase (MLE) in Gram-positive bacteria and their association with mobile genetic elements (MGEs). (B) Temporal distribution of MLE genes in Gram-negative bacteria and their association with MGEs. Only bacterial species with more than 10 MLE-positive occurrences were included. The pie chart shows the proportion of MLE-harboring bacterial species containing MGEs versus those without MGEs. The Venn diagram illustrates the distribution of MGEs among MLE-harboring bacterial species: purple regions indicate insertion sequences (IS), green regions denote integrons, and red regions represent plasmids. Overlapping areas correspond to MGEs shared between two or three categories. Timeline annotations are color-coded as follows: green lines, initial detection of MLE-harboring strains across continents and marine ecosystems; purple lines, emergence of bacterial species carrying MLEs; dashed lines, milestones of macrolide antibiotics (discovery and clinical deployment); pink zoomorphic icons, hosts of the earliest-known MLE-harboring bacterial strains. Permafrost and Roman amphorae are indicated by their respective icons.

We sought to investigate whether the spread of MLEs is linked to human antibiotic use by analyzing the time at which MLE-carrying bacteria emerged (Fig. 5A and B). These resistance genes were found in permafrost samples dating back 2.7 million years, as well as in Roman pottery from AD 400 [51, 52]. Additionally, between 1887 and 1953, a period prior to the widespread use of macrolides. MLE-carrying bacteria were also identified in hosts such as humans (e.g. GCA_022014755.1), sheep (e.g. GCA_017813925.1), and cattle (e.g. GCA_029695545.1) (Fig. 5A and Supplementary data 2). These timelines, which predate the discovery and use of macrolide antibiotics, support the view that resistance genes are naturally occurring rather than being selected by antibiotic use. Therefore, in Gram-positive bacteria, MLEs are inherited from ancient bacteria, with an estimated divergence occurring at least 2.7 million years ago, rather than being related to the extensive use of antibiotics in modern times. This is consistent with the previous report that antibiotic resistance genes existed in natural environments before human use [53, 54].

MLE-carrying strains in Gram-negative bacteria emerged only after the widespread use of macrolide antibiotics in the 1950s. The appearance was in ducks in the UK in 1969 (e.g. GCF_028680055.1), followed by pigs in 1975 (e.g. GCA_012846185.1), cattle in 1983 (e.g. GCA_004794935.1), humans in 1984 (e.g. GCA_028898885.1), and later in chickens in 1990 (e.g. GCA_013047325.1) (Fig. 5B and Supplementary data 3). Given that 5032 bacterial strains collected prior to 1969 have already been sequenced, it seems unlikely that the absence of MLE-carrying bacteria is due to insufficient genomic data. Therefore, we observed two distinct patterns: in Gram-positive bacteria, MLEs originated from ancient bacteria, whereas in Gram-negative bacteria, MLEs were acquired from other sources as a result of selective pressure induced by antibiotic use.

To assess the prevalence of MLEs within different bacterial populations, we calculated the ratio of MLE-carrying bacteria to the total number of sequenced bacteria within the same species (Supplementary material 17). In Gram-positive bacteria, prior to 1950, the proportion varied widely due to the limited sample size, fluctuating between 50% and 100%. However, with the increase in sequencing efforts over time, this proportion stabilized around 91%. The proportions of MLE-carrying genomes are particularly high in *Bacillus* species, including *B. cereus* (95.9%, *n =* 1576), *B. anthracis* (95.1%, *n =* 443), and *B. thuringiensis* (97.8%, *n =* 717). The consistently high proportion of MLE-containing genomes across multiple *Bacillus* species, together with our earlier finding that the majority of MLE-positive genomes originate from this genus, strongly suggests that *Bacillus* may represent the natural host of these elements. Given that only 7.5% of MLEs are associated with MGEs, this suggests that the spread of MLEs in Gram-positive bacteria is predominantly through clonal propagation (Fig. 5A).

The proportion of MLE-carrying Gram-negative bacteria stabilized at ~2%. The proportion of MLE-carrying genomes in Gram-negative bacteria varies across species (Supplementary material 17). For example, in *S. enterica* (1.0%, *n =* 2474), *E. coli* (3.5%, *n =* 5439), and *K. pneumoniae* (3.0%, *n =* 1095), the proportion of MLE-carrying genomes is relatively low compared to Gram-positive bacteria. MLE-carrying bacteria were more prevalent in livestock pathogens, such as the opportunistic pathogens *Histophilus somni* in cattle/sheep (29.8%, *n =* 17), *R. anatipestifer* in ducks (59.4%, *n =* 205), and *Glaesserella parasuis* in pigs (20.6%, *n =* 114). This prevalence is likely due to the selective pressure exerted by the routine use of 16-membered macrolides in veterinary medicine, which may promote the selection of MLEs that can inactivate these antibiotics. Furthermore, HGT may play an important role in the spread of MLEs in Gram-negative bacteria, as over 76.7% of MLEs in Gram-negative bacteria are associated with MGEs (Fig. 5B).

We observed two distinct patterns. In Gram-positive bacteria, MLEs are inherited from ancient bacteria that may have developed resistance to naturally occurring antibiotics and spread primarily through clonal propagation. Although their origin is largely independent of human antibiotic use, these genes are more prevalent in livestock-, food-, and plant-associated bacteria, where macrolides are commonly used, suggesting that environmental exposure may promote their enrichment. In contrast, the spread of MLEs in Gram-negative bacteria is linked to inherent resistance to macrolide antibiotics. Although these bacteria were not originally expected to carry MLEs, they acquired them through HGT as a response to elevated local concentrations of antibiotics resulting from extensive human use. This suggests that Gram-negative bacteria adapted to macrolides via HGT due to antibiotic pressure.

This study provides a comprehensive overview of Gram-positive MLEs. In addition to the limitations already discussed—such as potential effects of heterologous expression and biases in available genomic data—other aspects warrant further attention. For instance, the ecological drivers shaping the distribution and persistence of MLE-carrying bacteria remain unclear, and the clinical relevance of these enzymes has yet to be systematically assessed in real infection contexts. Moreover, the evolutionary pressures contributing to the emergence and diversification of Gram-positive MLEs require deeper exploration through temporal and metagenomic analyses. Considering the interconnectedness of human, animal, and environmental reservoirs, future investigations under a One Health framework will be crucial to comprehensively understand and mitigate the spread of MLE-mediated macrolide resistance [55, 56].

## Conclusion

In the ongoing battle between humans and bacterial pathogens, understanding the spread of antibiotic resistance genes is crucial for controlling antimicrobial resistance (AMR). Our research has biochemically characterized MLEs from Gram-positive species, revealing that they are ancient antibiotic resistance genes, existing for at least 2.7 million years. MLEs are prevalent in Gram-positive bacteria, especially in *Bacillus* species, with a carrier rate exceeding 95%. Due to its ability to form spores, *Bacillus* exhibits strong environmental resilience, and its wide geographical distribution makes it a significant source of MLEs. Although only a small fraction is associated with HGT, the potential risk of dissemination still warrants further assessment. In contrast to the clonal spread seen in Gram-positive bacteria, we also found that the overuse of antibiotics by humans is likely the main factor driving the acquisition of MLEs by Gram-negative bacteria, which naturally exhibit resistance to macrolide antibiotics and initially did not carry these genes. These findings highlight the urgency of strengthening antibiotic stewardship through an integrated One Health strategy that considers the entire continuum of resistance circulation across sectors and ecosystems.

## Supplementary Material

Supplementary_material_wraf261

Supplementary_data_wraf261

## Data Availability

All protein sequences have been deposited in the NCBI database under the following accession numbers: PQ676745, PQ676746, PQ676747, PQ676748, PQ676749, and PQ67662322. All information is included in the manuscript or supplementary files.
