# Supplementary material for: Global distribution of α/β hydrolase family macrolide esterases in Gram-positive bacteria
Source: ISME J. 2025 Nov 26;19(1):wraf261. doi: 10.1093/ismejo/wraf261 (PMC12704417; doi:10.1093/ismejo/wraf261)
Supplement: Supplementary_material_wraf261 [file supplementary_material_wraf261.pdf]

Supplementary material for

Global distribution of α/β hydrolase family macrolide esterases in Gram-positive bacteria

Yang Zhou[1*], Yongqiang Yang[2*], Yuqi Mao[1*], Zhangqun Hou[1], Yiyang Xu[1], Kelei Zhao[1], Yiwen Chu[1], Xinrong Wang[1], Can Wang[3], Shun Li[4], Fei Xu[5], Likai Hao[6,7], Binbin Xie[†4], Jiafu Lin[†1], Tao Song[†1]

1: Antibiotics Research and Re-evaluation Key Laboratory of Sichuan Province, Sichuan Industrial Institute of Antibiotics, School of pharmacy, Chengdu University, Chengdu 610106, China

2: Center of Infectious Diseases, Center for Pathogen Research, West China Hospital, Sichuan University, Chengdu, China

3: State Key Laboratory of Geohazard Prevention and Geoenvironment Protection, College of Ecology and Environment, Chengdu University of Technology, Chengdu 610059, China

4: State Key Laboratory of Microbial Technology and Microbial Technology Institute, Shandong University, Qingdao 266237, China

5: Key Laboratory of Bio-Resource and Eco-Environment of Ministry of Education, College of Life Sciences, Sichuan University, Chengdu 610065, Sichuan, PR China

6: State Key Laboratory of Environmental Geochemistry, Institute of Geochemistry, CAS, Guiyang 550081, PR China

7: University of Chinese Academy of Sciences, Beijing 100049 PR China

*These authors contributed equally to this work.

†Corresponding author: Binbin Xie, State Key Laboratory of Microbial Technology and Microbial Technology Institute, Shandong University, Qingdao 266237, China. E-mails: xbb@sdu.edu.cn. Jiafu Lin, Antibiotics Research and Re-evaluation Key Laboratory of Sichuan Province, Sichuan Industrial Institute of Antibiotics, School of pharmacy, Chengdu University, Chengdu 610106, China. E-mails: linjiafu@cdu.edu.cn. Tao Song, Antibiotics Research and Re-evaluation Key Laboratory of Sichuan Province, Sichuan Industrial Institute of Antibiotics, School of pharmacy, Chengdu University, Chengdu 610106, China. E-mails: songtao@cdu.edu.cn.

Supplementary material 1

Composition of the local Gram-positive bacterial database. (A) Proportion of genomes at different assembly levels included in the database.(B) Proportion of metagenome-derived versus non-metagenome-derived genomes.(C) Relative abundance and taxonomic distribution of *Firmicutes* species in the local Gram-positive bacterial database, showing only the top 10 most abundant genera.(D) Relative abundance and taxonomic distribution of *Actinobacteria* species in the local Gram-positive bacterial database, showing only the top 10 most abundant genera.

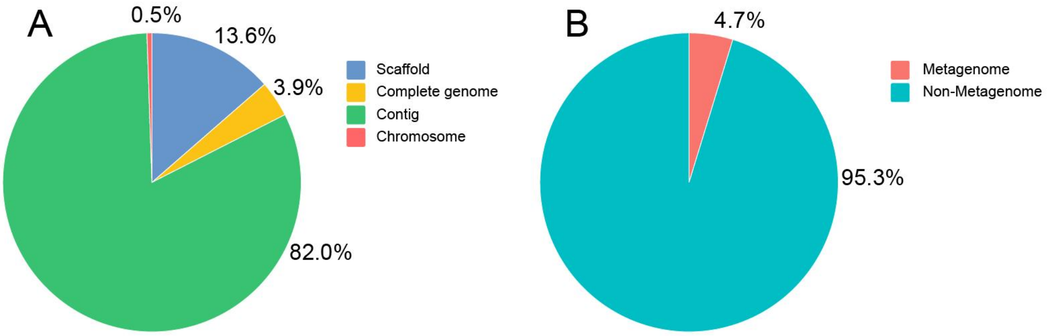

C

Genus	Proportion (%)
Streptococcus	29.3
Staphylococcus	25.1
Listeria	15.1
Enterococcus	9.5
Clostridium	5.4
Bacillus	2.0
Lactiplantibacillus	0.3
Lacticaseibacillus	0.3
Lachnospira	0.2
Lactobacillus	0.2
Other	12.6

D

Genus	Proportion (%)
Mycobacterium	14.8
Streptomyces	13.1
Bifidobacterium	7.3
Cutibacterium	3.9
Corynebacterium	3.9
Candidatus	1.4
Actinomyces	1.1
Microbacterium	1.1
Collinsella	1.0
Rhodococcus	0.8
Other	51.6

Supplementary material 2

Comparison of sequence identity between preliminarily validated protein candidates without macrolide esterases with characterized EstT and EstX enzymes.

Protein	Sequence identity with EstT	Sequence identity with EstX
WP_001083348.1	28.8%	30.8%
WP_057438337.1	27.9%	30.1%
PXX67395.1	25.1%	29.4%
MBW4008855.1	27.3%	26.7%
WP_081058018.1	27.4%	26.9%

Supplementary material 3

Natural Transformation Protocol for *Bacillus subtilis* WB800N. The recombinant plasmid was introduced into *B. subtilis* WB800N via a natural transformation protocol. Single colonies of *B. subtilis* WB800N were inoculated into freshly prepared basic medium (60 mM K_2HPO_4, 40 mM KH_2PO_4, 3 mM trisodium citrate, 20 mM potassium L-glutamate, 3 mM $MgSO_4$, 1% glucose, 20 μg/mL L-tryptophan, 0.1% casamino acids) and cultured overnight at 37°C with shaking at 250 rpm. The overnight culture was diluted to an OD 600 of 0.2 and incubated for an additional 4 hours. Cells were then harvested, resuspended in fresh basic medium, and incubated with 0.5-1.0 μg of plasmid DNA for 6 hours. Following transformation, cells were plated on LB agar containing 5 μg/mL chloramphenicol. Successful transformants were initially screened by colony PCR and subsequently verified by sequencing.

Supplementary material 4

The number and proportion of Gram-positive bacterial species harboring macrolide esterases. The proportion was calculated as the number of species carrying macrolide esterases divided by the total number of species in the dataset.

Specie	Number	Proportion (%)
Bacillus cereus	2079	26.4
Bacillus thuringiensis	1158	14.7
Bacillus anthracis	591	7.5
Priestia megaterium	382	4.9
Bacillus toyonensis	353	4.5
Bacillus mycoides	293	3.7
Bacillus paranthracis	259	3.3
Bacillus wiedmannii	233	3.0
Bacillus pseudomycoides	128	1.6
Paenibacillus polymyxa	91	1.2
Priestia aryabhattai	85	1.1
Bacillus pacificus	80	1.0
Peribacillus frigoritolerans	68	0.9
Bacillus tropicus	56	0.7
Cytobacillus firmus	40	0.5
Other	1974	25.0

Supplementary material 5

The heatmap illustrates the sequence identity among six representative macrolide esterases and EstX and EstT. Color depth indicates similarity levels, with numerical values representing pairwise similarity percentages between esterases. EstX and EstT were two characterized macrolide esterases belonging to the α/β hydrolase superfamily, originally identified in Gram-negative bacteria.

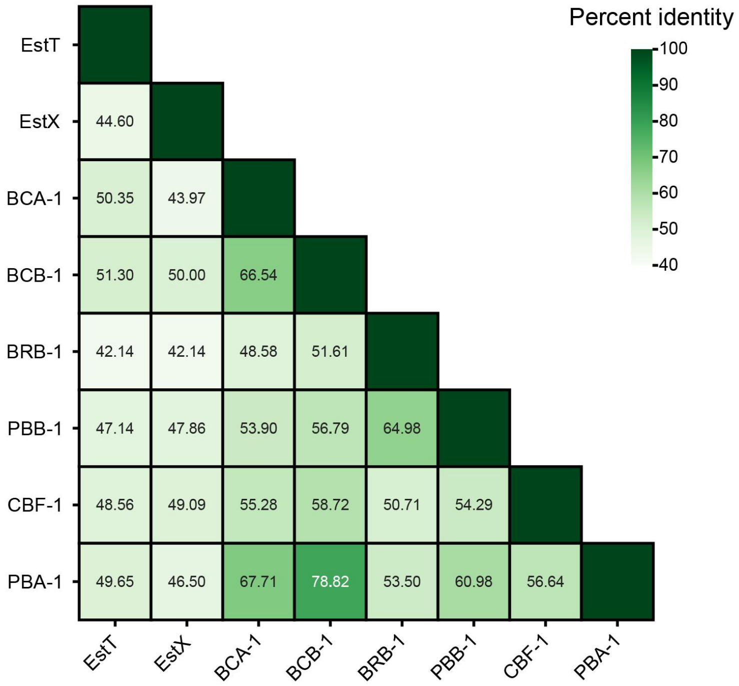

Supplementary material 6

Electrospray ionisation mass spectrometry (ESI-MS) analysis of the products from the reaction of macrolide esterase BRB-1 with eight 16-membered macrolide antibiotics (tylosin, tilmicosin, tildipirosin, kitasamycin, josamycin, spiramycin, midecamycin, acetylspiramycin). Reaction sites are marked with red circles in the compound structure. Mass spectra display antibiotic hydrolysis reaction by macrolide esterase: black peaks (parent antibiotic), red peaks (antibiotic hydrolyzed product). Annotated numbers indicate corresponding mass-to-charge ratios (*m/z*).

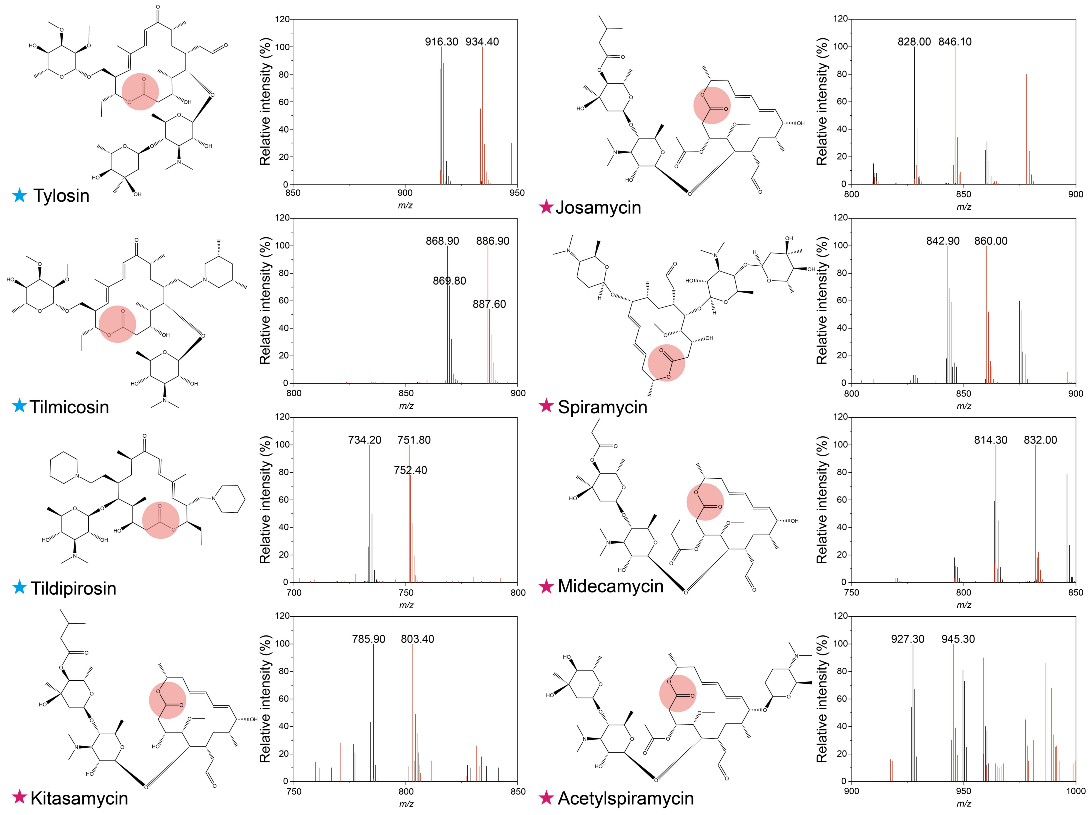

Supplementary material 7

ESI-MS analysis of the reaction products between esterase BCA-1 and 16-membered macrolide antibiotics. The degradable compound structures have their reaction sites highlighted with red circles. The mass spectra depict the esterase-catalyzed hydrolysis of the antibiotic: the upper panel shows the parent antibiotic, and the lower panel shows the hydrolysis product.

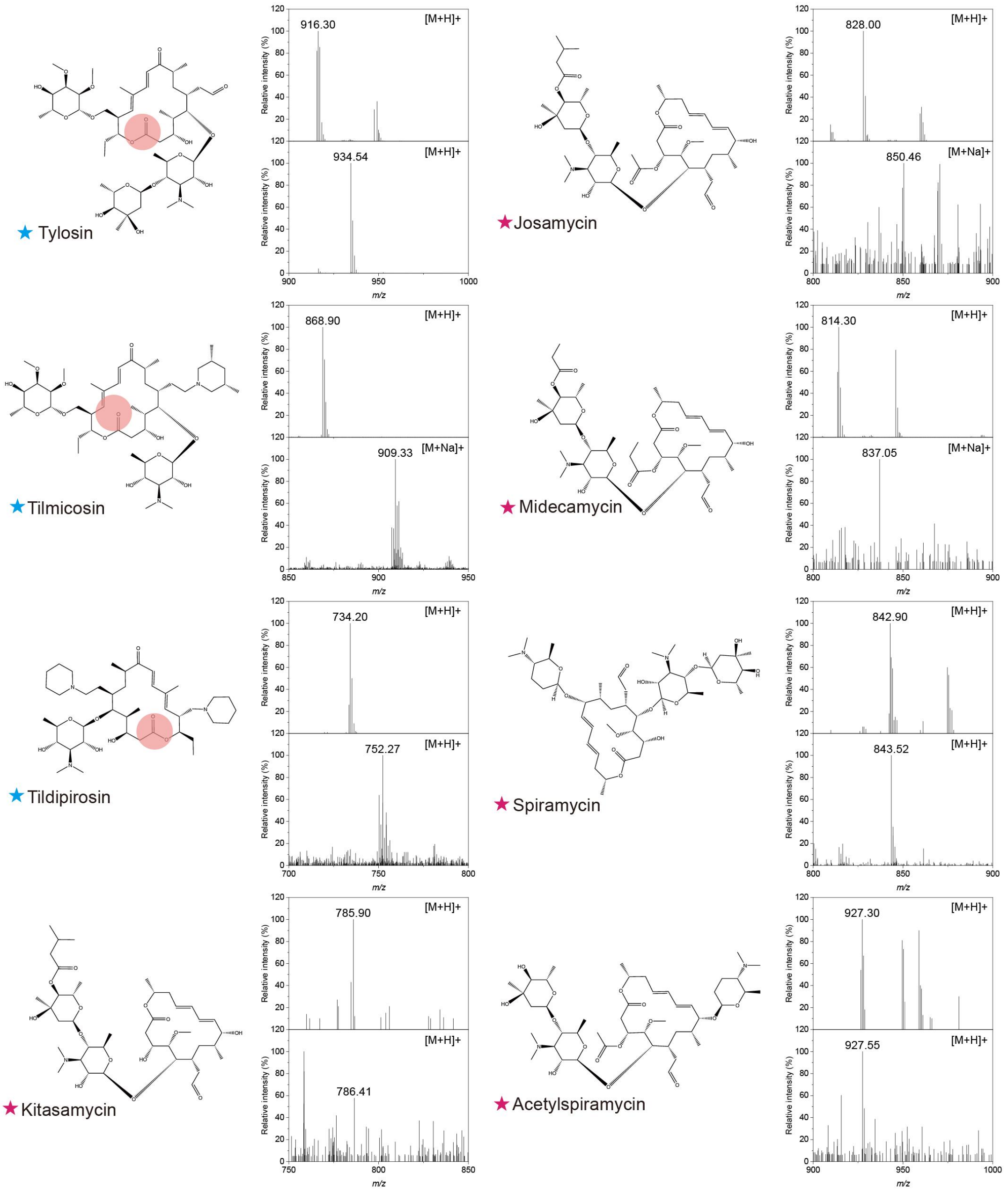

Supplementary material 8

ESI-MS analysis of the reaction products between esterase BCB-1 and 16-membered macrolide antibiotics.

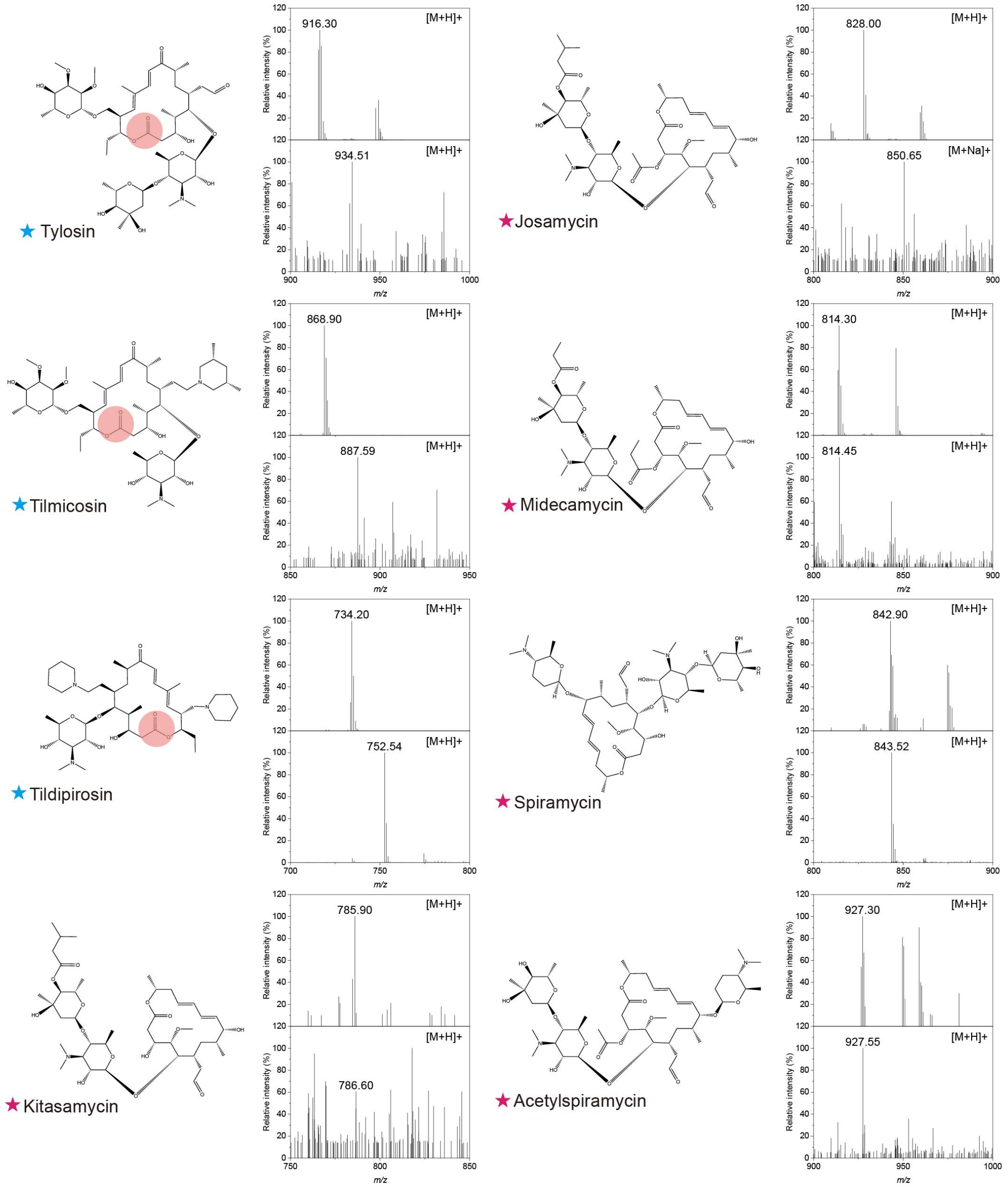

Supplementary material 9

ESI-MS analysis of the reaction products between esterase PBB-1 and 16-membered macrolide antibiotics.

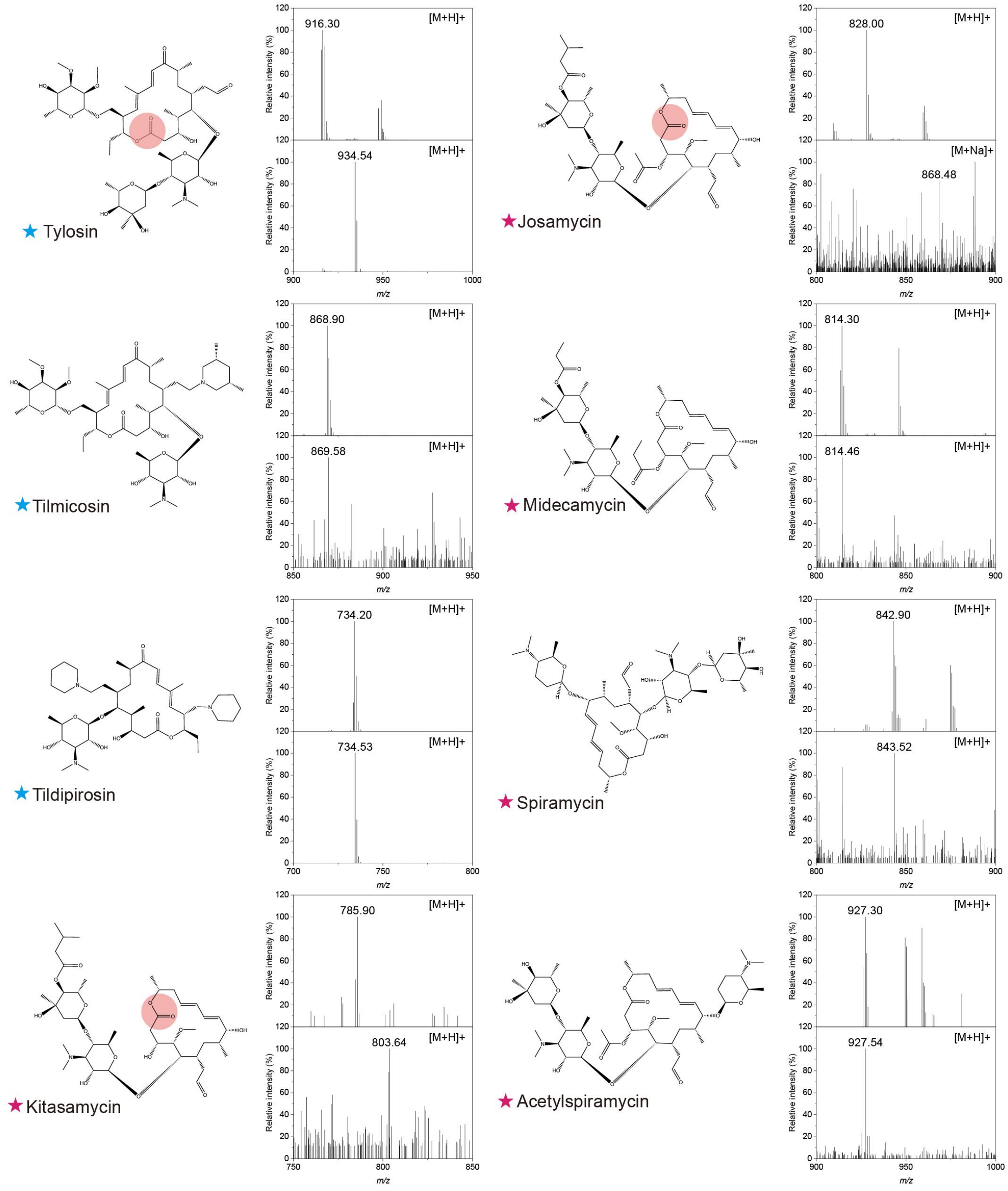

Supplementary material 10

ESI-MS analysis of the reaction products between esterase CBF-1 and 16-membered macrolide antibiotics.

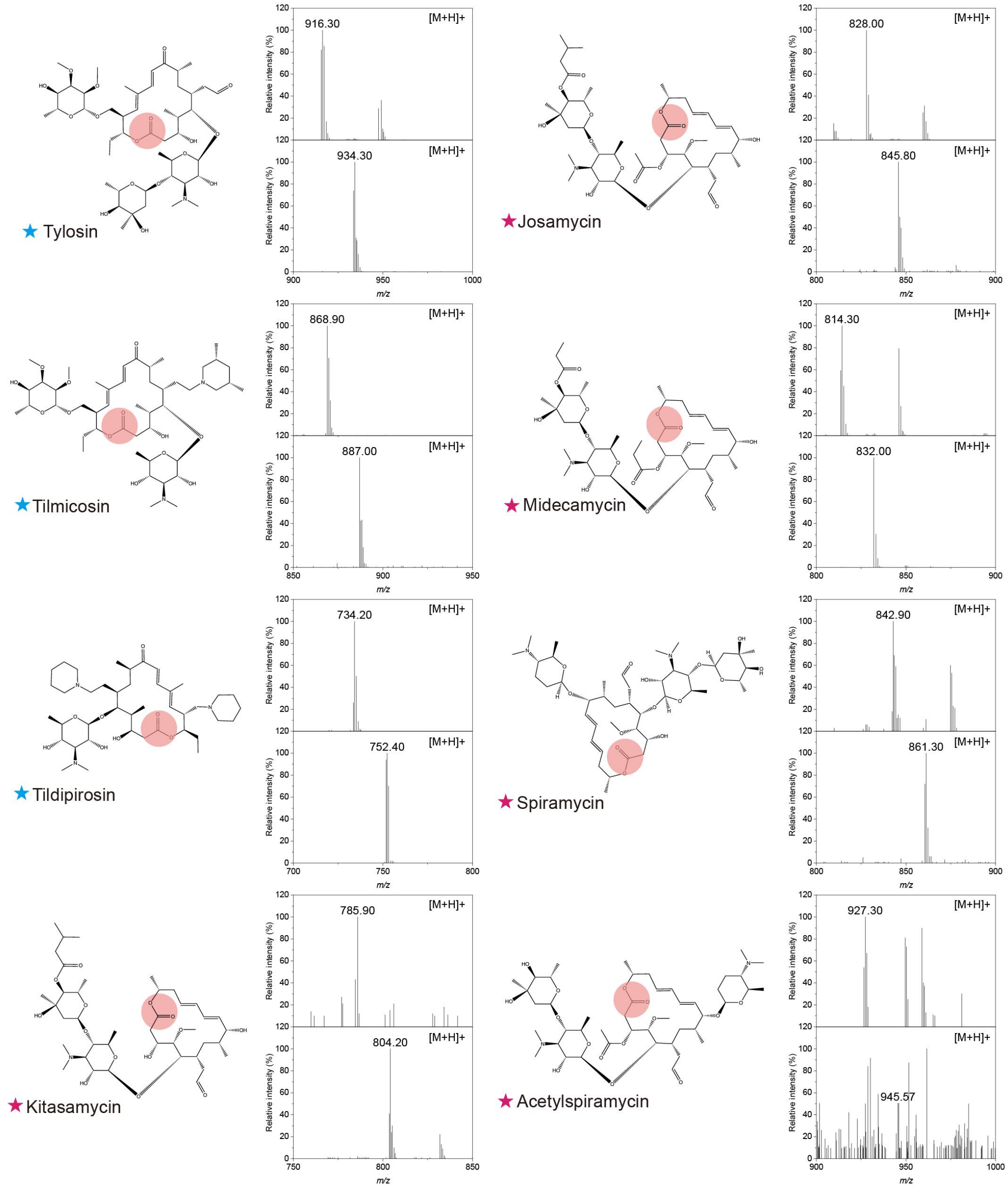

Supplementary material 11

ESI-MS analysis of the reaction products between esterase PBA-1 and 16-membered macrolide antibiotics.

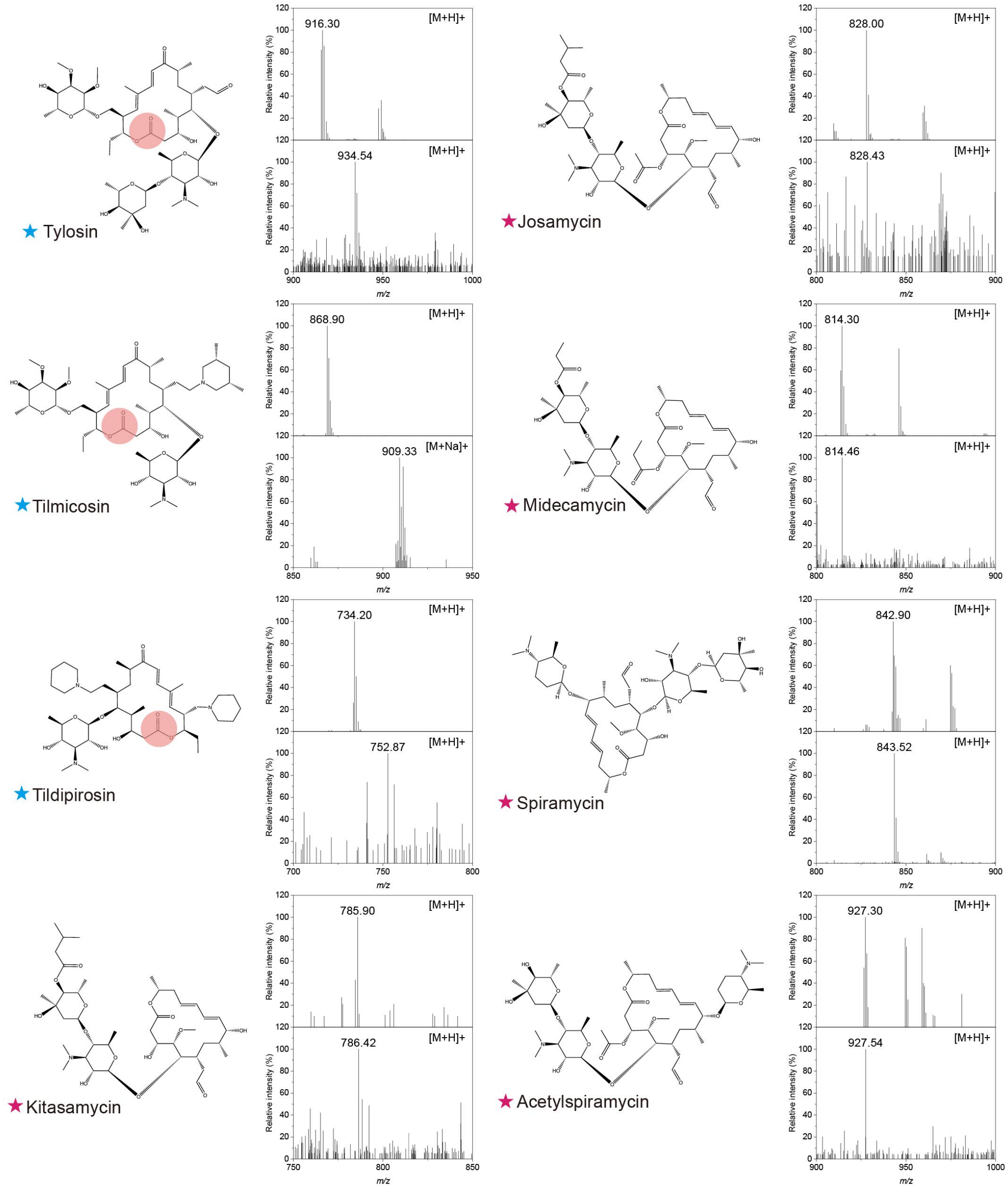

Supplementary material 12

Domain architecture of six representative macrolide esterases from Gram-positive bacteria. Green represent α/β Hydrolase Superfamily.

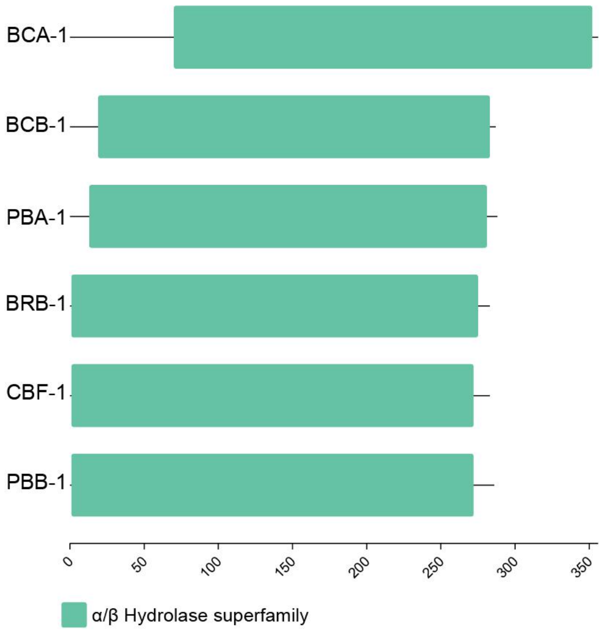

Supplementary material 13

The multiple sequence alignment of six representative esterases with EstT and EstX. Protein sequence alignment was performed using MUSCLE, with resulting alignments generated via the ESPript 3.0 online server (http://espript.ibcp.fr/ESPript/ESPript/). Identical residues across the seven proteins were shaded in red. Catalytic triad residues (Asp, Ser, His) were highlighted with red pentagrams.

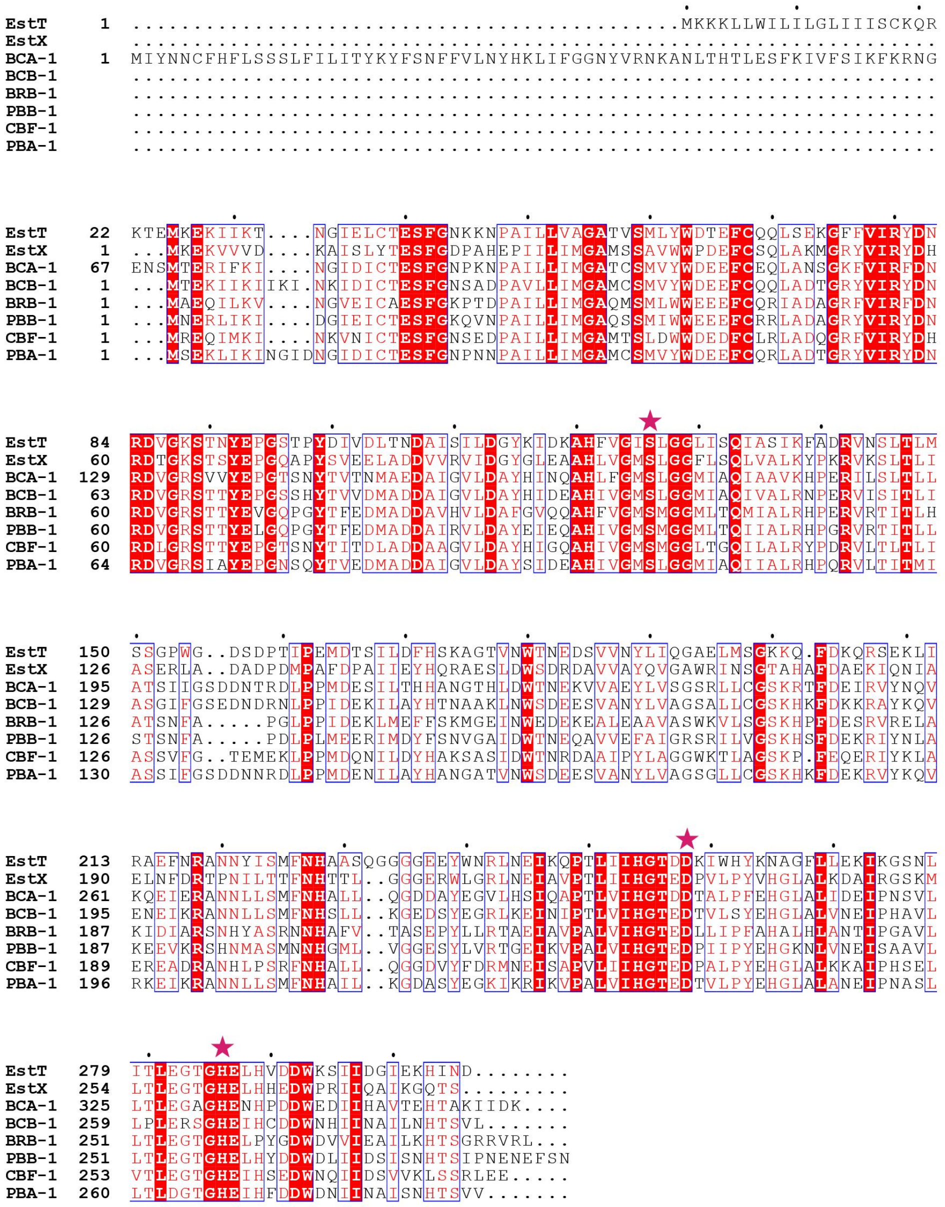

Supplementary material 14

Structural analysis of macrolide esterase. (A) Binding pockets in the macrolide esterases were predicted using PrankWeb (https://prankweb.cz/). The macrolide esterases structure was depicted in cartoon representation, with binding pocket residues rendered as a purple surface, visualized using UCSF Chimera. (B) The ConSurf analysis of macrolide esterases was displayed as a surface-rendered 3D structure, color-coded by sequence conservation scores (1–9 scale; increasing redness indicates higher conservation). Visualizations were generated using UCSF Chimera. (C) The predicted macrolide esterases structures were presented with emphasis on the catalytic triad consisting of Ser, Asp, and His residues. Hydrogen bonds were indicated by dashed lines, and numbers represent the hydrogen bond lengths (Å). Visualizations were generated using UCSF Chimera. Results for macrolide esterases BRB-1 were presented in Figure 3.

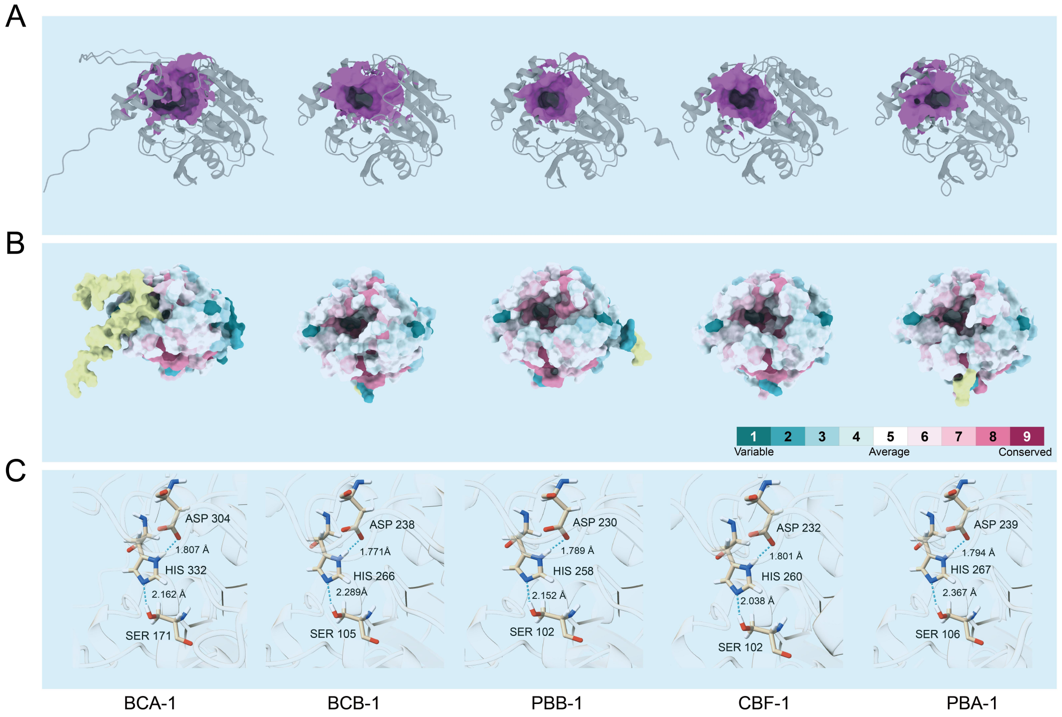

Supplementary material 15

(A) Enzyme activity of BRB-1 and mutated BRB-1 (S102A, D230A and H258A). Esterase activity was measured using p-nitrophenol as the substrate. Data are presented as the mean ± SD from independent experiments. n=3 biological replicates. (B) Minimum inhibitory concentration (MIC) analysis of *E. coli* carrying BRB-1, S102A, D230A and H258A against 8 different 16-membered macrolide antibiotics. (C) Color changes of the p-nitrophenol solution after adding enzymes. From left to right, the samples added to the wells are as follows: inactive BRB-1, BRB-1, S102A, D230A, H258A. Development of yellow color in the p-nitrophenol solution indicates esterase activity. (D-G) Inhibition zone analysis of 8 different 16-membered macrolide antibiotics after enzyme hydrolysis. The control group was conducted using inactivated enzyme. Data are presented as the mean ± SD from independent experiments. n=3 biological replicates.

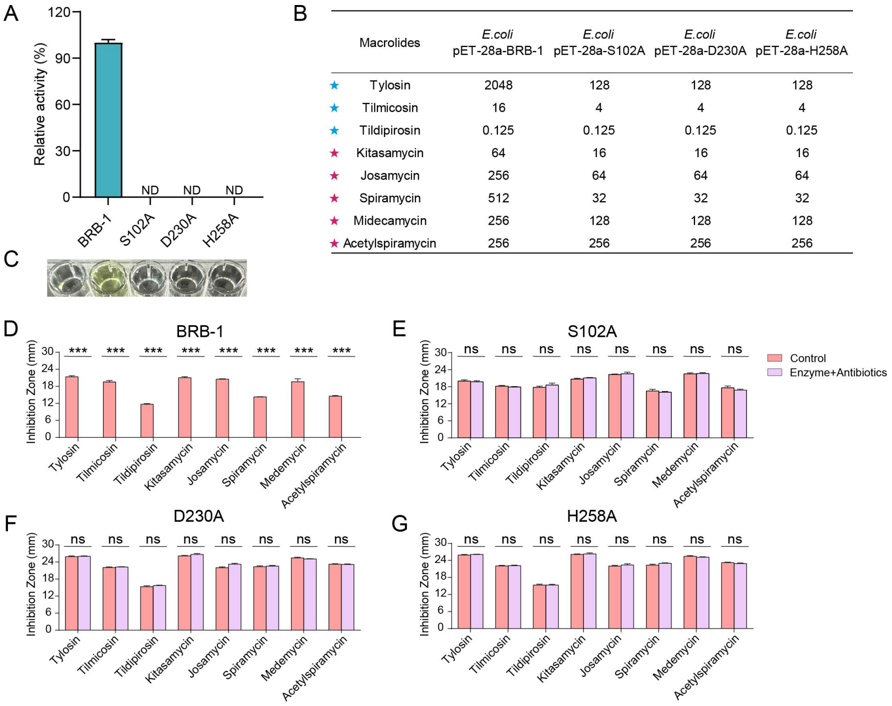

Macrolides	*E.coli* pET-28a-BRB-1	*E.coli* pET-28a-S102A	*E.coli* pET-28a-D230A	*E.coli* pET-28a-H258A
★ Tylosin	2048	128	128	128
★ Tilmicosin	16	4	4	4
★ Tildipirosin	0.125	0.125	0.125	0.125
★ Kitasamycin	64	16	16	16
★ Josamycin	256	64	64	64
★ Spiramycin	512	32	32	32
★ Midecamycin	256	128	128	128
★ Acetylspiramycin	256	256	256	256

Supplementary material 16

Distribution of Gram-positive bacterial species carrying macrolide esterases across different continents and oceans.

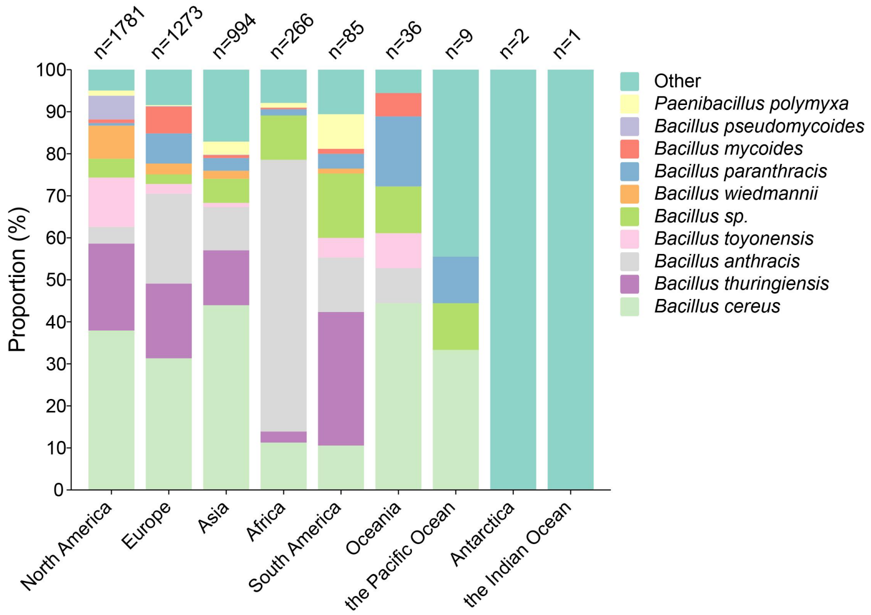

Supplementary material 17

The proportion of species containing MLE bacteria within their respective species. (A) Proportion of Gram-negative bacterial genomes carrying MLEs within each species. (B) Proportion of Gram-positive bacterial genomes carrying MLEs within each species. Only bacterial species with over 50 MLE-positive genomes were included in the analysis.

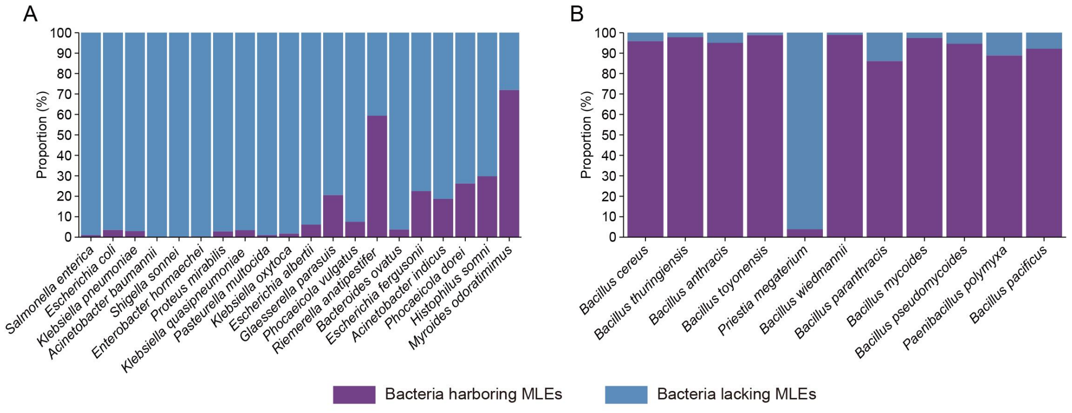

Supplementary material 18

Plasmid map of *E. coli* containing the MLE gene, using the expression vector pET-28a(+). The macrolide esterase (MLE) gene is highlighted in red. Key plasmid elements are annotated as follows: lacI, lac repressor; rop, Rop protein, which maintains the plasmid at a low copy number; bom, basis of mobility region from pBR322; ori, high-copy-number ColE1/pMB1/pBR322/pUC origin of replication; KanR, aminoglycoside phosphotransferase; f1 ori, f1 bacteriophage origin of replication (arrow indicates the direction of (+) strand synthesis); T7 terminator, transcription terminator for bacteriophage T7 RNA polymerase; 6×His, 6×His affinity tag; thrombin site, thrombin recognition and cleavage site; RBS, efficient ribosome binding site from bacteriophage T7 gene 10; T7 promoter, promoter for bacteriophage T7 RNA polymerase.

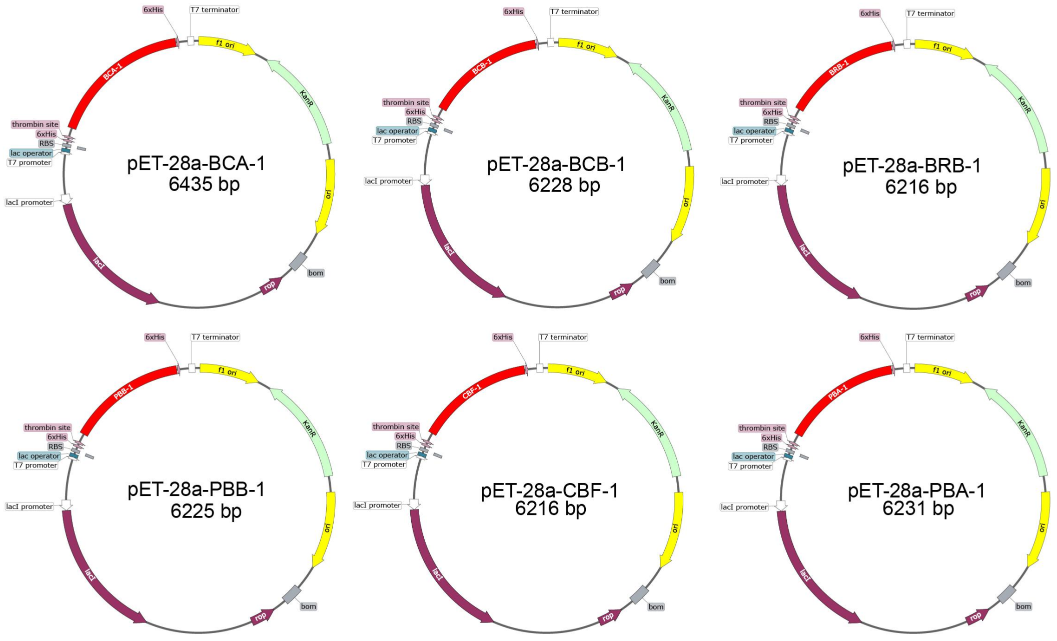

Supplementary material 19

Plasmid map of *Bacillus subtilis* containing the MLE gene, using the expression vector pHT43. The macrolide esterase (MLE) gene is highlighted in red. Key plasmid elements are annotated as follows: Pgrac, Pgrac promoter; lacI, lac repressor; ori, high-copy-number ColE1/pMB1/pBR322/pUC origin of replication; AmpR, β-lactamase gene conferring ampicillin resistance.

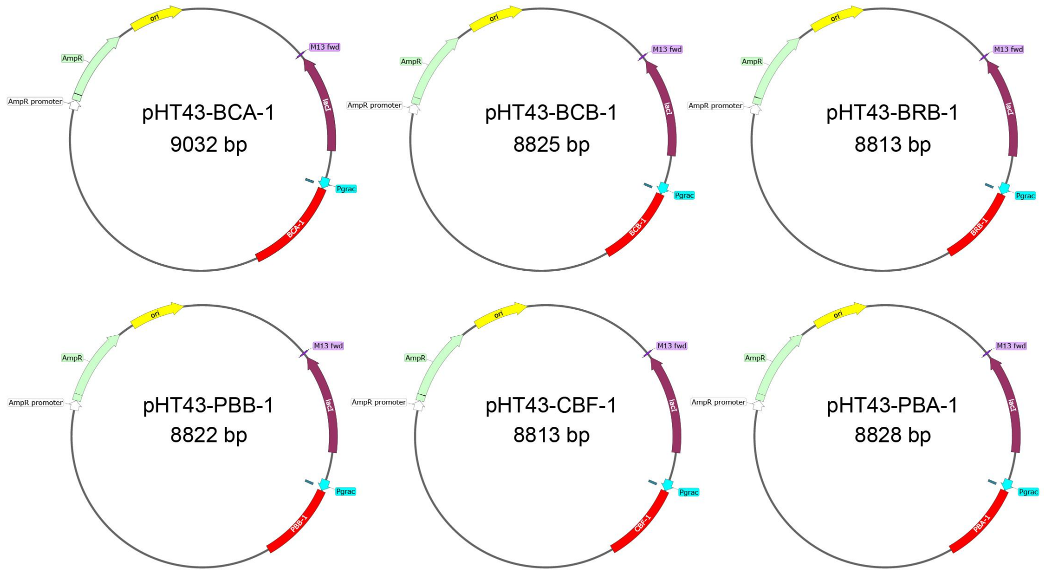

Supplementary material 20

The plasmid sequence from *E. coli* harboring the MLE gene:

pET-28a-BCA-1:

TGGCGAATGGGACGCGCCCTGTAGCGGCGCATTAAGCGCGGCGGGTGTGG

TGGTTACGCGCAGCGTGACCGCTACACTTGCCAGCGCCCTAGCGCCCGCTC

CTTTCGCTTTCTTCCCTTCCTTTCTCGCCACGTTCGCCGGCTTTCCCCGTCA

AGCTCTAAATCGGGGGCTCCCTTTAGGGTTCCGATTTAGTGCTTTACGGCAC

CTCGACCCCAAAAAACTTGATTAGGGTGATGGTTCACGTAGTGGGCCATCG

CCCTGATAGACGGTTTTTCGCCCTTTGACGTTGGAGTCCACGTTCTTTAATA

GTGGACTCTTGTTCCAAACTGGAACAACACTCAACCCTATCTCGGTCTATT

CTTTTGATTTATAAGGGATTTTGCCGATTTCGGCCTATTGGTTAAAAAATGA

GCTGATTTAACAAAAATTTAACGCGAATTTTAACAAAATATTAACGcTTACA

ATTTAGGTGGCACTTTTCGGGGAAATGTGCGCGGAACCCCTATTTGTTTATT

TTTCTAAATACATTCAAATATGTATCCGCTCATGAATTAATTCTTAGAAAAAC

TCATCGAGCATCAAATGAAACTGCAATTTATTCATATCAGGATTATCAATACC

ATATTTTTGAAAAAGCCGTTTCTGTAATGAAGGAGAAAACTCACCGAGGCA

GTTCCATAGGATGGCAAGATCCTGGTATCGGTCTGCGATTCCGACTCGTCCA

ACATCAATACAACCTATTAATTTCCCCTCGTCAAAAATAAGGTTATCAAGTG

AGAAATCACCATGAGTGACGACTGAATCCGGTGAGAATGGCAAAAGTTTAT

GCATTTCTTTCCAGACTTGTTCAACAGGCCAGCCATTACGCTCGTCATCAAA

ATCACTCGCATCAACCAAACCGTTATTCATTCGTGATTGCGCCTGAGCGAG

ACGAAATACGCGATCGCTGTTAAAAGGACAATTACAAACAGGAATCGAATG

CAACCGGCGCAGGAACACTGCCAGCGCATCAACAATATTTTCACCTGAATC

AGGATATTCTTCTAATACCTGGAATGCTGTTTTCCCGGGGATCGCAGTGGTG

AGTAACCATGCATCATCAGGAGTACGGATAAAATGCTTGATGGTCGGAAGA

GGCATAAATTCCGTCAGCCAGTTTAGTCTGACCATCTCATCTGTAACATCAT

TGGCAACGCTACCTTTGCCATGTTTCAGAAACAACTCTGGCGCATCGGGCT

TCCCATACAATCGATAGATTGTCGCACCTGATTGCCCGACATTATCGCGAGC

CCATTTATACCCATATAAATCAGCATCCATGTTGGAATTTAATCGCGGCCTAG

AGCAAGACGTTTCCCGTTGAATATGGCTCATAACACCCCTTGTATTACTGTT

TATGTAAGCAGACAGTTTTATTGTTCATGACCAAAATCCCTTAACGTGAGTT

TTCGTTCCACTGAGCGTCAGACCCCGTAGAAAAGATCAAAGGATCTTCTTG

AGATCCTTTTTTTCTGCGCGTAATCTGCTGCTTGCAAACAAAAAAACCACC

GCTACCAGCGGTGGTTTGTTTGCCGGATCAAGAGCTACCAACTCTTTTTCC

GAAGGTAACTGGCTTCAGCAGAGCGCAGATACCAAATACTGTCCTTCTAGT

GTAGCCGTAGTTAGGCCACCACTTCAAGAACTCTGTAGCACCGCCTACATA

CCTCGCTCTGCTAATCCTGTTACCAGTGGCTGCTGCCAGTGGCGATAAGTC

GTGTCTTACCGGGTTGGACTCAAGACGATAGTTACCGGATAAGGCGCAGCG

GTCGGGCTGAACGGGGGGTTCGTGCACACAGCCCAGCTTGGAGCGAACG

ACCTACACCGAACTGAGATACCTACAGCGTGAGCTATGAGAAAGCGCCAC

GCTTCCCGAAGGGAGAAAGGCGGACAGGTATCCGGTAAGCGGCAGGGTCG

GAACAGGAGAGCGCACGAGGGAGCTTCCAGGGGGAAACGCCTGGTATCTT

TATAGTCCTGTCGGGTTTCGCCACCTCTGACTTGAGCGTCGATTTTTGTGAT

GCTCGTCAGGGGGGCGGAGCCTATGGAAAAACGCCAGCAACGCGGCCTTT

TTACGGTTCCTGGCCTTTTGCTGGCCTTTTGCTCACATGTTCTTTCCTGCGTT

ATCCCCTGATTCTGTGGATAACCGTATTACCGCCTTTGAGTGAGCTGATACC

GCTCGCCGCAGCCGAACGACCGAGCGCAGCGAGTCAGTGAGCGAGGAAG

CGGAAGAGCGCCTGATGCGGTATTTTCTCCTTACGCATCTGTGCGGTATTTC

ACACCGCAATGGTGCACTCTCAGTACAATCTGCTCTGATGCCGCATAGTTAA

GCCAGTATACACTCCGCTATCGCTACGTGACTGGGTCATGGCTGCGCCCCG

ACACCCGCCAACACCCGCTGACGCGCCCTGACGGGCTTGTCTGCTCCCGG

CATCCGCTTACAGACAAGCTGTGACCGTCTCCGGGAGCTGCATGTGTCAGA

GGTTTTCACCGTCATCACCGAAACGCGCGAGGCAGCTGCGGTAAAGCTCAT

CAGCGTGGTCGTGAAGCGATTCACAGATGTCTGCCTGTTCATCCGCGTCCA

GCTCGTTGAGTTTCTCCAGAAGCGTTAATGTCTGGCTTCTGATAAAGCGGG

CCATGTTAAGGGCGGTTTTTTCCTGTTTGGTCACTGATGCCTCCGTGTAAGG

GGGATTTCTGTTCATGGGGGTAATGATACCGATGAAACGAGAGAGGATGCT

CACGATACGGGTTACTGATGATGAACATGCCCGGTTACTGGAACGTTGTGA

GGGTAAACAACTGGCGGTATGGATGCGGCGGGACCAGAGAAAAATCACTC

AGGGTCAATGCCAGCGCTTCGTTAATACAGATGTAGGTGTTCCACAGGGTA

GCCAGCAGCATCCTGCGATGCAGATCCGGAACATAATGGTGCAGGGCGCTG

ACTTCCGCGTTTCCAGACTTTACGAAACACGGAAACCGAAGACCATTCATG

TTGTTGCTCAGGTCGCAGACGTTTTGCAGCAGCAGTCGCTTCACGTTCGCT

CGCGTATCGGTGATTCATTCTGCTAACCAGTAAGGCAACCCCGCCAGCCTA

GCCGGGTCCTCAACGACAGGAGCACGATCATGCGCACCCGTGGGGCCGCC

ATGCCGGCGATAATGGCCTGCTTCTCGCCGAAACGTTTGGTGGCGGGACCA

GTGACGAAGGCTTGAGCGAGGGCGTGCAAGATTCCGAATACCGCAAGCGA

CAGGCCGATCATCGTCGCGCTCCAGCGAAAGCGGTCCTCGCCGAAAATGA

CCCAGAGCGCTGCCGGCACCTGTCCTACGAGTTGCATGATAAAGAAGACA

GTCATAAGTGCGGCGACGATAGTCATGCCCCGCGCCCACCGGAAGGAGCT

GACTGGGTTGAAGGCTCTCAAGGGCATCGGTCGAGATCCCGGTGCCTAATG

AGTGAGCTAACTTACATTAATTGCGTTGCGCTCACTGCCCGCTTTCCAGTCG

GGAAACCTGTCGTGCCAGCTGCATTAATGAATCGGCCAACGCGCGGGGAG

AGGCGGTTTGCGTATTGGGCGCCAGGGTGGTTTTTCTTTTCACCAGTGAGA

CGGGCAACAGCTGATTGCCCTTCACCGCCTGGCCCTGAGAGAGTTGCAGC

AAGCGGTCCACGCTGGTTTGCCCCAGCAGGCGAAAATCCTGTTTGATGGTG

GTTAACGGCGGGATATAACATGAGCTGTCTTCGGTATCGTCGTATCCCACTA

CCGAGATATCCGCACCAACGCGCAGCCCGGACTCGGTAATGGCGCGCATTG

CGCCCAGCGCCATCTGATCGTTGGCAACCAGCATCGCAGTGGGAACGATGC

CCTCATTCAGCATTTGCATGGTTTGTTGAAAACCGGACATGGCACTCCAGT

CGCCTTCCCGTTCCGCTATCGGCTGAATTTGATTGCGAGTGAGATATTTATG

CCAGCCAGCCAGACGCAGACGCGCCGAGACAGAACTTAATGGGCCCGCTA

ACAGCGCGATTTGCTGGTGACCCAATGCGACCAGATGCTCCACGCCCAGTC

GCGTACCGTCTTCATGGGAGAAAATAATACTGTTGATGGGTGTCTGGTCAG

AGACATCAAGAAATAACGCCGGAACATTAGTGCAGGCAGCTTCCACAGCA

ATGGCATCCTGGTCATCCAGCGGATAGTTAATGATCAGCCCACTGACGCGTT

GCGCGAGAAGATTGTGCACCGCCGCTTTACAGGCTTCGACGCCGCTTCGTT

CTACCATCGACACCACCACGCTGGCACCCAGTTGATCGGCGCGAGATTTAA

TCGCCGCGACAATTTGCGACGGCGCGTGCAGGGCCAGACTGGAGGTGGCA

ACGCCAATCAGCAACGACTGTTTGCCCGCCAGTTGTTGTGCCACGCGGTTG

GGAATGTAATTCAGCTCCGCCATCGCCGCTTCCACTTTTTCCCGCGTTTTCG

CAGAAACGTGGCTGGCCTGGTTCACCACGCGGGAAACGGTCTGATAAGAG

ACACCGGCATACTCTGCGACATCGTATAACGTTACTGGTTTCACATTCACCA

CCCTGAATTGACTCTCTTCCGGGCGCTATCATGCCATACCGCGAAAGGTTTT

GCGCCATTCGATGGTGTCCGGGATCTCGACGCTCTCCCTTATGCGACTCCTG

CATTAGGAAGCAGCCCAGTAGTAGGTTGAGGCCGTTGAGCACCGCCGCCG

CAAGGAATGGTGCATGCAAGGAGATGGCGCCCAACAGTCCCCCGGCCACG

GGGCCTGCCACCATACCCACGCCGAAACAAGCGCTCATGAGCCCGAAGTG

GCGAGCCCGATCTTCCCCATCGGTGATGTCGGCGATATAGGCGCCAGCAAC

CGCACCTGTGGCGCCGGTGATGCCGGCCACGATGCGTCCGGCGTAGAGGA

TCGAGATCTCGATCCCGCGAAATTAATACGACTCACTATAGGGGAATTGTGA

GCGGATAACAATTCCCCTCTAGAAATAATTTTGTTTAACTTTAAGAAGGAGA

TATACCATGGGCAGCAGCCATCATCATCATCATCACAGCAGCGGCCTGGTGC

CGCGCGGCAGCCATATGGCTAGCATGACTGGTGGACAGCAAATGGGTCGC

GGATCCGAATTCGAGCTCCGTCGACAAGCTTGCATGATCTACAACAACTGC

TTCCATTTCCTGAGCAGTAGTCTGTTTATTCTGATTACCTACAAGTACTTCAG

CAACTTTTTCGTTCTGAACTACCATAAGCTGATCTTTGGCGGTAATTATGTG

CGCAATAAGGCAAATCTGACCCATACCCTGGAAAGTTTTAAAATTGTGTTC

AGCATCAAGTTCAAGCGTAATGGTGAAAATAGCATGACCGAACGTATTTTT

AAGATCAACGGCATTGACATCTGCACCGAAAGTTTTGGTAATCCGAAAAAT

CCGGCAATTCTGCTGATTATGGGTGCAACCTGCAGTATGGTGTATTGGGATG

AAGAATTTTGCGAACAGCTGGCCAATAGCGGCAAATTTGTGATTCGTTTTG

ACAATCGTGACGTTGGCCGCAGCGTTGTTTATGAACCGGGTACCAGCAATT

ATACCGTTACCAATATGGCAGAAGACGCCATTGGTGTTCTGGATGCCTATCA

TATTAACCAGGCACATCTGTTTGGTATGAGTCTGGGCGGTATGATTGCCCAG

ATTGCCGCAGTTAAACATCCGGAACGCATTCTGAGCCTGACCCTGCTGGCC

ACAAGCATTATTGGTAGCGATGATAATACCCGTGATCTGCCGCCGATGGATG

AAAGTATTCTGACCCATCATGCCAATGGTACCCATCTGGATTGGACCAATGA

AAAAGTGGTTGCAGAATATCTGGTGAGCGGTAGCCGCCTGCTGTGTGGTTC

AAAACGCACCTTTGATGAAATTCGTGTTTACAATCAGGTGAAGCAGGAAAT

TGAACGTGCCAATAATCTGCTGAGCATGTTTAATCATGCGCTGCTGCAGGGT

GACGATGCCTATGAAGGCGTTCTGCATAGCATTCAGGCCCCGACCCTGGTTA

TTCATGGTACCGATGATACCGCCCTGCCGTTTGAACATGGCCTGGCACTGAT

TGATGAAATTCCGAATAGCGTTCTGCTGACCCTGGAAGGCGCCGGTCATGA

AAATCATCCGGATGATTGGGAAGATATTATCCATGCCGTTACCGAACATACC

GCCAAAATTATTGATAAGGCGGCCGCACTCGAGCACCACCACCACCACCAC

TGAGATCCGGCTGCTAACAAAGCCCGAAAGGAAGCTGAGTTGGCTGCTGC

CACCGCTGAGCAATAACTAGCATAACCCCTTGGGGCCTCTAAACGGGTCTT

GAGGGGTTTTTTGCTGAAAGGAGGAACTATATCCGGAT

pET-28a-BCB-1:

TGGCGAATGGGACGCGCCCTGTAGCGGCGCATTAAGCGCGGCGGGTGTGG

TGGTTACGCGCAGCGTGACCGCTACACTTGCCAGCGCCCTAGCGCCCGCTC

CTTTCGCTTTCTTCCCTTCCTTTCTCGCCACGTTCGCCGGCTTTCCCCGTCA

AGCTCTAAATCGGGGGCTCCCTTTAGGGTTCCGATTTAGTGCTTTACGGCAC

CTCGACCCCAAAAAACTTGATTAGGGTGATGGTTCACGTAGTGGGCCATCG

CCCTGATAGACGGTTTTTCGCCCTTTGACGTTGGAGTCCACGTTCTTTAATA

GTGGACTCTTGTTCCAAACTGGAACAACACTCAACCCTATCTCGGTCTATT

CTTTTGATTTATAAGGGATTTTGCCGATTTCGGCCTATTGGTTAAAAAATGA

GCTGATTTAACAAAAATTTAACGCGAATTTTAACAAAATATTAACGcTTACA

ATTTAGGTGGCACTTTTCGGGGAAATGTGCGCGGAACCCCTATTTGTTTATT

TTTCTAAATACATTCAAATATGTATCCGCTCATGAATTAATTCTTAGAAAAAC

TCATCGAGCATCAAATGAAACTGCAATTTATTCATATCAGGATTATCAATACC

ATATTTTTGAAAAAGCCGTTTCTGTAATGAAGGAGAAAACTCACCGAGGCA

GTTCCATAGGATGGCAAGATCCTGGTATCGGTCTGCGATTCCGACTCGTCCA

ACATCAATACAACCTATTAATTTCCCCTCGTCAAAAATAAGGTTATCAAGTG

AGAAATCACCATGAGTGACGACTGAATCCGGTGAGAATGGCAAAAGTTTAT

GCATTTCTTTCCAGACTTGTTCAACAGGCCAGCCATTACGCTCGTCATCAAA

ATCACTCGCATCAACCAAACCGTTATTCATTCGTGATTGCGCCTGAGCGAG

ACGAAATACGCGATCGCTGTTAAAAGGACAATTACAAACAGGAATCGAATG

CAACCGGCGCAGGAACACTGCCAGCGCATCAACAATATTTTCACCTGAATC

AGGATATTCTTCTAATACCTGGAATGCTGTTTTCCCGGGGATCGCAGTGGTG

AGTAACCATGCATCATCAGGAGTACGGATAAAATGCTTGATGGTCGGAAGA

GGCATAAATTCCGTCAGCCAGTTTAGTCTGACCATCTCATCTGTAACATCAT

TGGCAACGCTACCTTTGCCATGTTTCAGAAACAACTCTGGCGCATCGGGCT

TCCCATACAATCGATAGATTGTCGCACCTGATTGCCCGACATTATCGCGAGC

CCATTTATACCCATATAAATCAGCATCCATGTTGGAATTTAATCGCGGCCTAG

AGCAAGACGTTTCCCGTTGAATATGGCTCATAACACCCCTTGTATTACTGTT

TATGTAAGCAGACAGTTTTATTGTTCATGACCAAAATCCCTTAACGTGAGTT

TTCGTTCCACTGAGCGTCAGACCCCGTAGAAAAGATCAAAGGATCTTCTTG

AGATCCTTTTTTTCTGCGCGTAATCTGCTGCTTGCAAACAAAAAAACCACC

GCTACCAGCGGTGGTTTGTTTGCCGGATCAAGAGCTACCAACTCTTTTTCC

GAAGGTAACTGGCTTCAGCAGAGCGCAGATACCAAATACTGTCCTTCTAGT

GTAGCCGTAGTTAGGCCACCACTTCAAGAACTCTGTAGCACCGCCTACATA

CCTCGCTCTGCTAATCCTGTTACCAGTGGCTGCTGCCAGTGGCGATAAGTC

GTGTCTTACCGGGTTGGACTCAAGACGATAGTTACCGGATAAGGCGCAGCG

GTCGGGCTGAACGGGGGGTTCGTGCACACAGCCCAGCTTGGAGCGAACG

ACCTACACCGAACTGAGATACCTACAGCGTGAGCTATGAGAAAGCGCCAC

GCTTCCCGAAGGGAGAAAGGCGGACAGGTATCCGGTAAGCGGCAGGGTCG

GAACAGGAGAGCGCACGAGGGAGCTTCCAGGGGGAAACGCCTGGTATCTT

TATAGTCCTGTCGGGTTTCGCCACCTCTGACTTGAGCGTCGATTTTTGTGAT

GCTCGTCAGGGGGGCGGAGCCTATGGAAAAACGCCAGCAACGCGGCCTTT

TTACGGTTCCTGGCCTTTTGCTGGCCTTTTGCTCACATGTTCTTTCCTGCGTT

ATCCCCTGATTCTGTGGATAACCGTATTACCGCCTTTGAGTGAGCTGATACC

GCTCGCCGCAGCCGAACGACCGAGCGCAGCGAGTCAGTGAGCGAGGAAG

CGGAAGAGCGCCTGATGCGGTATTTTCTCCTTACGCATCTGTGCGGTATTTC

ACACCGCAATGGTGCACTCTCAGTACAATCTGCTCTGATGCCGCATAGTTAA

GCCAGTATACACTCCGCTATCGCTACGTGACTGGGTCATGGCTGCGCCCCG

ACACCCGCCAACACCCGCTGACGCGCCCTGACGGGCTTGTCTGCTCCCGG

CATCCGCTTACAGACAAGCTGTGACCGTCTCCGGGAGCTGCATGTGTCAGA

GGTTTTCACCGTCATCACCGAAACGCGCGAGGCAGCTGCGGTAAAGCTCAT

CAGCGTGGTCGTGAAGCGATTCACAGATGTCTGCCTGTTCATCCGCGTCCA

GCTCGTTGAGTTTCTCCAGAAGCGTTAATGTCTGGCTTCTGATAAAGCGGG

CCATGTTAAGGGCGGTTTTTTCCTGTTTGGTCACTGATGCCTCCGTGTAAGG

GGGATTTCTGTTCATGGGGGTAATGATACCGATGAAACGAGAGAGGATGCT

CACGATACGGGTTACTGATGATGAACATGCCCGGTTACTGGAACGTTGTGA

GGGTAAACAACTGGCGGTATGGATGCGGCGGGACCAGAGAAAAATCACTC

AGGGTCAATGCCAGCGCTTCGTTAATACAGATGTAGGTGTTCCACAGGGTA

GCCAGCAGCATCCTGCGATGCAGATCCGGAACATAATGGTGCAGGGCGCTG

ACTTCCGCGTTTCCAGACTTTACGAAACACGGAAACCGAAGACCATTCATG

TTGTTGCTCAGGTCGCAGACGTTTTGCAGCAGCAGTCGCTTCACGTTCGCT

CGCGTATCGGTGATTCATTCTGCTAACCAGTAAGGCAACCCCGCCAGCCTA

GCCGGGTCCTCAACGACAGGAGCACGATCATGCGCACCCGTGGGGCCGCC

ATGCCGGCGATAATGGCCTGCTTCTCGCCGAAACGTTTGGTGGCGGGACCA

GTGACGAAGGCTTGAGCGAGGGCGTGCAAGATTCCGAATACCGCAAGCGA

CAGGCCGATCATCGTCGCGCTCCAGCGAAAGCGGTCCTCGCCGAAAATGA

CCCAGAGCGCTGCCGGCACCTGTCCTACGAGTTGCATGATAAAGAAGACA

GTCATAAGTGCGGCGACGATAGTCATGCCCCGCGCCCACCGGAAGGAGCT

GACTGGGTTGAAGGCTCTCAAGGGCATCGGTCGAGATCCCGGTGCCTAATG

AGTGAGCTAACTTACATTAATTGCGTTGCGCTCACTGCCCGCTTTCCAGTCG

GGAAACCTGTCGTGCCAGCTGCATTAATGAATCGGCCAACGCGCGGGGAG

AGGCGGTTTGCGTATTGGGCGCCAGGGTGGTTTTTCTTTTCACCAGTGAGA

CGGGCAACAGCTGATTGCCCTTCACCGCCTGGCCCTGAGAGAGTTGCAGC

AAGCGGTCCACGCTGGTTTGCCCCAGCAGGCGAAAATCCTGTTTGATGGTG

GTTAACGGCGGGATATAACATGAGCTGTCTTCGGTATCGTCGTATCCCACTA

CCGAGATATCCGCACCAACGCGCAGCCCGGACTCGGTAATGGCGCGCATTG

CGCCCAGCGCCATCTGATCGTTGGCAACCAGCATCGCAGTGGGAACGATGC

CCTCATTCAGCATTTGCATGGTTTGTTGAAAACCGGACATGGCACTCCAGT

CGCCTTCCCGTTCCGCTATCGGCTGAATTTGATTGCGAGTGAGATATTTATG

CCAGCCAGCCAGACGCAGACGCGCCGAGACAGAACTTAATGGGCCCGCTA

ACAGCGCGATTTGCTGGTGACCCAATGCGACCAGATGCTCCACGCCCAGTC

GCGTACCGTCTTCATGGGAGAAAATAATACTGTTGATGGGTGTCTGGTCAG

AGACATCAAGAAATAACGCCGGAACATTAGTGCAGGCAGCTTCCACAGCA

ATGGCATCCTGGTCATCCAGCGGATAGTTAATGATCAGCCCACTGACGCGTT

GCGCGAGAAGATTGTGCACCGCCGCTTTACAGGCTTCGACGCCGCTTCGTT

CTACCATCGACACCACCACGCTGGCACCCAGTTGATCGGCGCGAGATTTAA

TCGCCGCGACAATTTGCGACGGCGCGTGCAGGGCCAGACTGGAGGTGGCA

ACGCCAATCAGCAACGACTGTTTGCCCGCCAGTTGTTGTGCCACGCGGTTG

GGAATGTAATTCAGCTCCGCCATCGCCGCTTCCACTTTTTCCCGCGTTTTCG

CAGAAACGTGGCTGGCCTGGTTCACCACGCGGGAAACGGTCTGATAAGAG

ACACCGGCATACTCTGCGACATCGTATAACGTTACTGGTTTCACATTCACCA

CCCTGAATTGACTCTCTTCCGGGCGCTATCATGCCATACCGCGAAAGGTTTT

GCGCCATTCGATGGTGTCCGGGATCTCGACGCTCTCCCTTATGCGACTCCTG

CATTAGGAAGCAGCCCAGTAGTAGGTTGAGGCCGTTGAGCACCGCCGCCG

CAAGGAATGGTGCATGCAAGGAGATGGCGCCCAACAGTCCCCCGGCCACG

GGGCCTGCCACCATACCCACGCCGAAACAAGCGCTCATGAGCCCGAAGTG

GCGAGCCCGATCTTCCCCATCGGTGATGTCGGCGATATAGGCGCCAGCAAC

CGCACCTGTGGCGCCGGTGATGCCGGCCACGATGCGTCCGGCGTAGAGGA

TCGAGATCTCGATCCCGCGAAATTAATACGACTCACTATAGGGGAATTGTGA

GCGGATAACAATTCCCCTCTAGAAATAATTTTGTTTAACTTTAAGAAGGAGA

TATACCATGGGCAGCAGCCATCATCATCATCATCACAGCAGCGGCCTGGTGC

CGCGCGGCAGCCATATGGCTAGCATGACTGGTGGACAGCAAATGGGTCGC

GGATCCGAATTCGAGCTCCGTCGACAAGCTTGCATGACCGAAAAGATCATC

AAGATCATCAAAATCAACAAGATCGACATCTGCACCGAAAGTTTTGGTAAT

AGCGCAGATCCGGCAGTTCTGCTGATTATGGGCGCCATGTGTAGCATGGTGT

ATTGGGATGAAGAATTTTGTCAGCAGCTGGCAGATACCGGTCGCTATGTTAT

TCGTTATGATAATCGCGATGTGGGCCGTAGCACCACCTATGAACCGGGTAGT

AGTCATTATACCGTTGTTGATATGGCAGATGATGCAATTGGCGTTCTGGATG

CATATCATATTGATGAAGCACATATCGTGGGTATGAGCCTGGGCGGCATGAT

TGCACAGATTGTTGCACTGCGTAATCCGGAACGCGTGATTAGTATTACCCTG

ATTGCAAGTGGTATCTTTGGTAGTGAAGATAATGACCGTAACCTGCCGCCGA

TTGATGAAAAAATTCTGGCATATCACACCAACGCAGCCAAACTGAATTGGA

GTGATGAAGAAAGTGTGGCCAATTATCTGGTGGCAGGCAGCGCCCTGCTGT

GTGGTAGCAAACATAAATTTGATAAGAAGCGCGCATACAAGCAGGTTGAAA

ATGAAATTAAGCGCGCCAATAACCTGCTGAGTATGTTTAATCATAGCCTGCT

GAAAGGCGAAGATAGCTATGAAGGTCGCCTGAAAGAAATTAATATCCCGAC

CCTGGTTATCCATGGTACCGAAGATACCGTGCTGAGCTATGAACATGGTCTG

GCCCTGGTGAATGAAATTCCGCATGCAGTGCTGCTGCCGCTGGAAAGAAG

TGGCCATGAAATTCATTGCGATGATTGGAATCACATCATCAATGCAATCCTG

AATCACACCAGCGTGCTGGCGGCCGCACTCGAGCACCACCACCACCACCA

CTGAGATCCGGCTGCTAACAAAGCCCGAAAGGAAGCTGAGTTGGCTGCTG

CCACCGCTGAGCAATAACTAGCATAACCCCTTGGGGCCTCTAAACGGGTCT

TGAGGGGTTTTTTGCTGAAAGGAGGAACTATATCCGGAT

pET-28a-BRB-1:

TGGCGAATGGGACGCGCCCTGTAGCGGCGCATTAAGCGCGGCGGGTGTGG

TGGTTACGCGCAGCGTGACCGCTACACTTGCCAGCGCCCTAGCGCCCGCTC

CTTTCGCTTTCTTCCCTTCCTTTCTCGCCACGTTCGCCGGCTTTCCCCGTCA

AGCTCTAAATCGGGGGCTCCCTTTAGGGTTCCGATTTAGTGCTTTACGGCAC

CTCGACCCCAAAAAACTTGATTAGGGTGATGGTTCACGTAGTGGGCCATCG

CCCTGATAGACGGTTTTTCGCCCTTTGACGTTGGAGTCCACGTTCTTTAATA

GTGGACTCTTGTTCCAAACTGGAACAACACTCAACCCTATCTCGGTCTATT

CTTTTGATTTATAAGGGATTTTGCCGATTTCGGCCTATTGGTTAAAAAATGA

GCTGATTTAACAAAAATTTAACGCGAATTTTAACAAAATATTAACGcTTACA

ATTTAGGTGGCACTTTTCGGGGAAATGTGCGCGGAACCCCTATTTGTTTATT

TTTCTAAATACATTCAAATATGTATCCGCTCATGAATTAATTCTTAGAAAAAC

TCATCGAGCATCAAATGAAACTGCAATTTATTCATATCAGGATTATCAATACC

ATATTTTTGAAAAAGCCGTTTCTGTAATGAAGGAGAAAACTCACCGAGGCA

GTTCCATAGGATGGCAAGATCCTGGTATCGGTCTGCGATTCCGACTCGTCCA

ACATCAATACAACCTATTAATTTCCCCTCGTCAAAAATAAGGTTATCAAGTG

AGAAATCACCATGAGTGACGACTGAATCCGGTGAGAATGGCAAAAGTTTAT

GCATTTCTTTCCAGACTTGTTCAACAGGCCAGCCATTACGCTCGTCATCAAA

ATCACTCGCATCAACCAAACCGTTATTCATTCGTGATTGCGCCTGAGCGAG

ACGAAATACGCGATCGCTGTTAAAAGGACAATTACAAACAGGAATCGAATG

CAACCGGCGCAGGAACACTGCCAGCGCATCAACAATATTTTCACCTGAATC

AGGATATTCTTCTAATACCTGGAATGCTGTTTTCCCGGGGATCGCAGTGGTG

AGTAACCATGCATCATCAGGAGTACGGATAAAATGCTTGATGGTCGGAAGA

GGCATAAATTCCGTCAGCCAGTTTAGTCTGACCATCTCATCTGTAACATCAT

TGGCAACGCTACCTTTGCCATGTTTCAGAAACAACTCTGGCGCATCGGGCT

TCCCATACAATCGATAGATTGTCGCACCTGATTGCCCGACATTATCGCGAGC

CCATTTATACCCATATAAATCAGCATCCATGTTGGAATTTAATCGCGGCCTAG

AGCAAGACGTTTCCCGTTGAATATGGCTCATAACACCCCTTGTATTACTGTT

TATGTAAGCAGACAGTTTTATTGTTCATGACCAAAATCCCTTAACGTGAGTT

TTCGTTCCACTGAGCGTCAGACCCCGTAGAAAAGATCAAAGGATCTTCTTG

AGATCCTTTTTTTCTGCGCGTAATCTGCTGCTTGCAAACAAAAAAACCACC

GCTACCAGCGGTGGTTTGTTTGCCGGATCAAGAGCTACCAACTCTTTTTCC

GAAGGTAACTGGCTTCAGCAGAGCGCAGATACCAAATACTGTCCTTCTAGT

GTAGCCGTAGTTAGGCCACCACTTCAAGAACTCTGTAGCACCGCCTACATA

CCTCGCTCTGCTAATCCTGTTACCAGTGGCTGCTGCCAGTGGCGATAAGTC

GTGTCTTACCGGGTTGGACTCAAGACGATAGTTACCGGATAAGGCGCAGCG

GTCGGGCTGAACGGGGGGTTCGTGCACACAGCCCAGCTTGGAGCGAACG

ACCTACACCGAACTGAGATACCTACAGCGTGAGCTATGAGAAAGCGCCAC

GCTTCCCGAAGGGAGAAAGGCGGACAGGTATCCGGTAAGCGGCAGGGTCG

GAACAGGAGAGCGCACGAGGGAGCTTCCAGGGGGAAACGCCTGGTATCTT

TATAGTCCTGTCGGGTTTCGCCACCTCTGACTTGAGCGTCGATTTTTGTGAT

GCTCGTCAGGGGGGCGGAGCCTATGGAAAAACGCCAGCAACGCGGCCTTT

TTACGGTTCCTGGCCTTTTGCTGGCCTTTTGCTCACATGTTCTTTCCTGCGTT

ATCCCCTGATTCTGTGGATAACCGTATTACCGCCTTTGAGTGAGCTGATACC

GCTCGCCGCAGCCGAACGACCGAGCGCAGCGAGTCAGTGAGCGAGGAAG

CGGAAGAGCGCCTGATGCGGTATTTTCTCCTTACGCATCTGTGCGGTATTTC

ACACCGCAATGGTGCACTCTCAGTACAATCTGCTCTGATGCCGCATAGTTAA

GCCAGTATACACTCCGCTATCGCTACGTGACTGGGTCATGGCTGCGCCCCG

ACACCCGCCAACACCCGCTGACGCGCCCTGACGGGCTTGTCTGCTCCCGG

CATCCGCTTACAGACAAGCTGTGACCGTCTCCGGGAGCTGCATGTGTCAGA

GGTTTTCACCGTCATCACCGAAACGCGCGAGGCAGCTGCGGTAAAGCTCAT

CAGCGTGGTCGTGAAGCGATTCACAGATGTCTGCCTGTTCATCCGCGTCCA

GCTCGTTGAGTTTCTCCAGAAGCGTTAATGTCTGGCTTCTGATAAAGCGGG

CCATGTTAAGGGCGGTTTTTTCCTGTTTGGTCACTGATGCCTCCGTGTAAGG

GGGATTTCTGTTCATGGGGGTAATGATACCGATGAAACGAGAGAGGATGCT

CACGATACGGGTTACTGATGATGAACATGCCCGGTTACTGGAACGTTGTGA

GGGTAAACAACTGGCGGTATGGATGCGGCGGGACCAGAGAAAAATCACTC

AGGGTCAATGCCAGCGCTTCGTTAATACAGATGTAGGTGTTCCACAGGGTA

GCCAGCAGCATCCTGCGATGCAGATCCGGAACATAATGGTGCAGGGCGCTG

ACTTCCGCGTTTCCAGACTTTACGAAACACGGAAACCGAAGACCATTCATG

TTGTTGCTCAGGTCGCAGACGTTTTGCAGCAGCAGTCGCTTCACGTTCGCT

CGCGTATCGGTGATTCATTCTGCTAACCAGTAAGGCAACCCCGCCAGCCTA

GCCGGGTCCTCAACGACAGGAGCACGATCATGCGCACCCGTGGGGCCGCC

ATGCCGGCGATAATGGCCTGCTTCTCGCCGAAACGTTTGGTGGCGGGACCA

GTGACGAAGGCTTGAGCGAGGGCGTGCAAGATTCCGAATACCGCAAGCGA

CAGGCCGATCATCGTCGCGCTCCAGCGAAAGCGGTCCTCGCCGAAAATGA

CCCAGAGCGCTGCCGGCACCTGTCCTACGAGTTGCATGATAAAGAAGACA

GTCATAAGTGCGGCGACGATAGTCATGCCCCGCGCCCACCGGAAGGAGCT

GACTGGGTTGAAGGCTCTCAAGGGCATCGGTCGAGATCCCGGTGCCTAATG

AGTGAGCTAACTTACATTAATTGCGTTGCGCTCACTGCCCGCTTTCCAGTCG

GGAAACCTGTCGTGCCAGCTGCATTAATGAATCGGCCAACGCGCGGGGAG

AGGCGGTTTGCGTATTGGGCGCCAGGGTGGTTTTTCTTTTCACCAGTGAGA

CGGGCAACAGCTGATTGCCCTTCACCGCCTGGCCCTGAGAGAGTTGCAGC

AAGCGGTCCACGCTGGTTTGCCCCAGCAGGCGAAAATCCTGTTTGATGGTG

GTTAACGGCGGGATATAACATGAGCTGTCTTCGGTATCGTCGTATCCCACTA

CCGAGATATCCGCACCAACGCGCAGCCCGGACTCGGTAATGGCGCGCATTG

CGCCCAGCGCCATCTGATCGTTGGCAACCAGCATCGCAGTGGGAACGATGC

CCTCATTCAGCATTTGCATGGTTTGTTGAAAACCGGACATGGCACTCCAGT

CGCCTTCCCGTTCCGCTATCGGCTGAATTTGATTGCGAGTGAGATATTTATG

CCAGCCAGCCAGACGCAGACGCGCCGAGACAGAACTTAATGGGCCCGCTA

ACAGCGCGATTTGCTGGTGACCCAATGCGACCAGATGCTCCACGCCCAGTC

GCGTACCGTCTTCATGGGAGAAAATAATACTGTTGATGGGTGTCTGGTCAG

AGACATCAAGAAATAACGCCGGAACATTAGTGCAGGCAGCTTCCACAGCA

ATGGCATCCTGGTCATCCAGCGGATAGTTAATGATCAGCCCACTGACGCGTT

GCGCGAGAAGATTGTGCACCGCCGCTTTACAGGCTTCGACGCCGCTTCGTT

CTACCATCGACACCACCACGCTGGCACCCAGTTGATCGGCGCGAGATTTAA

TCGCCGCGACAATTTGCGACGGCGCGTGCAGGGCCAGACTGGAGGTGGCA

ACGCCAATCAGCAACGACTGTTTGCCCGCCAGTTGTTGTGCCACGCGGTTG

GGAATGTAATTCAGCTCCGCCATCGCCGCTTCCACTTTTTCCCGCGTTTTCG

CAGAAACGTGGCTGGCCTGGTTCACCACGCGGGAAACGGTCTGATAAGAG

ACACCGGCATACTCTGCGACATCGTATAACGTTACTGGTTTCACATTCACCA

CCCTGAATTGACTCTCTTCCGGGCGCTATCATGCCATACCGCGAAAGGTTTT

GCGCCATTCGATGGTGTCCGGGATCTCGACGCTCTCCCTTATGCGACTCCTG

CATTAGGAAGCAGCCCAGTAGTAGGTTGAGGCCGTTGAGCACCGCCGCCG

CAAGGAATGGTGCATGCAAGGAGATGGCGCCCAACAGTCCCCCGGCCACG

GGGCCTGCCACCATACCCACGCCGAAACAAGCGCTCATGAGCCCGAAGTG

GCGAGCCCGATCTTCCCCATCGGTGATGTCGGCGATATAGGCGCCAGCAAC

CGCACCTGTGGCGCCGGTGATGCCGGCCACGATGCGTCCGGCGTAGAGGA

TCGAGATCTCGATCCCGCGAAATTAATACGACTCACTATAGGGGAATTGTGA

GCGGATAACAATTCCCCTCTAGAAATAATTTTGTTTAACTTTAAGAAGGAGA

TATACCATGGGCAGCAGCCATCATCATCATCATCACAGCAGCGGCCTGGTGC

CGCGCGGCAGCCATATGGCTAGCATGACTGGTGGACAGCAAATGGGTCGC

GGATCCGAATTCGAGCTCCGTCGACAAGCTTGCATGGCTGAACAGATTCTG

AAAGTTAACGGTGTGGAAATTTGCGCAGAAAGTTTTGGCAAACCGACCGA

TCCGGCCATTCTGCTGATTATGGGCGCACAGATGAGCATGCTGTGGTGGGA

AGAAGAATTTTGTCAGCGTATTGCCGATGCCGGTCGCTTTGTTATTCGCTTT

GATAATCGTGATGTGGGTCGCAGCACCACCTATGAAGTTGGTCAGCCGGGT

TATACCTTTGAAGATATGGCAGATGATGCCGTTCATGTGCTGGATGCATTTG

GTGTGCAGCAGGCACATTTTGTTGGTATGAGTATGGGCGGTATGCTGACCC

AGATGATTGCCCTGCGTCATCCGGAACGCGTTCGTACAATTACCCTGCATGC

AACCAGCAATTTTGCACCGGGCCTGCCGCCTATTGATGAAAAACTGATGGA

ATTTTTCAGCAAGATGGGTGAAATCAACTGGGAAGATGAAAAAGAAGCAC

TGGAAGCAGCAGTTGCCAGCTGGAAAGTTCTGAGCGGTAGTAAACATCCG

TTTGATGAAAGCCGTGTGCGCGAACTGGCAAAAATTGATATTGCCCGTAGC

AATCATTACGCCAGCCGTAATAATCATGCATTTGTGACCGCAAGCGAACCGT

ATCTGCTGCGCACCGCTGAAATTGCCGTGCCTGCACTGGTTATTCATGGCAC

CGAAGATCTGCTGATTCCGTTTGCCCATGCACTGCATCTGGCCAATACCATT

CCGGGCGCAGTGCTGCTGACCTTAGAAGGTACCGGCCATGAACTGCCGTAT

GGCGATTGGGATGTGGTTATTGAAGCCATTCTGAAACATACCAGCGGTCGC

CGCGTTCGCCTGGCGGCCGCACTCGAGCACCACCACCACCACCACTGAGA

TCCGGCTGCTAACAAAGCCCGAAAGGAAGCTGAGTTGGCTGCTGCCACCG

CTGAGCAATAACTAGCATAACCCCTTGGGGCCTCTAAACGGGTCTTGAGGG

GTTTTTTGCTGAAAGGAGGAACTATATCCGGAT

pET-28a-PBB-1:

TGGCGAATGGGACGCGCCCTGTAGCGGCGCATTAAGCGCGGCGGGTGTGG

TGGTTACGCGCAGCGTGACCGCTACACTTGCCAGCGCCCTAGCGCCCGCTC

CTTTCGCTTTCTTCCCTTCCTTTCTCGCCACGTTCGCCGGCTTTCCCCGTCA

AGCTCTAAATCGGGGGCTCCCTTTAGGGTTCCGATTTAGTGCTTTACGGCAC

CTCGACCCCAAAAAACTTGATTAGGGTGATGGTTCACGTAGTGGGCCATCG

CCCTGATAGACGGTTTTTCGCCCTTTGACGTTGGAGTCCACGTTCTTTAATA

GTGGACTCTTGTTCCAAACTGGAACAACACTCAACCCTATCTCGGTCTATT

CTTTTGATTTATAAGGGATTTTGCCGATTTCGGCCTATTGGTTAAAAAATGA

GCTGATTTAACAAAAATTTAACGCGAATTTTAACAAAATATTAACGcTTACA

ATTTAGGTGGCACTTTTCGGGGAAATGTGCGCGGAACCCCTATTTGTTTATT

TTTCTAAATACATTCAAATATGTATCCGCTCATGAATTAATTCTTAGAAAAAC

TCATCGAGCATCAAATGAAACTGCAATTTATTCATATCAGGATTATCAATACC

ATATTTTTGAAAAAGCCGTTTCTGTAATGAAGGAGAAAACTCACCGAGGCA

GTTCCATAGGATGGCAAGATCCTGGTATCGGTCTGCGATTCCGACTCGTCCA

ACATCAATACAACCTATTAATTTCCCCTCGTCAAAAATAAGGTTATCAAGTG

AGAAATCACCATGAGTGACGACTGAATCCGGTGAGAATGGCAAAAGTTTAT

GCATTTCTTTCCAGACTTGTTCAACAGGCCAGCCATTACGCTCGTCATCAAA

ATCACTCGCATCAACCAAACCGTTATTCATTCGTGATTGCGCCTGAGCGAG

ACGAAATACGCGATCGCTGTTAAAAGGACAATTACAAACAGGAATCGAATG

CAACCGGCGCAGGAACACTGCCAGCGCATCAACAATATTTTCACCTGAATC

AGGATATTCTTCTAATACCTGGAATGCTGTTTTCCCGGGGATCGCAGTGGTG

AGTAACCATGCATCATCAGGAGTACGGATAAAATGCTTGATGGTCGGAAGA

GGCATAAATTCCGTCAGCCAGTTTAGTCTGACCATCTCATCTGTAACATCAT

TGGCAACGCTACCTTTGCCATGTTTCAGAAACAACTCTGGCGCATCGGGCT

TCCCATACAATCGATAGATTGTCGCACCTGATTGCCCGACATTATCGCGAGC

CCATTTATACCCATATAAATCAGCATCCATGTTGGAATTTAATCGCGGCCTAG

AGCAAGACGTTTCCCGTTGAATATGGCTCATAACACCCCTTGTATTACTGTT

TATGTAAGCAGACAGTTTTATTGTTCATGACCAAAATCCCTTAACGTGAGTT

TTCGTTCCACTGAGCGTCAGACCCCGTAGAAAAGATCAAAGGATCTTCTTG

AGATCCTTTTTTTCTGCGCGTAATCTGCTGCTTGCAAACAAAAAAACCACC

GCTACCAGCGGTGGTTTGTTTGCCGGATCAAGAGCTACCAACTCTTTTTCC

GAAGGTAACTGGCTTCAGCAGAGCGCAGATACCAAATACTGTCCTTCTAGT

GTAGCCGTAGTTAGGCCACCACTTCAAGAACTCTGTAGCACCGCCTACATA

CCTCGCTCTGCTAATCCTGTTACCAGTGGCTGCTGCCAGTGGCGATAAGTC

GTGTCTTACCGGGTTGGACTCAAGACGATAGTTACCGGATAAGGCGCAGCG

GTCGGGCTGAACGGGGGGTTCGTGCACACAGCCCAGCTTGGAGCGAACG

ACCTACACCGAACTGAGATACCTACAGCGTGAGCTATGAGAAAGCGCCAC

GCTTCCCGAAGGGAGAAAGGCGGACAGGTATCCGGTAAGCGGCAGGGTCG

GAACAGGAGAGCGCACGAGGGAGCTTCCAGGGGGAAACGCCTGGTATCTT

TATAGTCCTGTCGGGTTTCGCCACCTCTGACTTGAGCGTCGATTTTTGTGAT

GCTCGTCAGGGGGGCGGAGCCTATGGAAAAACGCCAGCAACGCGGCCTTT

TTACGGTTCCTGGCCTTTTGCTGGCCTTTTGCTCACATGTTCTTTCCTGCGTT

ATCCCCTGATTCTGTGGATAACCGTATTACCGCCTTTGAGTGAGCTGATACC

GCTCGCCGCAGCCGAACGACCGAGCGCAGCGAGTCAGTGAGCGAGGAAG

CGGAAGAGCGCCTGATGCGGTATTTTCTCCTTACGCATCTGTGCGGTATTTC

ACACCGCAATGGTGCACTCTCAGTACAATCTGCTCTGATGCCGCATAGTTAA

GCCAGTATACACTCCGCTATCGCTACGTGACTGGGTCATGGCTGCGCCCCG

ACACCCGCCAACACCCGCTGACGCGCCCTGACGGGCTTGTCTGCTCCCGG

CATCCGCTTACAGACAAGCTGTGACCGTCTCCGGGAGCTGCATGTGTCAGA

GGTTTTCACCGTCATCACCGAAACGCGCGAGGCAGCTGCGGTAAAGCTCAT

CAGCGTGGTCGTGAAGCGATTCACAGATGTCTGCCTGTTCATCCGCGTCCA

GCTCGTTGAGTTTCTCCAGAAGCGTTAATGTCTGGCTTCTGATAAAGCGGG

CCATGTTAAGGGCGGTTTTTTCCTGTTTGGTCACTGATGCCTCCGTGTAAGG

GGGATTTCTGTTCATGGGGGTAATGATACCGATGAAACGAGAGAGGATGCT

CACGATACGGGTTACTGATGATGAACATGCCCGGTTACTGGAACGTTGTGA

GGGTAAACAACTGGCGGTATGGATGCGGCGGGACCAGAGAAAAATCACTC

AGGGTCAATGCCAGCGCTTCGTTAATACAGATGTAGGTGTTCCACAGGGTA

GCCAGCAGCATCCTGCGATGCAGATCCGGAACATAATGGTGCAGGGCGCTG

ACTTCCGCGTTTCCAGACTTTACGAAACACGGAAACCGAAGACCATTCATG

TTGTTGCTCAGGTCGCAGACGTTTTGCAGCAGCAGTCGCTTCACGTTCGCT

CGCGTATCGGTGATTCATTCTGCTAACCAGTAAGGCAACCCCGCCAGCCTA

GCCGGGTCCTCAACGACAGGAGCACGATCATGCGCACCCGTGGGGCCGCC

ATGCCGGCGATAATGGCCTGCTTCTCGCCGAAACGTTTGGTGGCGGGACCA

GTGACGAAGGCTTGAGCGAGGGCGTGCAAGATTCCGAATACCGCAAGCGA

CAGGCCGATCATCGTCGCGCTCCAGCGAAAGCGGTCCTCGCCGAAAATGA

CCCAGAGCGCTGCCGGCACCTGTCCTACGAGTTGCATGATAAAGAAGACA

GTCATAAGTGCGGCGACGATAGTCATGCCCCGCGCCCACCGGAAGGAGCT

GACTGGGTTGAAGGCTCTCAAGGGCATCGGTCGAGATCCCGGTGCCTAATG

AGTGAGCTAACTTACATTAATTGCGTTGCGCTCACTGCCCGCTTTCCAGTCG

GGAAACCTGTCGTGCCAGCTGCATTAATGAATCGGCCAACGCGCGGGGAG

AGGCGGTTTGCGTATTGGGCGCCAGGGTGGTTTTTCTTTTCACCAGTGAGA

CGGGCAACAGCTGATTGCCCTTCACCGCCTGGCCCTGAGAGAGTTGCAGC

AAGCGGTCCACGCTGGTTTGCCCCAGCAGGCGAAAATCCTGTTTGATGGTG

GTTAACGGCGGGATATAACATGAGCTGTCTTCGGTATCGTCGTATCCCACTA

CCGAGATATCCGCACCAACGCGCAGCCCGGACTCGGTAATGGCGCGCATTG

CGCCCAGCGCCATCTGATCGTTGGCAACCAGCATCGCAGTGGGAACGATGC

CCTCATTCAGCATTTGCATGGTTTGTTGAAAACCGGACATGGCACTCCAGT

CGCCTTCCCGTTCCGCTATCGGCTGAATTTGATTGCGAGTGAGATATTTATG

CCAGCCAGCCAGACGCAGACGCGCCGAGACAGAACTTAATGGGCCCGCTA

ACAGCGCGATTTGCTGGTGACCCAATGCGACCAGATGCTCCACGCCCAGTC

GCGTACCGTCTTCATGGGAGAAAATAATACTGTTGATGGGTGTCTGGTCAG

AGACATCAAGAAATAACGCCGGAACATTAGTGCAGGCAGCTTCCACAGCA

ATGGCATCCTGGTCATCCAGCGGATAGTTAATGATCAGCCCACTGACGCGTT

GCGCGAGAAGATTGTGCACCGCCGCTTTACAGGCTTCGACGCCGCTTCGTT

CTACCATCGACACCACCACGCTGGCACCCAGTTGATCGGCGCGAGATTTAA

TCGCCGCGACAATTTGCGACGGCGCGTGCAGGGCCAGACTGGAGGTGGCA

ACGCCAATCAGCAACGACTGTTTGCCCGCCAGTTGTTGTGCCACGCGGTTG

GGAATGTAATTCAGCTCCGCCATCGCCGCTTCCACTTTTTCCCGCGTTTTCG

CAGAAACGTGGCTGGCCTGGTTCACCACGCGGGAAACGGTCTGATAAGAG

ACACCGGCATACTCTGCGACATCGTATAACGTTACTGGTTTCACATTCACCA

CCCTGAATTGACTCTCTTCCGGGCGCTATCATGCCATACCGCGAAAGGTTTT

GCGCCATTCGATGGTGTCCGGGATCTCGACGCTCTCCCTTATGCGACTCCTG

CATTAGGAAGCAGCCCAGTAGTAGGTTGAGGCCGTTGAGCACCGCCGCCG

CAAGGAATGGTGCATGCAAGGAGATGGCGCCCAACAGTCCCCCGGCCACG

GGGCCTGCCACCATACCCACGCCGAAACAAGCGCTCATGAGCCCGAAGTG

GCGAGCCCGATCTTCCCCATCGGTGATGTCGGCGATATAGGCGCCAGCAAC

CGCACCTGTGGCGCCGGTGATGCCGGCCACGATGCGTCCGGCGTAGAGGA

TCGAGATCTCGATCCCGCGAAATTAATACGACTCACTATAGGGGAATTGTGA

GCGGATAACAATTCCCCTCTAGAAATAATTTTGTTTAACTTTAAGAAGGAGA

TATACCATGGGCAGCAGCCATCATCATCATCATCACAGCAGCGGCCTGGTGC

CGCGCGGCAGCCATATGGCTAGCATGACTGGTGGACAGCAAATGGGTCGC

GGATCCGAATTCGAGCTCCGTCGACAAGCTTGCATGAACGAGCGTCTGATT

AAGATTGATGGTATTGAAATCTGCACCGAAAGTTTTGGCAAACAGGTTAAT

CCGGCCATTCTGCTGATTATGGGCGCCCAGAGCAGCATGATTTGGTGGGAA

GAAGAATTTTGTCGTCGCCTGGCCGATGCAGGTCGCTATGTTATTCGCTATG

ATAATCGTGATGTGGGCCGCAGCACCACCTATGAACTGGGTCAGCCGGGTT

ATACCTTTGAAGATATGGCCGATGATGCCATTCGTGTGCTGGATGCCTATGA

AATTGAACAGGCACATATTGTTGGCATGAGTATGGGTGGCATGCTGACCCA

GATTATTGCACTGCGTCATCCGGGTCGTGTGCGCACCATTACCCTGTTAAGC

ACCAGCAATTTTGCACCGGATCTGCCGCTGATGGAAGAACGTATTATGGATT

ATTTCAGCAACGTGGGCGCAATTGATTGGACCAATGAACAGGCAGTGGTG

GAATTTGCAATTGGTCGCAGTCGTATTCTGGTTGGTAGCAAACATAGCTTTG

ATGAAAAGCGCATCTATAACCTGGCAAAAGAAGAAGTTAAGCGTAGTCATA

ACATGGCCAGTATGAATAATCACGGTATGCTGGTGGGCGGTGAAAGTTATCT

GGTGCGTACCGGTGAAATTAAGGTGCCGGCACTGGTTATTCATGGTACCGA

AGATCCGATTATTCCGTATGAACATGGCAAAAATCTGGTTAATGAGATCAGC

GCCGCCGTGCTGCTGACCTTAGAAGGTACCGGTCATGAACTGCATTATGAT

GATTGGGATCTGATTATCGACAGTATTAGCAATCACACCAGTATTCCGAATG

AAAATGAGTTTAGCAACGCGGCCGCACTCGAGCACCACCACCACCACCAC

TGAGATCCGGCTGCTAACAAAGCCCGAAAGGAAGCTGAGTTGGCTGCTGC

CACCGCTGAGCAATAACTAGCATAACCCCTTGGGGCCTCTAAACGGGTCTT

GAGGGGTTTTTTGCTGAAAGGAGGAACTATATCCGGAT

pET-28a-CBF-1:

TGGCGAATGGGACGCGCCCTGTAGCGGCGCATTAAGCGCGGCGGGTGTGG

TGGTTACGCGCAGCGTGACCGCTACACTTGCCAGCGCCCTAGCGCCCGCTC

CTTTCGCTTTCTTCCCTTCCTTTCTCGCCACGTTCGCCGGCTTTCCCCGTCA

AGCTCTAAATCGGGGGCTCCCTTTAGGGTTCCGATTTAGTGCTTTACGGCAC

CTCGACCCCAAAAAACTTGATTAGGGTGATGGTTCACGTAGTGGGCCATCG

CCCTGATAGACGGTTTTTCGCCCTTTGACGTTGGAGTCCACGTTCTTTAATA

GTGGACTCTTGTTCCAAACTGGAACAACACTCAACCCTATCTCGGTCTATT

CTTTTGATTTATAAGGGATTTTGCCGATTTCGGCCTATTGGTTAAAAAATGA

GCTGATTTAACAAAAATTTAACGCGAATTTTAACAAAATATTAACGcTTACA

ATTTAGGTGGCACTTTTCGGGGAAATGTGCGCGGAACCCCTATTTGTTTATT

TTTCTAAATACATTCAAATATGTATCCGCTCATGAATTAATTCTTAGAAAAAC

TCATCGAGCATCAAATGAAACTGCAATTTATTCATATCAGGATTATCAATACC

ATATTTTTGAAAAAGCCGTTTCTGTAATGAAGGAGAAAACTCACCGAGGCA

GTTCCATAGGATGGCAAGATCCTGGTATCGGTCTGCGATTCCGACTCGTCCA

ACATCAATACAACCTATTAATTTCCCCTCGTCAAAAATAAGGTTATCAAGTG

AGAAATCACCATGAGTGACGACTGAATCCGGTGAGAATGGCAAAAGTTTAT

GCATTTCTTTCCAGACTTGTTCAACAGGCCAGCCATTACGCTCGTCATCAAA

ATCACTCGCATCAACCAAACCGTTATTCATTCGTGATTGCGCCTGAGCGAG

ACGAAATACGCGATCGCTGTTAAAAGGACAATTACAAACAGGAATCGAATG

CAACCGGCGCAGGAACACTGCCAGCGCATCAACAATATTTTCACCTGAATC

AGGATATTCTTCTAATACCTGGAATGCTGTTTTCCCGGGGATCGCAGTGGTG

AGTAACCATGCATCATCAGGAGTACGGATAAAATGCTTGATGGTCGGAAGA

GGCATAAATTCCGTCAGCCAGTTTAGTCTGACCATCTCATCTGTAACATCAT

TGGCAACGCTACCTTTGCCATGTTTCAGAAACAACTCTGGCGCATCGGGCT

TCCCATACAATCGATAGATTGTCGCACCTGATTGCCCGACATTATCGCGAGC

CCATTTATACCCATATAAATCAGCATCCATGTTGGAATTTAATCGCGGCCTAG

AGCAAGACGTTTCCCGTTGAATATGGCTCATAACACCCCTTGTATTACTGTT

TATGTAAGCAGACAGTTTTATTGTTCATGACCAAAATCCCTTAACGTGAGTT

TTCGTTCCACTGAGCGTCAGACCCCGTAGAAAAGATCAAAGGATCTTCTTG

AGATCCTTTTTTTCTGCGCGTAATCTGCTGCTTGCAAACAAAAAAACCACC

GCTACCAGCGGTGGTTTGTTTGCCGGATCAAGAGCTACCAACTCTTTTTCC

GAAGGTAACTGGCTTCAGCAGAGCGCAGATACCAAATACTGTCCTTCTAGT

GTAGCCGTAGTTAGGCCACCACTTCAAGAACTCTGTAGCACCGCCTACATA

CCTCGCTCTGCTAATCCTGTTACCAGTGGCTGCTGCCAGTGGCGATAAGTC

GTGTCTTACCGGGTTGGACTCAAGACGATAGTTACCGGATAAGGCGCAGCG

GTCGGGCTGAACGGGGGGTTCGTGCACACAGCCCAGCTTGGAGCGAACG

ACCTACACCGAACTGAGATACCTACAGCGTGAGCTATGAGAAAGCGCCAC

GCTTCCCGAAGGGAGAAAGGCGGACAGGTATCCGGTAAGCGGCAGGGTCG

GAACAGGAGAGCGCACGAGGGAGCTTCCAGGGGGAAACGCCTGGTATCTT

TATAGTCCTGTCGGGTTTCGCCACCTCTGACTTGAGCGTCGATTTTTGTGAT

GCTCGTCAGGGGGGCGGAGCCTATGGAAAAACGCCAGCAACGCGGCCTTT

TTACGGTTCCTGGCCTTTTGCTGGCCTTTTGCTCACATGTTCTTTCCTGCGTT

ATCCCCTGATTCTGTGGATAACCGTATTACCGCCTTTGAGTGAGCTGATACC

GCTCGCCGCAGCCGAACGACCGAGCGCAGCGAGTCAGTGAGCGAGGAAG

CGGAAGAGCGCCTGATGCGGTATTTTCTCCTTACGCATCTGTGCGGTATTTC

ACACCGCAATGGTGCACTCTCAGTACAATCTGCTCTGATGCCGCATAGTTAA

GCCAGTATACACTCCGCTATCGCTACGTGACTGGGTCATGGCTGCGCCCCG

ACACCCGCCAACACCCGCTGACGCGCCCTGACGGGCTTGTCTGCTCCCGG

CATCCGCTTACAGACAAGCTGTGACCGTCTCCGGGAGCTGCATGTGTCAGA

GGTTTTCACCGTCATCACCGAAACGCGCGAGGCAGCTGCGGTAAAGCTCAT

CAGCGTGGTCGTGAAGCGATTCACAGATGTCTGCCTGTTCATCCGCGTCCA

GCTCGTTGAGTTTCTCCAGAAGCGTTAATGTCTGGCTTCTGATAAAGCGGG

CCATGTTAAGGGCGGTTTTTTCCTGTTTGGTCACTGATGCCTCCGTGTAAGG

GGGATTTCTGTTCATGGGGGTAATGATACCGATGAAACGAGAGAGGATGCT

CACGATACGGGTTACTGATGATGAACATGCCCGGTTACTGGAACGTTGTGA

GGGTAAACAACTGGCGGTATGGATGCGGCGGGACCAGAGAAAAATCACTC

AGGGTCAATGCCAGCGCTTCGTTAATACAGATGTAGGTGTTCCACAGGGTA

GCCAGCAGCATCCTGCGATGCAGATCCGGAACATAATGGTGCAGGGCGCTG

ACTTCCGCGTTTCCAGACTTTACGAAACACGGAAACCGAAGACCATTCATG

TTGTTGCTCAGGTCGCAGACGTTTTGCAGCAGCAGTCGCTTCACGTTCGCT

CGCGTATCGGTGATTCATTCTGCTAACCAGTAAGGCAACCCCGCCAGCCTA

GCCGGGTCCTCAACGACAGGAGCACGATCATGCGCACCCGTGGGGCCGCC

ATGCCGGCGATAATGGCCTGCTTCTCGCCGAAACGTTTGGTGGCGGGACCA

GTGACGAAGGCTTGAGCGAGGGCGTGCAAGATTCCGAATACCGCAAGCGA

CAGGCCGATCATCGTCGCGCTCCAGCGAAAGCGGTCCTCGCCGAAAATGA

CCCAGAGCGCTGCCGGCACCTGTCCTACGAGTTGCATGATAAAGAAGACA

GTCATAAGTGCGGCGACGATAGTCATGCCCCGCGCCCACCGGAAGGAGCT

GACTGGGTTGAAGGCTCTCAAGGGCATCGGTCGAGATCCCGGTGCCTAATG

AGTGAGCTAACTTACATTAATTGCGTTGCGCTCACTGCCCGCTTTCCAGTCG

GGAAACCTGTCGTGCCAGCTGCATTAATGAATCGGCCAACGCGCGGGGAG

AGGCGGTTTGCGTATTGGGCGCCAGGGTGGTTTTTCTTTTCACCAGTGAGA

CGGGCAACAGCTGATTGCCCTTCACCGCCTGGCCCTGAGAGAGTTGCAGC

AAGCGGTCCACGCTGGTTTGCCCCAGCAGGCGAAAATCCTGTTTGATGGTG

GTTAACGGCGGGATATAACATGAGCTGTCTTCGGTATCGTCGTATCCCACTA

CCGAGATATCCGCACCAACGCGCAGCCCGGACTCGGTAATGGCGCGCATTG

CGCCCAGCGCCATCTGATCGTTGGCAACCAGCATCGCAGTGGGAACGATGC

CCTCATTCAGCATTTGCATGGTTTGTTGAAAACCGGACATGGCACTCCAGT

CGCCTTCCCGTTCCGCTATCGGCTGAATTTGATTGCGAGTGAGATATTTATG

CCAGCCAGCCAGACGCAGACGCGCCGAGACAGAACTTAATGGGCCCGCTA

ACAGCGCGATTTGCTGGTGACCCAATGCGACCAGATGCTCCACGCCCAGTC

GCGTACCGTCTTCATGGGAGAAAATAATACTGTTGATGGGTGTCTGGTCAG

AGACATCAAGAAATAACGCCGGAACATTAGTGCAGGCAGCTTCCACAGCA

ATGGCATCCTGGTCATCCAGCGGATAGTTAATGATCAGCCCACTGACGCGTT

GCGCGAGAAGATTGTGCACCGCCGCTTTACAGGCTTCGACGCCGCTTCGTT

CTACCATCGACACCACCACGCTGGCACCCAGTTGATCGGCGCGAGATTTAA

TCGCCGCGACAATTTGCGACGGCGCGTGCAGGGCCAGACTGGAGGTGGCA

ACGCCAATCAGCAACGACTGTTTGCCCGCCAGTTGTTGTGCCACGCGGTTG

GGAATGTAATTCAGCTCCGCCATCGCCGCTTCCACTTTTTCCCGCGTTTTCG

CAGAAACGTGGCTGGCCTGGTTCACCACGCGGGAAACGGTCTGATAAGAG

ACACCGGCATACTCTGCGACATCGTATAACGTTACTGGTTTCACATTCACCA

CCCTGAATTGACTCTCTTCCGGGCGCTATCATGCCATACCGCGAAAGGTTTT

GCGCCATTCGATGGTGTCCGGGATCTCGACGCTCTCCCTTATGCGACTCCTG

CATTAGGAAGCAGCCCAGTAGTAGGTTGAGGCCGTTGAGCACCGCCGCCG

CAAGGAATGGTGCATGCAAGGAGATGGCGCCCAACAGTCCCCCGGCCACG

GGGCCTGCCACCATACCCACGCCGAAACAAGCGCTCATGAGCCCGAAGTG

GCGAGCCCGATCTTCCCCATCGGTGATGTCGGCGATATAGGCGCCAGCAAC

CGCACCTGTGGCGCCGGTGATGCCGGCCACGATGCGTCCGGCGTAGAGGA

TCGAGATCTCGATCCCGCGAAATTAATACGACTCACTATAGGGGAATTGTGA

GCGGATAACAATTCCCCTCTAGAAATAATTTTGTTTAACTTTAAGAAGGAGA

TATACCATGGGCAGCAGCCATCATCATCATCATCACAGCAGCGGCCTGGTGC

CGCGCGGCAGCCATATGGCTAGCATGACTGGTGGACAGCAAATGGGTCGC

GGATCCGAATTCGAGCTCCGTCGACAAGCTTGCATGCGTGAACAGATTATG

AAGATCAACAAGGTTAACATCTGCACCGAAAGTTTTGGCAATAGTGAAGAT

CCGGCAATTCTGCTGATTATGGGTGCAATGACCAGCCTGGATTGGTGGGAT

GAAGATTTTTGCCTGCGTCTGGCCGATCAGGGTCGCTTTGTGATTCGTTATG

ATCATCGCGATCTGGGCCGTAGCACCACCTATGAACCGGGTACCAGCAATTA

TACCATTACCGATCTGGCCGATGATGCCGCAGGCGTTCTGGATGCATATCAT

ATTGGTCAGGCACATATTGTGGGTATGAGCATGGGCGGTCTGACCGGTCAG

ATTCTGGCCTTACGCTATCCGGATCGTGTTCTGACCCTGACCCTGATTGCCA

GCAGTGTTTTTGGCACCGAAATGGAAAAACTGCCGCCGATGGATCAGAATA

TTCTGGATTATCATGCGAAGAGTGCCAGCATTGATTGGACCAATCGCGATGC

CGCCATTCCGTATCTGGCAGGTGGTTGGAAAACCCTGGCAGGTAGCAAACC

GTTTGAACAGGAACGTATCTATAAGCTGGCCGAACGTGAAGCCGATCGCGC

AAATCATCTGCCGAGCCGTTTTAATCATGCACTGCTGCAGGGTGGTGACGT

GTATTTTGATCGTATGAATGAAATCAGCGCGCCGGTTCTGATTATTCATGGC

ACCGAAGATCCGGCGCTGCCGTATGAACATGGTCTGGCACTGAAAAAAGC

AATTCCGCATAGTGAACTGGTTACCCTGGAAGGCACCGGCCATGAAATTCA

TAGTGAAGATTGGAATCAGATCATCGATAGTGTTGTTAAGCTGAGTAGCCGT

CTGGAAGAAGCGGCCGCACTCGAGCACCACCACCACCACCACTGAGATCC

GGCTGCTAACAAAGCCCGAAAGGAAGCTGAGTTGGCTGCTGCCACCGCTG

AGCAATAACTAGCATAACCCCTTGGGGCCTCTAAACGGGTCTTGAGGGGTT

TTTTGCTGAAAGGAGGAACTATATCCGGAT

pET-28a-PBA-1:

TGGCGAATGGGACGCGCCCTGTAGCGGCGCATTAAGCGCGGCGGGTGTGG

TGGTTACGCGCAGCGTGACCGCTACACTTGCCAGCGCCCTAGCGCCCGCTC

CTTTCGCTTTCTTCCCTTCCTTTCTCGCCACGTTCGCCGGCTTTCCCCGTCA

AGCTCTAAATCGGGGGCTCCCTTTAGGGTTCCGATTTAGTGCTTTACGGCAC

CTCGACCCCAAAAAACTTGATTAGGGTGATGGTTCACGTAGTGGGCCATCG

CCCTGATAGACGGTTTTTCGCCCTTTGACGTTGGAGTCCACGTTCTTTAATA

GTGGACTCTTGTTCCAAACTGGAACAACACTCAACCCTATCTCGGTCTATT

CTTTTGATTTATAAGGGATTTTGCCGATTTCGGCCTATTGGTTAAAAAATGA

GCTGATTTAACAAAAATTTAACGCGAATTTTAACAAAATATTAACGcTTACA

ATTTAGGTGGCACTTTTCGGGGAAATGTGCGCGGAACCCCTATTTGTTTATT

TTTCTAAATACATTCAAATATGTATCCGCTCATGAATTAATTCTTAGAAAAAC

TCATCGAGCATCAAATGAAACTGCAATTTATTCATATCAGGATTATCAATACC

ATATTTTTGAAAAAGCCGTTTCTGTAATGAAGGAGAAAACTCACCGAGGCA

GTTCCATAGGATGGCAAGATCCTGGTATCGGTCTGCGATTCCGACTCGTCCA

ACATCAATACAACCTATTAATTTCCCCTCGTCAAAAATAAGGTTATCAAGTG

AGAAATCACCATGAGTGACGACTGAATCCGGTGAGAATGGCAAAAGTTTAT

GCATTTCTTTCCAGACTTGTTCAACAGGCCAGCCATTACGCTCGTCATCAAA

ATCACTCGCATCAACCAAACCGTTATTCATTCGTGATTGCGCCTGAGCGAG

ACGAAATACGCGATCGCTGTTAAAAGGACAATTACAAACAGGAATCGAATG

CAACCGGCGCAGGAACACTGCCAGCGCATCAACAATATTTTCACCTGAATC

AGGATATTCTTCTAATACCTGGAATGCTGTTTTCCCGGGGATCGCAGTGGTG

AGTAACCATGCATCATCAGGAGTACGGATAAAATGCTTGATGGTCGGAAGA

GGCATAAATTCCGTCAGCCAGTTTAGTCTGACCATCTCATCTGTAACATCAT

TGGCAACGCTACCTTTGCCATGTTTCAGAAACAACTCTGGCGCATCGGGCT

TCCCATACAATCGATAGATTGTCGCACCTGATTGCCCGACATTATCGCGAGC

CCATTTATACCCATATAAATCAGCATCCATGTTGGAATTTAATCGCGGCCTAG

AGCAAGACGTTTCCCGTTGAATATGGCTCATAACACCCCTTGTATTACTGTT

TATGTAAGCAGACAGTTTTATTGTTCATGACCAAAATCCCTTAACGTGAGTT

TTCGTTCCACTGAGCGTCAGACCCCGTAGAAAAGATCAAAGGATCTTCTTG

AGATCCTTTTTTTCTGCGCGTAATCTGCTGCTTGCAAACAAAAAAACCACC

GCTACCAGCGGTGGTTTGTTTGCCGGATCAAGAGCTACCAACTCTTTTTCC

GAAGGTAACTGGCTTCAGCAGAGCGCAGATACCAAATACTGTCCTTCTAGT

GTAGCCGTAGTTAGGCCACCACTTCAAGAACTCTGTAGCACCGCCTACATA

CCTCGCTCTGCTAATCCTGTTACCAGTGGCTGCTGCCAGTGGCGATAAGTC

GTGTCTTACCGGGTTGGACTCAAGACGATAGTTACCGGATAAGGCGCAGCG

GTCGGGCTGAACGGGGGGTTCGTGCACACAGCCCAGCTTGGAGCGAACG

ACCTACACCGAACTGAGATACCTACAGCGTGAGCTATGAGAAAGCGCCAC

GCTTCCCGAAGGGAGAAAGGCGGACAGGTATCCGGTAAGCGGCAGGGTCG

GAACAGGAGAGCGCACGAGGGAGCTTCCAGGGGGAAACGCCTGGTATCTT

TATAGTCCTGTCGGGTTTCGCCACCTCTGACTTGAGCGTCGATTTTTGTGAT

GCTCGTCAGGGGGGCGGAGCCTATGGAAAAACGCCAGCAACGCGGCCTTT

TTACGGTTCCTGGCCTTTTGCTGGCCTTTTGCTCACATGTTCTTTCCTGCGTT

ATCCCCTGATTCTGTGGATAACCGTATTACCGCCTTTGAGTGAGCTGATACC

GCTCGCCGCAGCCGAACGACCGAGCGCAGCGAGTCAGTGAGCGAGGAAG

CGGAAGAGCGCCTGATGCGGTATTTTCTCCTTACGCATCTGTGCGGTATTTC

ACACCGCAATGGTGCACTCTCAGTACAATCTGCTCTGATGCCGCATAGTTAA

GCCAGTATACACTCCGCTATCGCTACGTGACTGGGTCATGGCTGCGCCCCG

ACACCCGCCAACACCCGCTGACGCGCCCTGACGGGCTTGTCTGCTCCCGG

CATCCGCTTACAGACAAGCTGTGACCGTCTCCGGGAGCTGCATGTGTCAGA

GGTTTTCACCGTCATCACCGAAACGCGCGAGGCAGCTGCGGTAAAGCTCAT

CAGCGTGGTCGTGAAGCGATTCACAGATGTCTGCCTGTTCATCCGCGTCCA

GCTCGTTGAGTTTCTCCAGAAGCGTTAATGTCTGGCTTCTGATAAAGCGGG

CCATGTTAAGGGCGGTTTTTTCCTGTTTGGTCACTGATGCCTCCGTGTAAGG

GGGATTTCTGTTCATGGGGGTAATGATACCGATGAAACGAGAGAGGATGCT

CACGATACGGGTTACTGATGATGAACATGCCCGGTTACTGGAACGTTGTGA

GGGTAAACAACTGGCGGTATGGATGCGGCGGGACCAGAGAAAAATCACTC

AGGGTCAATGCCAGCGCTTCGTTAATACAGATGTAGGTGTTCCACAGGGTA

GCCAGCAGCATCCTGCGATGCAGATCCGGAACATAATGGTGCAGGGCGCTG

ACTTCCGCGTTTCCAGACTTTACGAAACACGGAAACCGAAGACCATTCATG

TTGTTGCTCAGGTCGCAGACGTTTTGCAGCAGCAGTCGCTTCACGTTCGCT

CGCGTATCGGTGATTCATTCTGCTAACCAGTAAGGCAACCCCGCCAGCCTA

GCCGGGTCCTCAACGACAGGAGCACGATCATGCGCACCCGTGGGGCCGCC

ATGCCGGCGATAATGGCCTGCTTCTCGCCGAAACGTTTGGTGGCGGGACCA

GTGACGAAGGCTTGAGCGAGGGCGTGCAAGATTCCGAATACCGCAAGCGA

CAGGCCGATCATCGTCGCGCTCCAGCGAAAGCGGTCCTCGCCGAAAATGA

CCCAGAGCGCTGCCGGCACCTGTCCTACGAGTTGCATGATAAAGAAGACA

GTCATAAGTGCGGCGACGATAGTCATGCCCCGCGCCCACCGGAAGGAGCT

GACTGGGTTGAAGGCTCTCAAGGGCATCGGTCGAGATCCCGGTGCCTAATG

AGTGAGCTAACTTACATTAATTGCGTTGCGCTCACTGCCCGCTTTCCAGTCG

GGAAACCTGTCGTGCCAGCTGCATTAATGAATCGGCCAACGCGCGGGGAG

AGGCGGTTTGCGTATTGGGCGCCAGGGTGGTTTTTCTTTTCACCAGTGAGA

CGGGCAACAGCTGATTGCCCTTCACCGCCTGGCCCTGAGAGAGTTGCAGC

AAGCGGTCCACGCTGGTTTGCCCCAGCAGGCGAAAATCCTGTTTGATGGTG

GTTAACGGCGGGATATAACATGAGCTGTCTTCGGTATCGTCGTATCCCACTA

CCGAGATATCCGCACCAACGCGCAGCCCGGACTCGGTAATGGCGCGCATTG

CGCCCAGCGCCATCTGATCGTTGGCAACCAGCATCGCAGTGGGAACGATGC

CCTCATTCAGCATTTGCATGGTTTGTTGAAAACCGGACATGGCACTCCAGT

CGCCTTCCCGTTCCGCTATCGGCTGAATTTGATTGCGAGTGAGATATTTATG

CCAGCCAGCCAGACGCAGACGCGCCGAGACAGAACTTAATGGGCCCGCTA

ACAGCGCGATTTGCTGGTGACCCAATGCGACCAGATGCTCCACGCCCAGTC

GCGTACCGTCTTCATGGGAGAAAATAATACTGTTGATGGGTGTCTGGTCAG

AGACATCAAGAAATAACGCCGGAACATTAGTGCAGGCAGCTTCCACAGCA

ATGGCATCCTGGTCATCCAGCGGATAGTTAATGATCAGCCCACTGACGCGTT

GCGCGAGAAGATTGTGCACCGCCGCTTTACAGGCTTCGACGCCGCTTCGTT

CTACCATCGACACCACCACGCTGGCACCCAGTTGATCGGCGCGAGATTTAA

TCGCCGCGACAATTTGCGACGGCGCGTGCAGGGCCAGACTGGAGGTGGCA

ACGCCAATCAGCAACGACTGTTTGCCCGCCAGTTGTTGTGCCACGCGGTTG

GGAATGTAATTCAGCTCCGCCATCGCCGCTTCCACTTTTTCCCGCGTTTTCG

CAGAAACGTGGCTGGCCTGGTTCACCACGCGGGAAACGGTCTGATAAGAG

ACACCGGCATACTCTGCGACATCGTATAACGTTACTGGTTTCACATTCACCA

CCCTGAATTGACTCTCTTCCGGGCGCTATCATGCCATACCGCGAAAGGTTTT

GCGCCATTCGATGGTGTCCGGGATCTCGACGCTCTCCCTTATGCGACTCCTG

CATTAGGAAGCAGCCCAGTAGTAGGTTGAGGCCGTTGAGCACCGCCGCCG

CAAGGAATGGTGCATGCAAGGAGATGGCGCCCAACAGTCCCCCGGCCACG

GGGCCTGCCACCATACCCACGCCGAAACAAGCGCTCATGAGCCCGAAGTG

GCGAGCCCGATCTTCCCCATCGGTGATGTCGGCGATATAGGCGCCAGCAAC

CGCACCTGTGGCGCCGGTGATGCCGGCCACGATGCGTCCGGCGTAGAGGA

TCGAGATCTCGATCCCGCGAAATTAATACGACTCACTATAGGGGAATTGTGA

GCGGATAACAATTCCCCTCTAGAAATAATTTTGTTTAACTTTAAGAAGGAGA

TATACCATGGGCAGCAGCCATCATCATCATCATCACAGCAGCGGCCTGGTGC

CGCGCGGCAGCCATATGGCTAGCATGACTGGTGGACAGCAAATGGGTCGC

GGATCCGAATTCGAGCTCCGTCGACAAGCTTGCATGAGCGAAAAGCTGATT

AAGATCAACGGCATTGATAACGGCATTGACATTTGCACCGAAAGTTTTGGT

AACCCGAATAATCCGGCAATTCTGCTGATTATGGGTGCCATGTGCAGTATGG

TTTATTGGGATGAAGAATTCTGCCAGCGTCTGGCCGATACCGGCAGATATGT

TATTCGTTATGATAACCGTGACGTGGGTCGCAGTATTGCCTATGAACCGGGT

AATAGTCAGTATACCGTTGAAGATATGGCCGATGATGCCATTGGTGTGCTGG

ATGCATATAGTATTGATGAAGCACATATCGTGGGTATGAGCCTGGGTGGTAT

GATTGCACAGATTATTGCCCTGCGCCATCCGCAGCGTGTTCTGACCATTACC

ATGATTGCCAGTAGTATTTTCGGCAGTGATGATAATAACCGCGATCTGCCGC

CGATGGATGAAAATATTCTGGCATATCATGCCAACGGTGCCACCGTTAATTG

GAGCGATGAAGAAAGTGTGGCCAATTATCTGGTTGCCGGCAGTGGCCTGCT

GTGCGGTAGCAAGCATAAATTTGATGAAAAGCGTGTGTACAAGCAGGTGC

GTAAAGAAATTAAGCGCGCAAATAATCTGCTGAGCATGTTTAATCACGCCAT

TCTGAAAGGCGATGCCAGCTATGAAGGTAAAATTAAGCGTATTAAGGTGCC

GGCCCTGGTTATTCATGGTACCGAAGATACCGTTCTGCCGTATGAACATGGT

CTGGCACTGGCAAATGAAATTCCGAATGCAAGTCTGCTGACCCTGGATGGT

ACCGGCCATGAAATTCATTTTGATGATTGGGATAACATCATCAACGCAATTA

GCAATCACACCAGTGTTGTTGCGGCCGCACTCGAGCACCACCACCACCAC

CACTGAGATCCGGCTGCTAACAAAGCCCGAAAGGAAGCTGAGTTGGCTGC

TGCCACCGCTGAGCAATAACTAGCATAACCCCTTGGGGCCTCTAAACGGGT

CTTGAGGGGTTTTTTGCTGAAAGGAGGAACTATATCCGGAT

Supplementary material 21

The plasmid sequence from *B. subtilis* harboring the MLE gene:

pHT43-BCA-1:

TTAAGTTATTGGTATGACTGGTTTTAAGCGCAAAAAAAGTTGCTTTTTCGTA

CCTATTAATGTATCGTTTTAGAAAACCGACTGTAAAAAGTACAGTCGGCATT

ATCTCATATTATAAAAGCCAGTCATTAGGCCTATCTGACAATTCCTGAATAGA

GTTCATAAACAATCCTGCATGATAACCATCACAAACAGAATGATGTACCTGT

AAAGATAGCGGTAAATATATTGAATTACCTTTATTAATGAATTTTCCTGCTGT

AATAATGGGTAGAAGGTAATTACTATTATTATTGATATTTAAGTTAAACCCAG

TAAATGAAGTCCATGGAATAATAGAAAGAGAAAAAGCATTTTCAGGTATAG

GTGTTTTGGGAAACAATTTCCCCGAACCATTATATTTCTCTACATCAGAAAG

GTATAAATCATAAAACTCTTTGAAGTCATTCTTTACAGGAGTCCAAATACCA

GAGAATGTTTTAGATACACCATCAAAAATTGTATAAAGTGGCTCTAACTTAT

CCCAATAACCTAACTCTCCGTCGCTATTGTAACCAGTTCTAAAAGCTGTATT

TGAGTTTATCACCCTTGTCACTAAGAAAATAAATGCAGGGTAAAATTTATAT

CCTTCTTGTTTTATGTTTCGGTATAAAACACTAATATCAATTTCTGTGGTTATA

CTAAAAGTCGTTTGTTGGTTCAAATAATGATTAAATATCTCTTTTCTCTTCCA

ATTGTCTAAATCAATTTTATTAAAGTTCATTTGATATGCCTCCTAAATTTTTAT

CTAAAGTGAATTTAGGAGGCTTACTTGTCTGCTTTCTTCATTAGAATCAATC

CTTTTTTAAAAGTCAATATTACTGTAACATAAATATATATTTTAAAAATATCCC

ACTTTATCCAATTTTCGTTTGTTGAACTAATGGGTGCTTTAGTTGAAGAATA

AAGACCACATTAAAAAATGTGGTCTTTTGTGTTTTTTTAAAGGATTTGAGCG

TAGCGAAAAATCCTTTTCTTTCTTATCTTGATAATAAGGGTAACTATTGCCGA

TCGTCCATTCCGACAGCATCGCCAGTCACTATGGCGTGCTGCTAGCGCCATT

CGCCATTCAGGCTGCGCAACTGTTGGGAAGGGCGATCGGTGCGGGCCTCT

TCGCTATTACGCCAGCTGGCGAAAGGGGGATGTGCTGCAAGGCGATTAAGT

TGGGTAACGCCAGGGTTTTCCCAGTCACGACGTTGTAAAACGACGGCCAG

TGAATTCGAGCTCAGGCCTTAACTCACATTAATTGCGTTGCGCTCACTGCCC

GCTTTCCAGTCGGGAAACCTGTCGTGCCAGCTGCATTAATGAATCGGCCAA

CGCGCGGGGAGAGGCGGTTTGCGTATTGGGCGCCAGGGTGGTTTTTCTTTT

CACCAGTGAGACGGGCAACAGCTGATTGCCCTTCACCGCCTGGCCCTGAG

AGAGTTGCAGCAAGCGGTCCACGCTGGTTTGCCCCAGCAGGCGAAAATCC

TGTTTGATGGTGGTTGACGGCGGGATATAACATGAGCTGTCTTCGGTATCGT

CGTATCCCACTACCGAGATATCCGCACCAACGCGCAGCCCGGACTCGGTAA

TGGCGCGCATTGCGCCCAGCGCCATCTGATCGTTGGCAACCAGCATCGCAG

TGGGAACGATGCCCTCATTCAGCATTTGCATGGTTTGTTGAAAACCGGACA

TGGCACTCCAGTCGCCTTCCCGTTCCGCTATCGGCTGAATTTGATTGCGAGT

GAGATATTTATGCCAGCCAGCCAGACGCAGACGCGCCGAGACAGAACTTAA

TGGGCCCGCTAACAGCGCGATTTGCTGGTGACCCAATGCGACCAGATGCTC

CACGCCCAGTCGCGTACCGTCTTCATGGGAGAAAATAATACTGTTGATGGG

TGTCTGGTCAGAGACATCAAGAAATAACGCCGGAACATTAGTGCAGGCAG

CTTCCACAGCAATGGCATCCTGGTCATCCAGCGGATAGTTAATGATCAGCCC

ACTGACGCGTTGCGCGAGAAGATTGTGCACCGCCGCTTTACAGGCTTCGAC

GCCGCTTCGTTCTACCATCGACACCACCACGCTGGCACCCAGTTGATCGGC

GCGAGATTTAATCGCCGCGACAATTTGCGACGGCGCGTGCAGGGCCAGAC

TGGAGGTGGCAACGCCAATCAGCAACGACTGTTTGCCCGCCAGTTGTTGT

GCCACGCGGTTGGGAATGTAATTCAGCTCCGCCATCGCCGCTTCCACTTTTT

CCCGCGTTTTCGCAGAAACGTGGCTGGCCTGGTTCACCACGCGGGAAACG

GTCTGATAAGAGACACCGGCATACTCTGCGACATCGTATAACGTTACTGGTT

TCATCAAAATCGTCTCCCTCCGTTTGAATATTTGATTGATCGTAACCAGATG

AAGCACTCTTTCCACTATCCCTACAGTGTTATGGCTTGAACAATCACGAAAC

AATAATTGGTACGTACGATCTTTCAGCCGACTCAAACATCAAATCTTACAAA

TGTAGTCTTTGAAAGTATTACATATGTAAGATTTAAATGCAACCGTTTTTTCG

GAAGGAAATGATGACCTCGTTTCCACCGGAATTAGCTTGGTACCAGCTATT

GTAACATAATCGGTACGGGGGTGAAAAAGCTAACGGAAAAGGGAGCGGAA

AAGAATGATGTAAGCGTGAAAAATTTTTTATCTTATCACTTGAAATTGGAAG

GGAGATTCTTTATTATAAGAATTGTGGAATTGTGAGCGGATAACAATTCCCA

ATTAAAGGAGGAAGGATCACTGATCTACAACAACTGCTTTCATTTCCTGAG

CTCAAGCCTTTTTATCCTGATTACATACAAGTACTTCAGCAACTTCTTCGTG

CTGAATTATCATAAGCTGATCTTCGGAGGCAATTATGTGAGAAATAAGGCGA

ATCTGACGCATACACTTGAAAGCTTTAAGATTGTCTTCAGCATCAAGTTCAA

GAGAAACGGAGAAAATAGCATGACAGAAAGAATCTTCAAGATCAACGGAA

TCGATATCTGTACAGAAAGCTTTGGAAACCCGAAAAATCCGGCAATTCTGC

TTATTATGGGAGCGACATGCTCAATGGTTTATTGGGATGAAGAATTCTGCGA

ACAACTGGCGAATTCAGGAAAATTTGTTATCAGATTCGACAACCGCGATGT

TGGAAGATCAGTTGTTTATGAACCGGGCACAAGCAATTATACAGTGACAAA

TATGGCGGAAGACGCGATTGGCGTGCTGGATGCTTATCATATTAATCAAGCA

CACCTGTTCGGCATGTCACTTGGCGGCATGATTGCGCAAATTGCGGCAGTG

AAACATCCGGAAAGAATTCTTTCACTTACGCTTCTGGCAACATCAATTATTG

GCTCAGATGATAACACGAGAGATCTGCCGCCGATGGATGAATCAATTCTTA

CACATCATGCGAACGGCACACATCTGGATTGGACAAATGAAAAAGTGGTT

GCAGAATACCTGGTGAGCGGCTCAAGACTGCTGTGTGGAAGCAAAAGAAC

ATTTGATGAAATCCGCGTGTACAATCAAGTTAAACAAGAAATCGAGCGCGC

GAATAATCTTCTGTCAATGTTTAATCACGCACTGCTGCAGGGCGATGATGCA

TATGAAGGCGTTCTGCATAGCATTCAGGCACCGACACTTGTGATTCATGGA

ACAGATGATACAGCGCTGCCGTTTGAACATGGACTGGCACTGATTGATGAA

ATTCCGAATAGCGTTCTTCTGACACTTGAAGGCGCAGGACATGAAAATCAT

CCGGATGATTGGGAAGATATTATCCATGCGGTTACAGAACATACAGCAAAA

ATTATCGACAAGTAAGGATCCTCTAGAGTCGACGTCCCCGGGGCAGCCCGC

CTAATGAGCGGGCTTTTTTCACGTCACGCGTCCATGGAGATCTTTGTCTGCA

ACTGAAAAGTTTATACCTTACCTGGAACAAATGGTTGAAACATACGAGGCT

AATATCGGCTTATTAGGAATAGTCCCTGTACTAATAAAATCAGGTGGATCAG

TTGATCAGTATATTTTGGACGAAGCTCGGAAAGAATTTGGAGATGACTTGC

TTAATTCCACAATTAAATTAAGGGAAAGAATAAAGCGATTTGATGTTCAAG

GAATCACGGAAGAAGATACTCATGATAAAGAAGCTCTAAAACTATTCAATA

ACCTTACAATGGAATTGATCGAAAGGGTGGAAGGTTAATGGTACGAAAATT

AGGGGATCTACCTAGAAAGCCACAAGGCGATAGGTCAAGCTTAAAGAACC

CTTACATGGATCTTACAGATTCTGAAAGTAAAGAAACAACAGAGGTTAAAC

AAACAGAACCAAAAAGAAAAAAAGCATTGTTGAAAACAATGAAAGTTGA

TGTTTCAATCCATAATAAGATTAAATCGCTGCACGAAATTCTGGCAGCATCC

GAAGGGAATTCATATTACTTAGAGGATACTATTGAGAGAGCTATTGATAAGA

TGGTTGAGACATTACCTGAGAGCCAAAAAACTTTTTATGAATATGAATTAAA

AAAAAGAACCAACAAAGGCTGAGACAGACTCCAAACGAGTCTGTTTTTTT

AAAAAAAATATTAGGAGCATTGAATATATATTAGAGAATTAAGAAAGACATG

GGAATAAAAATATTTTAAATCCAGTAAAAATATGATAAGATTATTTCAGAATA

TGAAGAACTCTGTTTGTTTTTGATGAAAAAACAAACAAAAAAAATCCACCT

AACGGAATCTCAATTTAACTAACAGCGGCCAAACTGAGAAGTTAAATTTGA

GAAGGGGAAAAGGCGGATTTATACTTGTATTTAACTATCTCCATTTTAACAT

TTTATTAAACCCCATACAAGTGAAAATCCTCTTTTACACTGTTCCTTTAGGT

GATCGCGGAGGGACATTATGAGTGAAGTAAACCTAAAAGGAAATACAGAT

GAATTAGTGTATTATCGACAGCAAACCACTGGAAATAAAATCGCCAGGAAG

AGAATCAAAAAAGGGAAAGAAGAAGTTTATTATGTTGCTGAAACGGAAGA

GAAGATATGGACAGAAGAGCAAATAAAAAACTTTTCTTTAGACAAATTTGG

TACGCATATACCTTACATAGAAGGTCATTATACAATCTTAAATAATTACTTCTT

TGATTTTTGGGGCTATTTTTTAGGTGCTGAAGGAATTGCGCTCTATGCTCAC

CTAACTCGTTATGCATACGGCAGCAAAGACTTTTGCTTTCCTAGTCTACAAA

CAATCGCTAAAAAAATGGACAAGACTCCTGTTACAGTTAGAGGCTACTTGA

AACTGCTTGAAAGGTACGGTTTTATTTGGAAGGTAAACGTCCGTAATAAAA

CCAAGGATAACACAGAGGAATCCCCGATTTTTAAGATTAGACGTAAGGTTC

CTTTGCTTTCAGAAGAACTTTTAAATGGAAACCCTAATATTGAAATTCCAGA

TGACGAGGAAGCACATGTAAAGAAGGCTTTAAAAAAGGAAAAAGAGGGT

CTTCCAAAGGTTTTGAAAAAAGAGCACGATGAATTTGTTAAAAAAATGATG

GATGAGTCAGAAACAATTAATATTCCAGAGGCCTTACAATATGACACAATGT

ATGAAGATATACTCAGTAAAGGAGAAATTCGAAAAGAAATCAAAAAACAA

ATACCTAATCCTACAACATCTTTTGAGAGTATATCAATGACAACTGAAGAGG

AAAAAGTCGACAGTACTTTAAAAAGCGAAATGCAAAATCGTGTCTCTAAG

CCTTCTTTTGATACCTGGTTTAAAAACACTAAGATCAAAATTGAAAATAAAA

ATTGTTTATTACTTGTACCGAGTGAATTTGCATTTGAATGGATTAAGAAAAG

ATATTTAGAAACAATTAAAACAGTCCTTGAAGAAGCTGGATATGTTTTCGAA

AAAATCGAACTAAGAAAAGTGCAATAAACTGCTGAAGTATTTCAGCAGTTT

TTTTTATTTAGAAATAGTGAAAAAAATATAATCAGGGAGGTATCAATATTTAA

TGAGTACTGATTTAAATTTATTTAGACTGGAATTAATAATTAACACGTAGACT

AATTAAAATTTAATGAGGGATAAAGAGGATACAAAAATATTAATTTCAATCC

CTATTAAATTTTAACAAGGGGGGGATTAAAATTTAATTAGAGGTTTATCCAC

AAGAAAAGACCCTAATAAAATTTTTACTAGGGTTATAACACTGATTAATTTC

TTAATGGGGGAGGGATTAAAATTTAATGACAAAGAAAACAATCTTTTAAGA

AAAGCTTTTAAAAGATAATAATAAAAAGAGCTTTGCGATTAAGCAAAACTC

TTTACTTTTTCATTGACATTATCAAATTCATCGATTTCAAATTGTTGTTGTATC

ATAAAGTTAATTCTGTTTTGCACAACCTTTTCAGGAATATAAAACACATCTG

AGGCTTGTTTTATAAACTCAGGGTCGCTAAAGTCAATGTAACGTAGCATATG

ATATGGTATAGCTTCCACCCAAGTTAGCCTTTCTGCTTCTTCTGAATGTTTTT

CATATACTTCCATGGGTATCTCTAAATGATTTTCCTCATGTAGCAAGGTATGA

GCAAAAAGTTTATGGAATTGATAGTTCCTCTCTTTTTCTTCAACTTTTTTATC

TAAAACAAACACTTTAACATCTGAGTCAATGTAAGCATAAGATGTTTTTCCA

GTCATAATTTCAATCCCAAATCTTTTAGACAGAAATTCTGGACGTAAATCTT

TTGGTGAAAGAATTTTTTTATGTAGCAATATATCCGATACAGCACCTTCTAAA

AGCGTTGGTGAATAGGGCATTTTACCTATCTCCTCTCATTTTGTGGAATAAA

AATAGTCATATTCGTCCATCTACCTATCCTATTATCGAACAGTTGAACTTTTT

AATCAAGGATCAGTCCTTTTTTTCATTATTCTTAAACTGTGCTCTTAACTTTA

ACAACTCGATTTGTTTTTCCAGATCTCGAGGGTAACTAGCCTCGCCGATCCC

GCAAGAGGCCCGGCAGTCAGGTGGCACTTTTCGGGGAAATGTGCGCGGAA

CCCCTATTTGTTTATTTTTCTAAATACATTCAAATATGTATCCGCTCATGAGAC

AATAACCCTGATAAATGCTTCAATAATATTGAAAAAGGAAGAGTATGAGTAT

TCAACATTTCCGTGTCGCCCTTATTCCCTTTTTTGCGGCATTTTGCCTTCCTG

TTTTTGCTCACCCAGAAACGCTGGTGAAAGTAAAAGATGCTGAAGATCAGT

TGGGTGCACGAGTGGGTTACATCGAACTGGATCTCAACAGCGGTAAGATCC

TTGAGAGTTTTCGCCCCGAAGAACGTTTTCCAATGATGAGCACTTTTAAAG

TTCTGCTATGTGGCGCGGTATTATCCCGTATTGACGCCGGGCAAGAGCAACT

CGGTCGCCGCATACACTATTCTCAGAATGACTTGGTTGAGTACTCACCAGTC

ACAGAAAAGCATCTTACGGATGGCATGACAGTAAGAGAATTATGCAGTGCT

GCCATAACCATGAGTGATAACACTGCGGCCAACTTACTTCTGACAACGATC

GGAGGACCGAAGGAGCTAACCGCTTTTTTGCACAACATGGGGGATCATGTA

ACTCGCCTTGATCGTTGGGAACCGGAGCTGAATGAAGCCATACCAAACGA

CGAGCGTGACACCACGATGCCTGTAGCAATGGCAACAACGTTGCGCAAAC

TATTAACTGGCGAACTACTTACTCTAGCTTCCCGGCAACAATTAATAGACTG

GATGGAGGCGGATAAAGTTGCAGGACCACTTCTGCGCTCGGCCCTTCCGGC

TGGCTGGTTTATTGCTGATAAATCTGGAGCCGGTGAGCGTGGGTCTCGCGG

TATCATTGCAGCACTGGGGCCAGATGGTAAGCCCTCCCGTATCGTAGTTATC

TACACGACGGGGAGTCAGGCAACTATGGATGAACGAAATAGACAGATCGC

TGAGATAGGTGCCTCACTGATTAAGCATTGGTAACTGTCAGACCAAGTTTA

CTCATATATACTTTAGATTGATTTAAAACTTCATTTTTAATTTAAAAGGATCTA

GGTGAAGATCCTTTTTGATAATCTCATGACCAAAATCCCTTAACGTGAGTTT

TCGTTCCACTGAGCGTCAGACCCCGTAGAAAAGATCAAAGGATCTTCTTGA

GATCCTTTTTTTCTGCGCGTAATCTGCTGCTTGCAAACAAAAAAACCACCG

CTACCAGCGGTGGTTTGTTTGCCGGATCAAGAGCTACCAACTCTTTTTCCG

AAGGTAACTGGCTTCAGCAGAGCGCAGATACCAAATACTGTCCTTCTAGTG

TAGCCGTAGTTAGGCCACCACTTCAAGAACTCTGTAGCACCGCCTACATAC

CTCGCTCTGCTAATCCTGTTACCAGTGGCTGCTGCCAGTGGCGATAAGTCGT

GTCTTACCGGGTTGGACTCAAGACGATAGTTACCGGATAAGGCGCAGCGGT

CGGGCTGAACGGGGGGTTCGTGCACACAGCCCAGCTTGGAGCGAACGAC

CTACACCGAACTGAGATACCTACAGCGTGAGCTATGAGAAAGCGCCACGCT

TCCCGAAGGGAGAAAGGCGGACAGGTATCCGGTAAGCGGCAGGGTCGGA

ACAGGAGAGCGCACGAGGGAGCTTCCAGGGGGAAACGCCTGGTATCTTTA

TAGTCCTGTCGGGTTTCGCCACCTCTGACTTGAGCGTCGATTTTTGTGATGC

TCGTCAGGGGGGCGGAGCCTATGGAAAAACGCCAGCAACGCGGCCTTTTT

ACGGTTCCTGGCCTTTTGCTGGCCTTTTGCTCACATGTTCTTTCCTGCGTTAT

CCCCTGATTCTGTGGATAACCGTATTACCGCCTTTGAGTGAGCTGATACCGC

TCGCCGCAGCCGAACGACCGAGCGCAGCGAGTCAGTGAGCGAGGAAGCG

GAAGAGCGCCCAATACGCATGC

pHT43-BCB-1:

TTAAGTTATTGGTATGACTGGTTTTAAGCGCAAAAAAAGTTGCTTTTTCGTA

CCTATTAATGTATCGTTTTAGAAAACCGACTGTAAAAAGTACAGTCGGCATT

ATCTCATATTATAAAAGCCAGTCATTAGGCCTATCTGACAATTCCTGAATAGA

GTTCATAAACAATCCTGCATGATAACCATCACAAACAGAATGATGTACCTGT

AAAGATAGCGGTAAATATATTGAATTACCTTTATTAATGAATTTTCCTGCTGT

AATAATGGGTAGAAGGTAATTACTATTATTATTGATATTTAAGTTAAACCCAG

TAAATGAAGTCCATGGAATAATAGAAAGAGAAAAAGCATTTTCAGGTATAG

GTGTTTTGGGAAACAATTTCCCCGAACCATTATATTTCTCTACATCAGAAAG

GTATAAATCATAAAACTCTTTGAAGTCATTCTTTACAGGAGTCCAAATACCA

GAGAATGTTTTAGATACACCATCAAAAATTGTATAAAGTGGCTCTAACTTAT

CCCAATAACCTAACTCTCCGTCGCTATTGTAACCAGTTCTAAAAGCTGTATT

TGAGTTTATCACCCTTGTCACTAAGAAAATAAATGCAGGGTAAAATTTATAT

CCTTCTTGTTTTATGTTTCGGTATAAAACACTAATATCAATTTCTGTGGTTATA

CTAAAAGTCGTTTGTTGGTTCAAATAATGATTAAATATCTCTTTTCTCTTCCA

ATTGTCTAAATCAATTTTATTAAAGTTCATTTGATATGCCTCCTAAATTTTTAT

CTAAAGTGAATTTAGGAGGCTTACTTGTCTGCTTTCTTCATTAGAATCAATC

CTTTTTTAAAAGTCAATATTACTGTAACATAAATATATATTTTAAAAATATCCC

ACTTTATCCAATTTTCGTTTGTTGAACTAATGGGTGCTTTAGTTGAAGAATA

AAGACCACATTAAAAAATGTGGTCTTTTGTGTTTTTTTAAAGGATTTGAGCG

TAGCGAAAAATCCTTTTCTTTCTTATCTTGATAATAAGGGTAACTATTGCCGA

TCGTCCATTCCGACAGCATCGCCAGTCACTATGGCGTGCTGCTAGCGCCATT

CGCCATTCAGGCTGCGCAACTGTTGGGAAGGGCGATCGGTGCGGGCCTCT

TCGCTATTACGCCAGCTGGCGAAAGGGGGATGTGCTGCAAGGCGATTAAGT

TGGGTAACGCCAGGGTTTTCCCAGTCACGACGTTGTAAAACGACGGCCAG

TGAATTCGAGCTCAGGCCTTAACTCACATTAATTGCGTTGCGCTCACTGCCC

GCTTTCCAGTCGGGAAACCTGTCGTGCCAGCTGCATTAATGAATCGGCCAA

CGCGCGGGGAGAGGCGGTTTGCGTATTGGGCGCCAGGGTGGTTTTTCTTTT

CACCAGTGAGACGGGCAACAGCTGATTGCCCTTCACCGCCTGGCCCTGAG

AGAGTTGCAGCAAGCGGTCCACGCTGGTTTGCCCCAGCAGGCGAAAATCC

TGTTTGATGGTGGTTGACGGCGGGATATAACATGAGCTGTCTTCGGTATCGT

CGTATCCCACTACCGAGATATCCGCACCAACGCGCAGCCCGGACTCGGTAA

TGGCGCGCATTGCGCCCAGCGCCATCTGATCGTTGGCAACCAGCATCGCAG

TGGGAACGATGCCCTCATTCAGCATTTGCATGGTTTGTTGAAAACCGGACA

TGGCACTCCAGTCGCCTTCCCGTTCCGCTATCGGCTGAATTTGATTGCGAGT

GAGATATTTATGCCAGCCAGCCAGACGCAGACGCGCCGAGACAGAACTTAA

TGGGCCCGCTAACAGCGCGATTTGCTGGTGACCCAATGCGACCAGATGCTC

CACGCCCAGTCGCGTACCGTCTTCATGGGAGAAAATAATACTGTTGATGGG

TGTCTGGTCAGAGACATCAAGAAATAACGCCGGAACATTAGTGCAGGCAG

CTTCCACAGCAATGGCATCCTGGTCATCCAGCGGATAGTTAATGATCAGCCC

ACTGACGCGTTGCGCGAGAAGATTGTGCACCGCCGCTTTACAGGCTTCGAC

GCCGCTTCGTTCTACCATCGACACCACCACGCTGGCACCCAGTTGATCGGC

GCGAGATTTAATCGCCGCGACAATTTGCGACGGCGCGTGCAGGGCCAGAC

TGGAGGTGGCAACGCCAATCAGCAACGACTGTTTGCCCGCCAGTTGTTGT

GCCACGCGGTTGGGAATGTAATTCAGCTCCGCCATCGCCGCTTCCACTTTTT

CCCGCGTTTTCGCAGAAACGTGGCTGGCCTGGTTCACCACGCGGGAAACG

GTCTGATAAGAGACACCGGCATACTCTGCGACATCGTATAACGTTACTGGTT

TCATCAAAATCGTCTCCCTCCGTTTGAATATTTGATTGATCGTAACCAGATG

AAGCACTCTTTCCACTATCCCTACAGTGTTATGGCTTGAACAATCACGAAAC

AATAATTGGTACGTACGATCTTTCAGCCGACTCAAACATCAAATCTTACAAA

TGTAGTCTTTGAAAGTATTACATATGTAAGATTTAAATGCAACCGTTTTTTCG

GAAGGAAATGATGACCTCGTTTCCACCGGAATTAGCTTGGTACCAGCTATT

GTAACATAATCGGTACGGGGGTGAAAAAGCTAACGGAAAAGGGAGCGGAA

AAGAATGATGTAAGCGTGAAAAATTTTTTATCTTATCACTTGAAATTGGAAG

GGAGATTCTTTATTATAAGAATTGTGGAATTGTGAGCGGATAACAATTCCCA

ATTAAAGGAGGAAGGATCAATGACGGAAAAGATCATCAAGATCATCAAAAT

CAACAAGATCGACATCTGCACAGAATCATTTGGCAATAGCGCAGATCCGGC

AGTGCTGCTGATTATGGGAGCAATGTGTAGCATGGTTTATTGGGATGAAGAA

TTCTGCCAGCAACTGGCAGATACAGGCAGATATGTGATTAGATATGACAAC

AGAGACGTCGGAAGAAGCACAACATATGAACCGGGCTCATCACATTATACA

GTGGTTGATATGGCGGATGATGCGATTGGCGTGCTTGATGCATATCATATTGA

TGAAGCGCATATCGTGGGAATGTCACTTGGCGGAATGATTGCGCAGATTGT

TGCACTGAGAAATCCGGAAAGAGTTATTAGCATTACGCTGATTGCGTCAGG

CATTTTTGGCTCAGAAGATAATGATAGAAACCTGCCGCCGATTGATGAAAA

AATTCTGGCGTATCATACGAACGCAGCGAAACTGAATTGGAGCGATGAAGA

AAGCGTTGCGAATTATCTGGTGGCAGGAAGCGCACTGCTGTGCGGATCAA

AACATAAATTTGATAAGAAGCGTGCATACAAGCAGGTTGAAAATGAAATTA

AGCGCGCAAATAACCTGCTGAGCATGTTTAATCATAGCCTGCTGAAAGGCG

AAGATTCATATGAAGGAAGACTTAAAGAGATCAACATCCCGACACTGGTTA

TTCATGGAACAGAAGATACAGTGCTTTCATATGAACACGGACTGGCACTTG

TTAATGAAATTCCGCATGCGGTTCTGCTGCCGCTGGAAAGAAGCGGACATG

AAATTCATTGTGATGATTGGAACCACATCATCAATGCAATTCTTAACCACAC

AAGCGTTCTGTAAGGATCCTCTAGAGTCGACGTCCCCGGGGCAGCCCGCCT

AATGAGCGGGCTTTTTTCACGTCACGCGTCCATGGAGATCTTTGTCTGCAA

CTGAAAAGTTTATACCTTACCTGGAACAAATGGTTGAAACATACGAGGCTA

ATATCGGCTTATTAGGAATAGTCCCTGTACTAATAAAATCAGGTGGATCAGTT

GATCAGTATATTTTGGACGAAGCTCGGAAAGAATTTGGAGATGACTTGCTT

AATTCCACAATTAAATTAAGGGAAAGAATAAAGCGATTTGATGTTCAAGGA

ATCACGGAAGAAGATACTCATGATAAAGAAGCTCTAAAACTATTCAATAAC

CTTACAATGGAATTGATCGAAAGGGTGGAAGGTTAATGGTACGAAAATTAG

GGGATCTACCTAGAAAGCCACAAGGCGATAGGTCAAGCTTAAAGAACCCTT

ACATGGATCTTACAGATTCTGAAAGTAAAGAAACAACAGAGGTTAAACAA

ACAGAACCAAAAAGAAAAAAAGCATTGTTGAAAACAATGAAAGTTGATGT

TTCAATCCATAATAAGATTAAATCGCTGCACGAAATTCTGGCAGCATCCGAA

GGGAATTCATATTACTTAGAGGATACTATTGAGAGAGCTATTGATAAGATGG

TTGAGACATTACCTGAGAGCCAAAAAACTTTTTATGAATATGAATTAAAAA

AAAGAACCAACAAAGGCTGAGACAGACTCCAAACGAGTCTGTTTTTTTAA

AAAAAATATTAGGAGCATTGAATATATATTAGAGAATTAAGAAAGACATGGG

AATAAAAATATTTTAAATCCAGTAAAAATATGATAAGATTATTTCAGAATATG

AAGAACTCTGTTTGTTTTTGATGAAAAAACAAACAAAAAAAATCCACCTA

ACGGAATCTCAATTTAACTAACAGCGGCCAAACTGAGAAGTTAAATTTGAG

AAGGGGAAAAGGCGGATTTATACTTGTATTTAACTATCTCCATTTTAACATTT

TATTAAACCCCATACAAGTGAAAATCCTCTTTTACACTGTTCCTTTAGGTGA

TCGCGGAGGGACATTATGAGTGAAGTAAACCTAAAAGGAAATACAGATGA

ATTAGTGTATTATCGACAGCAAACCACTGGAAATAAAATCGCCAGGAAGAG

AATCAAAAAAGGGAAAGAAGAAGTTTATTATGTTGCTGAAACGGAAGAGA

AGATATGGACAGAAGAGCAAATAAAAAACTTTTCTTTAGACAAATTTGGTA

CGCATATACCTTACATAGAAGGTCATTATACAATCTTAAATAATTACTTCTTT

GATTTTTGGGGCTATTTTTTAGGTGCTGAAGGAATTGCGCTCTATGCTCACC

TAACTCGTTATGCATACGGCAGCAAAGACTTTTGCTTTCCTAGTCTACAAAC

AATCGCTAAAAAAATGGACAAGACTCCTGTTACAGTTAGAGGCTACTTGAA

ACTGCTTGAAAGGTACGGTTTTATTTGGAAGGTAAACGTCCGTAATAAAAC

CAAGGATAACACAGAGGAATCCCCGATTTTTAAGATTAGACGTAAGGTTCC

TTTGCTTTCAGAAGAACTTTTAAATGGAAACCCTAATATTGAAATTCCAGAT

GACGAGGAAGCACATGTAAAGAAGGCTTTAAAAAAGGAAAAAGAGGGTC

TTCCAAAGGTTTTGAAAAAAGAGCACGATGAATTTGTTAAAAAAATGATGG

ATGAGTCAGAAACAATTAATATTCCAGAGGCCTTACAATATGACACAATGTA

TGAAGATATACTCAGTAAAGGAGAAATTCGAAAAGAAATCAAAAAACAAA

TACCTAATCCTACAACATCTTTTGAGAGTATATCAATGACAACTGAAGAGGA

AAAAGTCGACAGTACTTTAAAAAGCGAAATGCAAAATCGTGTCTCTAAGCC

TTCTTTTGATACCTGGTTTAAAAACACTAAGATCAAAATTGAAAATAAAAAT

TGTTTATTACTTGTACCGAGTGAATTTGCATTTGAATGGATTAAGAAAAGAT

ATTTAGAAACAATTAAAACAGTCCTTGAAGAAGCTGGATATGTTTTCGAAA

AAATCGAACTAAGAAAAGTGCAATAAACTGCTGAAGTATTTCAGCAGTTTT

TTTTATTTAGAAATAGTGAAAAAAATATAATCAGGGAGGTATCAATATTTAAT

GAGTACTGATTTAAATTTATTTAGACTGGAATTAATAATTAACACGTAGACTA

ATTAAAATTTAATGAGGGATAAAGAGGATACAAAAATATTAATTTCAATCCC

TATTAAATTTTAACAAGGGGGGGATTAAAATTTAATTAGAGGTTTATCCACA

AGAAAAGACCCTAATAAAATTTTTACTAGGGTTATAACACTGATTAATTTCTT

AATGGGGGAGGGATTAAAATTTAATGACAAAGAAAACAATCTTTTAAGAAA

AGCTTTTAAAAGATAATAATAAAAAGAGCTTTGCGATTAAGCAAAACTCTTT

ACTTTTTCATTGACATTATCAAATTCATCGATTTCAAATTGTTGTTGTATCATA

AAGTTAATTCTGTTTTGCACAACCTTTTCAGGAATATAAAACACATCTGAGG

CTTGTTTTATAAACTCAGGGTCGCTAAAGTCAATGTAACGTAGCATATGATAT

GGTATAGCTTCCACCCAAGTTAGCCTTTCTGCTTCTTCTGAATGTTTTTCATA

TACTTCCATGGGTATCTCTAAATGATTTTCCTCATGTAGCAAGGTATGAGCA

AAAAGTTTATGGAATTGATAGTTCCTCTCTTTTTCTTCAACTTTTTTATCTAA

AACAAACACTTTAACATCTGAGTCAATGTAAGCATAAGATGTTTTTCCAGTC

ATAATTTCAATCCCAAATCTTTTAGACAGAAATTCTGGACGTAAATCTTTTG

GTGAAAGAATTTTTTTATGTAGCAATATATCCGATACAGCACCTTCTAAAAG

CGTTGGTGAATAGGGCATTTTACCTATCTCCTCTCATTTTGTGGAATAAAAAT

AGTCATATTCGTCCATCTACCTATCCTATTATCGAACAGTTGAACTTTTTAAT

CAAGGATCAGTCCTTTTTTTCATTATTCTTAAACTGTGCTCTTAACTTTAACA

ACTCGATTTGTTTTTCCAGATCTCGAGGGTAACTAGCCTCGCCGATCCCGCA

AGAGGCCCGGCAGTCAGGTGGCACTTTTCGGGGAAATGTGCGCGGAACCC

CTATTTGTTTATTTTTCTAAATACATTCAAATATGTATCCGCTCATGAGACAAT

AACCCTGATAAATGCTTCAATAATATTGAAAAAGGAAGAGTATGAGTATTCA

ACATTTCCGTGTCGCCCTTATTCCCTTTTTTGCGGCATTTTGCCTTCCTGTTT

TTGCTCACCCAGAAACGCTGGTGAAAGTAAAAGATGCTGAAGATCAGTTG

GGTGCACGAGTGGGTTACATCGAACTGGATCTCAACAGCGGTAAGATCCTT

GAGAGTTTTCGCCCCGAAGAACGTTTTCCAATGATGAGCACTTTTAAAGTT

CTGCTATGTGGCGCGGTATTATCCCGTATTGACGCCGGGCAAGAGCAACTC

GGTCGCCGCATACACTATTCTCAGAATGACTTGGTTGAGTACTCACCAGTC

ACAGAAAAGCATCTTACGGATGGCATGACAGTAAGAGAATTATGCAGTGCT

GCCATAACCATGAGTGATAACACTGCGGCCAACTTACTTCTGACAACGATC

GGAGGACCGAAGGAGCTAACCGCTTTTTTGCACAACATGGGGGATCATGTA

ACTCGCCTTGATCGTTGGGAACCGGAGCTGAATGAAGCCATACCAAACGA

CGAGCGTGACACCACGATGCCTGTAGCAATGGCAACAACGTTGCGCAAAC

TATTAACTGGCGAACTACTTACTCTAGCTTCCCGGCAACAATTAATAGACTG

GATGGAGGCGGATAAAGTTGCAGGACCACTTCTGCGCTCGGCCCTTCCGGC

TGGCTGGTTTATTGCTGATAAATCTGGAGCCGGTGAGCGTGGGTCTCGCGG

TATCATTGCAGCACTGGGGCCAGATGGTAAGCCCTCCCGTATCGTAGTTATC

TACACGACGGGGAGTCAGGCAACTATGGATGAACGAAATAGACAGATCGC

TGAGATAGGTGCCTCACTGATTAAGCATTGGTAACTGTCAGACCAAGTTTA

CTCATATATACTTTAGATTGATTTAAAACTTCATTTTTAATTTAAAAGGATCTA

GGTGAAGATCCTTTTTGATAATCTCATGACCAAAATCCCTTAACGTGAGTTT

TCGTTCCACTGAGCGTCAGACCCCGTAGAAAAGATCAAAGGATCTTCTTGA

GATCCTTTTTTTCTGCGCGTAATCTGCTGCTTGCAAACAAAAAAACCACCG

CTACCAGCGGTGGTTTGTTTGCCGGATCAAGAGCTACCAACTCTTTTTCCG

AAGGTAACTGGCTTCAGCAGAGCGCAGATACCAAATACTGTCCTTCTAGTG

TAGCCGTAGTTAGGCCACCACTTCAAGAACTCTGTAGCACCGCCTACATAC

CTCGCTCTGCTAATCCTGTTACCAGTGGCTGCTGCCAGTGGCGATAAGTCGT

GTCTTACCGGGTTGGACTCAAGACGATAGTTACCGGATAAGGCGCAGCGGT

CGGGCTGAACGGGGGGTTCGTGCACACAGCCCAGCTTGGAGCGAACGAC

CTACACCGAACTGAGATACCTACAGCGTGAGCTATGAGAAAGCGCCACGCT

TCCCGAAGGGAGAAAGGCGGACAGGTATCCGGTAAGCGGCAGGGTCGGA

ACAGGAGAGCGCACGAGGGAGCTTCCAGGGGGAAACGCCTGGTATCTTTA

TAGTCCTGTCGGGTTTCGCCACCTCTGACTTGAGCGTCGATTTTTGTGATGC

TCGTCAGGGGGGCGGAGCCTATGGAAAAACGCCAGCAACGCGGCCTTTTT

ACGGTTCCTGGCCTTTTGCTGGCCTTTTGCTCACATGTTCTTTCCTGCGTTAT

CCCCTGATTCTGTGGATAACCGTATTACCGCCTTTGAGTGAGCTGATACCGC

TCGCCGCAGCCGAACGACCGAGCGCAGCGAGTCAGTGAGCGAGGAAGCG

GAAGAGCGCCCAATACGCATGC

pHT43-BRB-1:

TTAAGTTATTGGTATGACTGGTTTTAAGCGCAAAAAAAGTTGCTTTTTCGTA

CCTATTAATGTATCGTTTTAGAAAACCGACTGTAAAAAGTACAGTCGGCATT

ATCTCATATTATAAAAGCCAGTCATTAGGCCTATCTGACAATTCCTGAATAGA

GTTCATAAACAATCCTGCATGATAACCATCACAAACAGAATGATGTACCTGT

AAAGATAGCGGTAAATATATTGAATTACCTTTATTAATGAATTTTCCTGCTGT

AATAATGGGTAGAAGGTAATTACTATTATTATTGATATTTAAGTTAAACCCAG

TAAATGAAGTCCATGGAATAATAGAAAGAGAAAAAGCATTTTCAGGTATAG

GTGTTTTGGGAAACAATTTCCCCGAACCATTATATTTCTCTACATCAGAAAG

GTATAAATCATAAAACTCTTTGAAGTCATTCTTTACAGGAGTCCAAATACCA

GAGAATGTTTTAGATACACCATCAAAAATTGTATAAAGTGGCTCTAACTTAT

CCCAATAACCTAACTCTCCGTCGCTATTGTAACCAGTTCTAAAAGCTGTATT

TGAGTTTATCACCCTTGTCACTAAGAAAATAAATGCAGGGTAAAATTTATAT

CCTTCTTGTTTTATGTTTCGGTATAAAACACTAATATCAATTTCTGTGGTTATA

CTAAAAGTCGTTTGTTGGTTCAAATAATGATTAAATATCTCTTTTCTCTTCCA

ATTGTCTAAATCAATTTTATTAAAGTTCATTTGATATGCCTCCTAAATTTTTAT

CTAAAGTGAATTTAGGAGGCTTACTTGTCTGCTTTCTTCATTAGAATCAATC

CTTTTTTAAAAGTCAATATTACTGTAACATAAATATATATTTTAAAAATATCCC

ACTTTATCCAATTTTCGTTTGTTGAACTAATGGGTGCTTTAGTTGAAGAATA

AAGACCACATTAAAAAATGTGGTCTTTTGTGTTTTTTTAAAGGATTTGAGCG

TAGCGAAAAATCCTTTTCTTTCTTATCTTGATAATAAGGGTAACTATTGCCGA

TCGTCCATTCCGACAGCATCGCCAGTCACTATGGCGTGCTGCTAGCGCCATT

CGCCATTCAGGCTGCGCAACTGTTGGGAAGGGCGATCGGTGCGGGCCTCT

TCGCTATTACGCCAGCTGGCGAAAGGGGGATGTGCTGCAAGGCGATTAAGT

TGGGTAACGCCAGGGTTTTCCCAGTCACGACGTTGTAAAACGACGGCCAG

TGAATTCGAGCTCAGGCCTTAACTCACATTAATTGCGTTGCGCTCACTGCCC

GCTTTCCAGTCGGGAAACCTGTCGTGCCAGCTGCATTAATGAATCGGCCAA

CGCGCGGGGAGAGGCGGTTTGCGTATTGGGCGCCAGGGTGGTTTTTCTTTT

CACCAGTGAGACGGGCAACAGCTGATTGCCCTTCACCGCCTGGCCCTGAG

AGAGTTGCAGCAAGCGGTCCACGCTGGTTTGCCCCAGCAGGCGAAAATCC

TGTTTGATGGTGGTTGACGGCGGGATATAACATGAGCTGTCTTCGGTATCGT

CGTATCCCACTACCGAGATATCCGCACCAACGCGCAGCCCGGACTCGGTAA

TGGCGCGCATTGCGCCCAGCGCCATCTGATCGTTGGCAACCAGCATCGCAG

TGGGAACGATGCCCTCATTCAGCATTTGCATGGTTTGTTGAAAACCGGACA

TGGCACTCCAGTCGCCTTCCCGTTCCGCTATCGGCTGAATTTGATTGCGAGT

GAGATATTTATGCCAGCCAGCCAGACGCAGACGCGCCGAGACAGAACTTAA

TGGGCCCGCTAACAGCGCGATTTGCTGGTGACCCAATGCGACCAGATGCTC

CACGCCCAGTCGCGTACCGTCTTCATGGGAGAAAATAATACTGTTGATGGG

TGTCTGGTCAGAGACATCAAGAAATAACGCCGGAACATTAGTGCAGGCAG

CTTCCACAGCAATGGCATCCTGGTCATCCAGCGGATAGTTAATGATCAGCCC

ACTGACGCGTTGCGCGAGAAGATTGTGCACCGCCGCTTTACAGGCTTCGAC

GCCGCTTCGTTCTACCATCGACACCACCACGCTGGCACCCAGTTGATCGGC

GCGAGATTTAATCGCCGCGACAATTTGCGACGGCGCGTGCAGGGCCAGAC

TGGAGGTGGCAACGCCAATCAGCAACGACTGTTTGCCCGCCAGTTGTTGT

GCCACGCGGTTGGGAATGTAATTCAGCTCCGCCATCGCCGCTTCCACTTTTT

CCCGCGTTTTCGCAGAAACGTGGCTGGCCTGGTTCACCACGCGGGAAACG

GTCTGATAAGAGACACCGGCATACTCTGCGACATCGTATAACGTTACTGGTT

TCATCAAAATCGTCTCCCTCCGTTTGAATATTTGATTGATCGTAACCAGATG

AAGCACTCTTTCCACTATCCCTACAGTGTTATGGCTTGAACAATCACGAAAC

AATAATTGGTACGTACGATCTTTCAGCCGACTCAAACATCAAATCTTACAAA

TGTAGTCTTTGAAAGTATTACATATGTAAGATTTAAATGCAACCGTTTTTTCG

GAAGGAAATGATGACCTCGTTTCCACCGGAATTAGCTTGGTACCAGCTATT

GTAACATAATCGGTACGGGGGTGAAAAAGCTAACGGAAAAGGGAGCGGAA

AAGAATGATGTAAGCGTGAAAAATTTTTATCTTATCACTTGAAATTGGAAG

GGAGATTCTTTATTATAAGAATTGTGGAATTGTGAGCGGATAACAATTCCCA

ATTAAAGGAGGAAGGATCAATGGCTGAACAGATTCTGAAAGTTAACGGAG

TGGAAATTTGCGCGGAAAGCTTTGGCAAACCGACAGATCCGGCGATTCTG

CTTATTATGGGAGCGCAAATGAGCATGCTTTGGTGGGAAGAAGAATTTTGC

CAAAGAATTGCAGACGCAGGAAGATTTGTTATTAGATTTGACAACCGCGAC

GTGGGAAGATCAACAACATATGAAGTTGGACAACCGGGCTATACATTTGAA

GATATGGCAGATGATGCGGTGCATGTGCTGGATGCGTTTGGCGTTCAACAG

GCACATTTTGTGGGCATGAGCATGGGCGGCATGCTGACACAAATGATTGCA

CTGAGACATCCGGAAAGAGTTAGAACAATTACACTGCATGCGACATCAAAT

TTCGCACCGGGCCTGCCGCCGATTGATGAAAAACTTATGGAATTTTTCAGC

AAGATGGGCGAAATTAACTGGGAAGATGAAAAAGAAGCGCTGGAAGCAG

CAGTTGCAAGCTGGAAAGTGCTGAGCGGCAGCAAACATCCGTTTGATGAA

AGCAGAGTTAGAGAACTGGCAAAAATTGATATCGCGAGATCAAATCACTAC

GCAAGCAGAAATAACCATGCGTTTGTGACAGCATCAGAACCGTATCTTCTT

AGAACAGCAGAAATTGCGGTTCCGGCGCTTGTTATTCATGGCACAGAAGAT

CTGCTGATTCCGTTTGCGCATGCACTGCATCTTGCGAATACAATTCCGGGAG

CGGTTCTTCTGACACTTGAAGGAACAGGCCATGAACTTCCGTATGGCGATT

GGGATGTGGTTATTGAAGCAATTCTTAAGCACACAAGCGGAAGAAGAGTTA

GACTGTAAGGATCCTCTAGAGTCGACGTCCCCGGGGCAGCCCGCCTAATGA

GCGGGCTTTTTTCACGTCACGCGTCCATGGAGATCTTTGTCTGCAACTGAA

AAGTTTATACCTTACCTGGAACAAATGGTTGAAACATACGAGGCTAATATCG

GCTTATTAGGAATAGTCCCTGTACTAATAAAATCAGGTGGATCAGTTGATCA

GTATATTTTGGACGAAGCTCGGAAAGAATTTGGAGATGACTTGCTTAATTCC

ACAATTAAATTAAGGGAAAGAATAAAGCGATTTGATGTTCAAGGAATCACG

GAAGAAGATACTCATGATAAAGAAGCTCTAAAACTATTCAATAACCTTACAA

TGGAATTGATCGAAAGGGTGGAAGGTTAATGGTACGAAAATTAGGGGATCT

ACCTAGAAAGCCACAAGGCGATAGGTCAAGCTTAAAGAACCCTTACATGG

ATCTTACAGATTCTGAAAGTAAAGAAACAACAGAGGTTAAACAAACAGAA

CCAAAAAGAAAAAAAGCATTGTTGAAAACAATGAAAGTTGATGTTTCAAT

CCATAATAAGATTAAATCGCTGCACGAAATTCTGGCAGCATCCGAAGGGAA

TTCATATTACTTAGAGGATACTATTGAGAGAGCTATTGATAAGATGGTTGAG

ACATTACCTGAGAGCCAAAAAACTTTTTATGAATATGAATTAAAAAAAAGA

ACCAACAAAGGCTGAGACAGACTCCAAACGAGTCTGTTTTTTTAAAAAAA

ATATTAGGAGCATTGAATATATATTAGAGAATTAAGAAAGACATGGGAATAA

AAATATTTTAAATCCAGTAAAAATATGATAAGATTATTTCAGAATATGAAGAA

CTCTGTTTGTTTTTGATGAAAAAACAAACAAAAAAAATCCACCTAACGGAA

TCTCAATTTAACTAACAGCGGCCAAACTGAGAAGTTAAATTTGAGAAGGGG

AAAAGGCGGATTTATACTTGTATTTAACTATCTCCATTTTAACATTTTATTAA

ACCCCATACAAGTGAAAATCCTCTTTTACACTGTTCCTTTAGGTGATCGCGG

AGGGACATTATGAGTGAAGTAAACCTAAAAGGAAATACAGATGAATTAGTG

TATTATCGACAGCAAACCACTGGAAATAAAATCGCCAGGAAGAGAATCAAA

AAAGGGAAAGAAGAAGTTTATTATGTTGCTGAAACGGAAGAGAAGATATG

GACAGAAGAGCAAATAAAAAACTTTTCTTTAGACAAATTTGGTACGCATAT

ACCTTACATAGAAGGTCATTATACAATCTTAAATAATTACTTCTTTGATTTTT

GGGGCTATTTTTTAGGTGCTGAAGGAATTGCGCTCTATGCTCACCTAACTCG

TTATGCATACGGCAGCAAAGACTTTTGCTTTCCTAGTCTACAAACAATCGCT

AAAAAAATGGACAAGACTCCTGTTACAGTTAGAGGCTACTTGAAACTGCTT

GAAAGGTACGGTTTTATTTGGAAGGTAAACGTCCGTAATAAAACCAAGGAT

AACACAGAGGAATCCCCGATTTTTAAGATTAGACGTAAGGTTCCTTTGCTTT

CAGAAGAACTTTTAAATGGAAACCCTAATATTGAAATTCCAGATGACGAGG

AAGCACATGTAAAGAAGGCTTTAAAAAAGGAAAAAGAGGGTCTTCCAAA

GGTTTTGAAAAAAGAGCACGATGAATTTGTTAAAAAAATGATGGATGAGTC

AGAAACAATTAATATTCCAGAGGCCTTACAATATGACACAATGTATGAAGAT

ATACTCAGTAAAGGAGAAATTCGAAAAGAAATCAAAAAACAAATACCTAAT

CCTACAACATCTTTTGAGAGTATATCAATGACAACTGAAGAGGAAAAAGTC

GACAGTACTTTAAAAAGCGAAATGCAAAATCGTGTCTCTAAGCCTTCTTTT

GATACCTGGTTTAAAAACACTAAGATCAAAATTGAAAATAAAAATTGTTTAT

TACTTGTACCGAGTGAATTTGCATTTGAATGGATTAAGAAAAGATATTTAGA

AACAATTAAAACAGTCCTTGAAGAAGCTGGATATGTTTTCGAAAAAATCGA

ACTAAGAAAAGTGCAATAAACTGCTGAAGTATTTCAGCAGTTTTTTTTATTT

AGAAATAGTGAAAAAAATATAATCAGGGAGGTATCAATATTTAATGAGTACT

GATTTAAATTTATTTAGACTGGAATTAATAATTAACACGTAGACTAATTAAAA

TTTAATGAGGGATAAAGAGGATACAAAAATATTAATTTCAATCCCTATTAAAT

TTTAACAAGGGGGGGATTAAAATTTAATTAGAGGTTTATCCACAAGAAAAG

ACCCTAATAAAATTTTTACTAGGGTTATAACACTGATTAATTTCTTAATGGGG

GAGGGATTAAAATTTAATGACAAAGAAAACAATCTTTTAAGAAAAGCTTTT

AAAAGATAATAATAAAAAGAGCTTTGCGATTAAGCAAAACTCTTTACTTTTT

CATTGACATTATCAAATTCATCGATTTCAAATTGTTGTTGTATCATAAAGTTA

ATTCTGTTTTGCACAACCTTTTCAGGAATATAAAACACATCTGAGGCTTGTT

TTATAAACTCAGGGTCGCTAAAGTCAATGTAACGTAGCATATGATATGGTAT

AGCTTCCACCCAAGTTAGCCTTTCTGCTTCTTCTGAATGTTTTTCATATACTT

CCATGGGTATCTCTAAATGATTTTCCTCATGTAGCAAGGTATGAGCAAAAAG

TTTATGGAATTGATAGTTCCTCTCTTTTTCTTCAACTTTTTTATCTAAAACAA

ACACTTTAACATCTGAGTCAATGTAAGCATAAGATGTTTTTCCAGTCATAAT

TTCAATCCCAAATCTTTTAGACAGAAATTCTGGACGTAAATCTTTTGGTGAA

AGAATTTTTTTATGTAGCAATATATCCGATACAGCACCTTCTAAAAGCGTTG

GTGAATAGGGCATTTTACCTATCTCCTCTCATTTTGTGGAATAAAAATAGTCA

TATTCGTCCATCTACCTATCCTATTATCGAACAGTTGAACTTTTTAATCAAGG

ATCAGTCCTTTTTTTCATTATTCTTAAACTGTGCTCTTAACTTTAACAACTCG

ATTTGTTTTTCCAGATCTCGAGGGTAACTAGCCTCGCCGATCCCGCAAGAG

GCCCGGCAGTCAGGTGGCACTTTTCGGGGAAATGTGCGCGGAACCCCTATT

TGTTTATTTTTCTAAATACATTCAAATATGTATCCGCTCATGAGACAATAACC

CTGATAAATGCTTCAATAATATTGAAAAAGGAAGAGTATGAGTATTCAACAT

TTCCGTGTCGCCCTTATTCCCTTTTTTGCGGCATTTTGCCTTCCTGTTTTTGC

TCACCCAGAAACGCTGGTGAAAGTAAAAGATGCTGAAGATCAGTTGGGTG

CACGAGTGGGTTACATCGAACTGGATCTCAACAGCGGTAAGATCCTTGAGA

GTTTTCGCCCCGAAGAACGTTTTCCAATGATGAGCACTTTTAAAGTTCTGCT

ATGTGGCGCGGTATTATCCCGTATTGACGCCGGGCAAGAGCAACTCGGTCG

CCGCATACACTATTCTCAGAATGACTTGGTTGAGTACTCACCAGTCACAGA

AAAGCATCTTACGGATGGCATGACAGTAAGAGAATTATGCAGTGCTGCCAT

AACCATGAGTGATAACACTGCGGCCAACTTACTTCTGACAACGATCGGAGG

ACCGAAGGAGCTAACCGCTTTTTTGCACAACATGGGGGATCATGTAACTCG

CCTTGATCGTTGGGAACCGGAGCTGAATGAAGCCATACCAAACGACGAGC

GTGACACCACGATGCCTGTAGCAATGGCAACAACGTTGCGCAAACTATTAA

CTGGCGAACTACTTACTCTAGCTTCCCGGCAACAATTAATAGACTGGATGG

AGGCGGATAAAGTTGCAGGACCACTTCTGCGCTCGGCCCTTCCGGCTGGCT

GGTTTATTGCTGATAAATCTGGAGCCGGTGAGCGTGGGTCTCGCGGTATCAT

TGCAGCACTGGGGCCAGATGGTAAGCCCTCCCGTATCGTAGTTATCTACAC

GACGGGGAGTCAGGCAACTATGGATGAACGAAATAGACAGATCGCTGAGA

TAGGTGCCTCACTGATTAAGCATTGGTAACTGTCAGACCAAGTTTACTCATA

TATACTTTAGATTGATTTAAAACTTCATTTTTAATTTAAAAGGATCTAGGTGA

AGATCCTTTTTGATAATCTCATGACCAAAATCCCTTAACGTGAGTTTTCGTT

CCACTGAGCGTCAGACCCCGTAGAAAAGATCAAAGGATCTTCTTGAGATCC

TTTTTTTCTGCGCGTAATCTGCTGCTTGCAAACAAAAAAACCACCGCTACC

AGCGGTGGTTTGTTTGCCGGATCAAGAGCTACCAACTCTTTTTCCGAAGGT

AACTGGCTTCAGCAGAGCGCAGATACCAAATACTGTCCTTCTAGTGTAGCC

GTAGTTAGGCCACCACTTCAAGAACTCTGTAGCACCGCCTACATACCTCGC

TCTGCTAATCCTGTTACCAGTGGCTGCTGCCAGTGGCGATAAGTCGTGTCTT

ACCGGGTTGGACTCAAGACGATAGTTACCGGATAAGGCGCAGCGGTCGGG

CTGAACGGGGGGTTCGTGCACACAGCCCAGCTTGGAGCGAACGACCTACA

CCGAACTGAGATACCTACAGCGTGAGCTATGAGAAAGCGCCACGCTTCCCG

AAGGGAGAAAGGCGGACAGGTATCCGGTAAGCGGCAGGGTCGGAACAGG

AGAGCGCACGAGGGAGCTTCCAGGGGGAAACGCCTGGTATCTTTATAGTCC

TGTCGGGTTTCGCCACCTCTGACTTGAGCGTCGATTTTTGTGATGCTCGTCA

GGGGGGCGGAGCCTATGGAAAAACGCCAGCAACGCGGCCTTTTTACGGTT

CCTGGCCTTTTGCTGGCCTTTTGCTCACATGTTCTTTCCTGCGTTATCCCCTG

ATTCTGTGGATAACCGTATTACCGCCTTTGAGTGAGCTGATACCGCTCGCCG

CAGCCGAACGACCGAGCGCAGCGAGTCAGTGAGCGAGGAAGCGGAAGAG

CGCCCAATACGCATGC

pHT43-PBB-1:

TTAAGTTATTGGTATGACTGGTTTTAAGCGCAAAAAAAGTTGCTTTTTCGTA

CCTATTAATGTATCGTTTTAGAAAACCGACTGTAAAAAGTACAGTCGGCATT

ATCTCATATTATAAAAGCCAGTCATTAGGCCTATCTGACAATTCCTGAATAGA

GTTCATAAACAATCCTGCATGATAACCATCACAAACAGAATGATGTACCTGT

AAAGATAGCGGTAAATATATTGAATTACCTTTATTAATGAATTTTCCTGCTGT

AATAATGGGTAGAAGGTAATTACTATTATTATTGATATTTAAGTTAAACCCAG

TAAATGAAGTCCATGGAATAATAGAAAGAGAAAAAGCATTTTCAGGTATAG

GTGTTTTGGGAAACAATTTCCCCGAACCATTATATTTCTCTACATCAGAAAG

GTATAAATCATAAAACTCTTTGAAGTCATTCTTTACAGGAGTCCAAATACCA

GAGAATGTTTTAGATACACCATCAAAAATTGTATAAAGTGGCTCTAACTTAT

CCCAATAACCTAACTCTCCGTCGCTATTGTAACCAGTTCTAAAAGCTGTATT

TGAGTTTATCACCCTTGTCACTAAGAAAATAAATGCAGGGTAAAATTTATAT

CCTTCTTGTTTTATGTTTCGGTATAAAACACTAATATCAATTTCTGTGGTTATA

CTAAAAGTCGTTTGTTGGTTCAAATAATGATTAAATATCTCTTTTCTCTTCCA

ATTGTCTAAATCAATTTTATTAAAGTTCATTTGATATGCCTCCTAAATTTTTAT

CTAAAGTGAATTTAGGAGGCTTACTTGTCTGCTTTCTTCATTAGAATCAATC

CTTTTTTAAAAGTCAATATTACTGTAACATAAATATATATTTTAAAAATATCCC

ACTTTATCCAATTTTCGTTTGTTGAACTAATGGGTGCTTTAGTTGAAGAATA

AAGACCACATTAAAAAATGTGGTCTTTTGTGTTTTTTTAAAGGATTTGAGCG

TAGCGAAAAATCCTTTTCTTTCTTATCTTGATAATAAGGGTAACTATTGCCGA

TCGTCCATTCCGACAGCATCGCCAGTCACTATGGCGTGCTGCTAGCGCCATT

CGCCATTCAGGCTGCGCAACTGTTGGGAAGGGCGATCGGTGCGGGCCTCT

TCGCTATTACGCCAGCTGGCGAAAGGGGGATGTGCTGCAAGGCGATTAAGT

TGGGTAACGCCAGGGTTTTCCCAGTCACGACGTTGTAAAACGACGGCCAG

TGAATTCGAGCTCAGGCCTTAACTCACATTAATTGCGTTGCGCTCACTGCCC

GCTTTCCAGTCGGGAAACCTGTCGTGCCAGCTGCATTAATGAATCGGCCAA

CGCGCGGGGAGAGGCGGTTTGCGTATTGGGCGCCAGGGTGGTTTTTCTTTT

CACCAGTGAGACGGGCAACAGCTGATTGCCCTTCACCGCCTGGCCCTGAG

AGAGTTGCAGCAAGCGGTCCACGCTGGTTTGCCCCAGCAGGCGAAAATCC

TGTTTGATGGTGGTTGACGGCGGGATATAACATGAGCTGTCTTCGGTATCGT

CGTATCCCACTACCGAGATATCCGCACCAACGCGCAGCCCGGACTCGGTAA

TGGCGCGCATTGCGCCCAGCGCCATCTGATCGTTGGCAACCAGCATCGCAG

TGGGAACGATGCCCTCATTCAGCATTTGCATGGTTTGTTGAAAACCGGACA

TGGCACTCCAGTCGCCTTCCCGTTCCGCTATCGGCTGAATTTGATTGCGAGT

GAGATATTTATGCCAGCCAGCCAGACGCAGACGCGCCGAGACAGAACTTAA

TGGGCCCGCTAACAGCGCGATTTGCTGGTGACCCAATGCGACCAGATGCTC

CACGCCCAGTCGCGTACCGTCTTCATGGGAGAAAATAATACTGTTGATGGG

TGTCTGGTCAGAGACATCAAGAAATAACGCCGGAACATTAGTGCAGGCAG

CTTCCACAGCAATGGCATCCTGGTCATCCAGCGGATAGTTAATGATCAGCCC

ACTGACGCGTTGCGCGAGAAGATTGTGCACCGCCGCTTTACAGGCTTCGAC

GCCGCTTCGTTCTACCATCGACACCACCACGCTGGCACCCAGTTGATCGGC

GCGAGATTTAATCGCCGCGACAATTTGCGACGGCGCGTGCAGGGCCAGAC

TGGAGGTGGCAACGCCAATCAGCAACGACTGTTTGCCCGCCAGTTGTTGT

GCCACGCGGTTGGGAATGTAATTCAGCTCCGCCATCGCCGCTTCCACTTTTT

CCCGCGTTTTCGCAGAAACGTGGCTGGCCTGGTTCACCACGCGGGAAACG

GTCTGATAAGAGACACCGGCATACTCTGCGACATCGTATAACGTTACTGGTT

TCATCAAAATCGTCTCCCTCCGTTTGAATATTTGATTGATCGTAACCAGATG

AAGCACTCTTTCCACTATCCCTACAGTGTTATGGCTTGAACAATCACGAAAC

AATAATTGGTACGTACGATCTTTCAGCCGACTCAAACATCAAATCTTACAAA

TGTAGTCTTTGAAAGTATTACATATGTAAGATTTAAATGCAACCGTTTTTTCG

GAAGGAAATGATGACCTCGTTTCCACCGGAATTAGCTTGGTACCAGCTATT

GTAACATAATCGGTACGGGGGTGAAAAAGCTAACGGAAAAGGGAGCGGAA

AAGAATGATGTAAGCGTGAAAAATTTTTTATCTTATCACTTGAAATTGGAAG

GGAGATTCTTTATTATAAGAATTGTGGAATTGTGAGCGGATAACAATTCCCA

ATTAAAGGAGGAAGGATCAATGAACGAGAGACTTATCAAGATCGATGGAAT

TGAAATCTGCACAGAATCATTCGGAAAGCAAGTTAATCCGGCGATTCTTCTT

ATTATGGGAGCGCAGAGCAGCATGATTTGGTGGGAAGAAGAATTTTGCAGA

AGACTGGCAGATGCAGGAAGATATGTTATTAGATACGACAACCGCGATGTG

GGCAGAAGCACAACATATGAACTTGGCCAGCCGGGATATACATTTGAAGAT

ATGGCAGATGACGCGATTAGAGTGCTTGATGCATATGAAATTGAGCAAGCA

CATATCGTTGGAATGAGCATGGGCGGAATGCTGACACAGATTATTGCGCTG

AGACATCCGGGAAGAGTGAGAACAATTACACTTCTTAGCACATCAAACTTC

GCGCCGGATCTGCCGCTGATGGAAGAAAGAATTATGGATTATTTCAGCAAC

GTGGGAGCAATTGATTGGACAAATGAACAGGCGGTGGTTGAATTTGCAATT

GGCAGAAGCAGAATTCTGGTTGGATCAAAACATAGCTTCGATGAAAAGAG

AATCTACAACCTGGCAAAAGAAGAAGTTAAGAGAAGCCATAACATGGCGA

GCATGAATAATCATGGCATGCTTGTTGGAGGAGAATCATATCTTGTGAGAAC

AGGCGAAATTAAGGTTCCGGCACTTGTGATTCATGGCACAGAAGATCCGAT

TATTCCGTATGAACATGGCAAAAATCTGGTTAATGAGATCAGCGCGGCGGT

GCTTCTGACACTGGAAGGAACAGGCCATGAACTGCATTATGATGATTGGGA

TCTTATCATCGACTCAATTTCAAACCACACATCAATCCCGAATGAAAATGAA

TTCAGCAACTAAGGATCCTCTAGAGTCGACGTCCCCGGGGCAGCCCGCCTA

ATGAGCGGGCTTTTTTCACGTCACGCGTCCATGGAGATCTTTGTCTGCAAC

TGAAAAGTTTATACCTTACCTGGAACAAATGGTTGAAACATACGAGGCTAA

TATCGGCTTATTAGGAATAGTCCCTGTACTAATAAAATCAGGTGGATCAGTT

GATCAGTATATTTTGGACGAAGCTCGGAAAGAATTTGGAGATGACTTGCTT

AATTCCACAATTAAATTAAGGGAAAGAATAAAGCGATTTGATGTTCAAGGA

ATCACGGAAGAAGATACTCATGATAAAGAAGCTCTAAAACTATTCAATAAC

CTTACAATGGAATTGATCGAAAGGGTGGAAGGTTAATGGTACGAAAATTAG

GGGATCTACCTAGAAAGCCACAAGGCGATAGGTCAAGCTTAAAGAACCCTT

ACATGGATCTTACAGATTCTGAAAGTAAAGAAACAACAGAGGTTAAACAA

ACAGAACCAAAAAGAAAAAAAGCATTGTTGAAAACAATGAAAGTTGATGT

TTCAATCCATAATAAGATTAAATCGCTGCACGAAATTCTGGCAGCATCCGAA

GGGAATTCATATTACTTAGAGGATACTATTGAGAGAGCTATTGATAAGATGG

TTGAGACATTACCTGAGAGCCAAAAAACTTTTTATGAATATGAATTAAAAA

AAAGAACCAACAAAGGCTGAGACAGACTCCAAACGAGTCTGTTTTTTTAA

AAAAAATATTAGGAGCATTGAATATATATTAGAGAATTAAGAAAGACATGGG

AATAAAAATATTTTAAATCCAGTAAAAATATGATAAGATTATTTCAGAATATG

AAGAACTCTGTTTGTTTTTGATGAAAAAACAAACAAAAAAAATCCACCTA

ACGGAATCTCAATTTAACTAACAGCGGCCAAACTGAGAAGTTAAATTTGAG

AAGGGGAAAAGGCGGATTTATACTTGTATTTAACTATCTCCATTTTAACATTT

TATTAAACCCCATACAAGTGAAAATCCTCTTTTACACTGTTCCTTTAGGTGA

TCGCGGAGGGACATTATGAGTGAAGTAAACCTAAAAGGAAATACAGATGA

ATTAGTGTATTATCGACAGCAAACCACTGGAAATAAAATCGCCAGGAAGAG

AATCAAAAAAGGGAAAGAAGAAGTTTATTATGTTGCTGAAACGGAAGAGA

AGATATGGACAGAAGAGCAAATAAAAAACTTTTCTTTAGACAAATTTGGTA

CGCATATACCTTACATAGAAGGTCATTATACAATCTTAAATAATTACTTCTTT

GATTTTTGGGGCTATTTTTTAGGTGCTGAAGGAATTGCGCTCTATGCTCACC

TAACTCGTTATGCATACGGCAGCAAAGACTTTTGCTTTCCTAGTCTACAAAC

AATCGCTAAAAAAATGGACAAGACTCCTGTTACAGTTAGAGGCTACTTGAA

ACTGCTTGAAAGGTACGGTTTTATTTGGAAGGTAAACGTCCGTAATAAAAC

CAAGGATAACACAGAGGAATCCCCGATTTTTAAGATTAGACGTAAGGTTCC

TTTGCTTTCAGAAGAACTTTTAAATGGAAACCCTAATATTGAAATTCCAGAT

GACGAGGAAGCACATGTAAAGAAGGCTTTAAAAAAGGAAAAAGAGGGTC

TTCCAAAGGTTTTGAAAAAAGAGCACGATGAATTTGTTAAAAAAATGATGG

ATGAGTCAGAAACAATTAATATTCCAGAGGCCTTACAATATGACACAATGTA

TGAAGATATACTCAGTAAAGGAGAAATTCGAAAAGAAATCAAAAAACAAA

TACCTAATCCTACAACATCTTTTGAGAGTATATCAATGACAACTGAAGAGGA

AAAAGTCGACAGTACTTTAAAAAGCGAAATGCAAAATCGTGTCTCTAAGCC

TTCTTTTGATACCTGGTTTAAAAACACTAAGATCAAAATTGAAAATAAAAAT

TGTTTATTACTTGTACCGAGTGAATTTGCATTTGAATGGATTAAGAAAAGAT

ATTTAGAAACAATTAAAACAGTCCTTGAAGAAGCTGGATATGTTTTCGAAA

AAATCGAACTAAGAAAAGTGCAATAAACTGCTGAAGTATTTCAGCAGTTTT

TTTTATTTAGAAATAGTGAAAAAAATATAATCAGGGAGGTATCAATATTTAAT

GAGTACTGATTTAAATTTATTTAGACTGGAATTAATAATTAACACGTAGACTA

ATTAAAATTTAATGAGGGATAAAGAGGATACAAAAATATTAATTTCAATCCC

TATTAAATTTTAACAAGGGGGGGATTAAAATTTAATTAGAGGTTTATCCACA

AGAAAAGACCCTAATAAAATTTTTACTAGGGTTATAACACTGATTAATTTCTT

AATGGGGGAGGGATTAAAATTTAATGACAAAGAAAACAATCTTTTAAGAAA

AGCTTTTAAAAGATAATAATAAAAAGAGCTTTGCGATTAAGCAAAACTCTTT

ACTTTTTCATTGACATTATCAAATTCATCGATTTCAAATTGTTGTTGTATCATA

AAGTTAATTCTGTTTTGCACAACCTTTTCAGGAATATAAAACACATCTGAGG

CTTGTTTTATAAACTCAGGGTCGCTAAAGTCAATGTAACGTAGCATATGATAT

GGTATAGCTTCCACCCAAGTTAGCCTTTCTGCTTCTTCTGAATGTTTTTCATA

TACTTCCATGGGTATCTCTAAATGATTTTCCTCATGTAGCAAGGTATGAGCA

AAAAGTTTATGGAATTGATAGTTCCTCTCTTTTTCTTCAACTTTTTTATCTAA

AACAAACACTTTAACATCTGAGTCAATGTAAGCATAAGATGTTTTTCCAGTC

ATAATTTCAATCCCAAATCTTTTAGACAGAAATTCTGGACGTAAATCTTTTG

GTGAAAGAATTTTTTTATGTAGCAATATATCCGATACAGCACCTTCTAAAAG

CGTTGGTGAATAGGGCATTTTACCTATCTCCTCTCATTTTGTGGAATAAAAAT

AGTCATATTCGTCCATCTACCTATCCTATTATCGAACAGTTGAACTTTTTAAT

CAAGGATCAGTCCTTTTTTTCATTATTCTTAAACTGTGCTCTTAACTTTAACA

ACTCGATTTGTTTTTCCAGATCTCGAGGGTAACTAGCCTCGCCGATCCCGCA

AGAGGCCCGGCAGTCAGGTGGCACTTTTCGGGGAAATGTGCGCGGAACCC

CTATTTGTTTATTTTTCTAAATACATTCAAATATGTATCCGCTCATGAGACAAT

AACCCTGATAAATGCTTCAATAATATTGAAAAAGGAAGAGTATGAGTATTCA

ACATTTCCGTGTCGCCCTTATTCCCTTTTTTGCGGCATTTTGCCTTCCTGTTT

TTGCTCACCCAGAAACGCTGGTGAAAGTAAAAGATGCTGAAGATCAGTTG

GGTGCACGAGTGGGTTACATCGAACTGGATCTCAACAGCGGTAAGATCCTT

GAGAGTTTTCGCCCCGAAGAACGTTTTCCAATGATGAGCACTTTTAAAGTT

CTGCTATGTGGCGCGGTATTATCCCGTATTGACGCCGGGCAAGAGCAACTC

GGTCGCCGCATACACTATTCTCAGAATGACTTGGTTGAGTACTCACCAGTC

ACAGAAAAGCATCTTACGGATGGCATGACAGTAAGAGAATTATGCAGTGCT

GCCATAACCATGAGTGATAACACTGCGGCCAACTTACTTCTGACAACGATC

GGAGGACCGAAGGAGCTAACCGCTTTTTTGCACAACATGGGGGATCATGTA

ACTCGCCTTGATCGTTGGGAACCGGAGCTGAATGAAGCCATACCAAACGA

CGAGCGTGACACCACGATGCCTGTAGCAATGGCAACAACGTTGCGCAAAC

TATTAACTGGCGAACTACTTACTCTAGCTTCCCGGCAACAATTAATAGACTG

GATGGAGGCGGATAAAGTTGCAGGACCACTTCTGCGCTCGGCCCTTCCGGC

TGGCTGGTTTATTGCTGATAAATCTGGAGCCGGTGAGCGTGGGTCTCGCGG

TATCATTGCAGCACTGGGGCCAGATGGTAAGCCCTCCCGTATCGTAGTTATC

TACACGACGGGGAGTCAGGCAACTATGGATGAACGAAATAGACAGATCGC

TGAGATAGGTGCCTCACTGATTAAGCATTGGTAACTGTCAGACCAAGTTTA

CTCATATATACTTTAGATTGATTTAAAACTTCATTTTTAATTTAAAAGGATCTA

GGTGAAGATCCTTTTTGATAATCTCATGACCAAAATCCCTTAACGTGAGTTT

TCGTTCCACTGAGCGTCAGACCCCGTAGAAAAGATCAAAGGATCTTCTTGA

GATCCTTTTTTTCTGCGCGTAATCTGCTGCTTGCAAACAAAAAAACCACCG

CTACCAGCGGTGGTTTGTTTGCCGGATCAAGAGCTACCAACTCTTTTTCCG

AAGGTAACTGGCTTCAGCAGAGCGCAGATACCAAATACTGTCCTTCTAGTG

TAGCCGTAGTTAGGCCACCACTTCAAGAACTCTGTAGCACCGCCTACATAC

CTCGCTCTGCTAATCCTGTTACCAGTGGCTGCTGCCAGTGGCGATAAGTCGT

GTCTTACCGGGTTGGACTCAAGACGATAGTTACCGGATAAGGCGCAGCGGT

CGGGCTGAACGGGGGGTTCGTGCACACAGCCCAGCTTGGAGCGAACGAC

CTACACCGAACTGAGATACCTACAGCGTGAGCTATGAGAAAGCGCCACGCT

TCCCGAAGGGAGAAAGGCGGACAGGTATCCGGTAAGCGGCAGGGTCGGA

ACAGGAGAGCGCACGAGGGAGCTTCCAGGGGGAAACGCCTGGTATCTTTA

TAGTCCTGTCGGGTTTCGCCACCTCTGACTTGAGCGTCGATTTTTGTGATGC

TCGTCAGGGGGGCGGAGCCTATGGAAAAACGCCAGCAACGCGGCCTTTTT

ACGGTTCCTGGCCTTTTGCTGGCCTTTTGCTCACATGTTCTTTCCTGCGTTAT

CCCCTGATTCTGTGGATAACCGTATTACCGCCTTTGAGTGAGCTGATACCGC

TCGCCGCAGCCGAACGACCGAGCGCAGCGAGTCAGTGAGCGAGGAAGCG

GAAGAGCGCCCAATACGCATGC

pHT43-CBF-1:

TTAAGTTATTGGTATGACTGGTTTTAAGCGCAAAAAAAGTTGCTTTTTCGTA

CCTATTAATGTATCGTTTTAGAAAACCGACTGTAAAAAGTACAGTCGGCATT

ATCTCATATTATAAAAGCCAGTCATTAGGCCTATCTGACAATTCCTGAATAGA

GTTCATAAACAATCCTGCATGATAACCATCACAAACAGAATGATGTACCTGT

AAAGATAGCGGTAAATATATTGAATTACCTTTATTAATGAATTTTCCTGCTGT

AATAATGGGTAGAAGGTAATTACTATTATTATTGATATTTAAGTTAAACCCAG

TAAATGAAGTCCATGGAATAATAGAAAGAGAAAAAGCATTTTCAGGTATAG

GTGTTTTGGGAAACAATTTCCCCGAACCATTATATTTCTCTACATCAGAAAG

GTATAAATCATAAAACTCTTTGAAGTCATTCTTTACAGGAGTCCAAATACCA

GAGAATGTTTTAGATACACCATCAAAAATTGTATAAAGTGGCTCTAACTTAT

CCCAATAACCTAACTCTCCGTCGCTATTGTAACCAGTTCTAAAAGCTGTATT

TGAGTTTATCACCCTTGTCACTAAGAAAATAAATGCAGGGTAAAATTTATAT

CCTTCTTGTTTTATGTTTCGGTATAAAACACTAATATCAATTTCTGTGGTTATA

CTAAAAGTCGTTTGTTGGTTCAAATAATGATTAAATATCTCTTTTCTCTTCCA

ATTGTCTAAATCAATTTTATTAAAGTTCATTTGATATGCCTCCTAAATTTTTAT

CTAAAGTGAATTTAGGAGGCTTACTTGTCTGCTTTCTTCATTAGAATCAATC

CTTTTTTAAAAGTCAATATTACTGTAACATAAATATATATTTTAAAAATATCCC

ACTTTATCCAATTTTCGTTTGTTGAACTAATGGGTGCTTTAGTTGAAGAATA

AAGACCACATTAAAAAATGTGGTCTTTTGTGTTTTTTTAAAGGATTTGAGCG

TAGCGAAAAATCCTTTTCTTTCTTATCTTGATAATAAGGGTAACTATTGCCGA

TCGTCCATTCCGACAGCATCGCCAGTCACTATGGCGTGCTGCTAGCGCCATT

CGCCATTCAGGCTGCGCAACTGTTGGGAAGGGCGATCGGTGCGGGCCTCT

TCGCTATTACGCCAGCTGGCGAAAGGGGGATGTGCTGCAAGGCGATTAAGT

TGGGTAACGCCAGGGTTTTCCCAGTCACGACGTTGTAAAACGACGGCCAG

TGAATTCGAGCTCAGGCCTTAACTCACATTAATTGCGTTGCGCTCACTGCCC

GCTTTCCAGTCGGGAAACCTGTCGTGCCAGCTGCATTAATGAATCGGCCAA

CGCGCGGGGAGAGGCGGTTTGCGTATTGGGCGCCAGGGTGGTTTTTCTTTT

CACCAGTGAGACGGGCAACAGCTGATTGCCCTTCACCGCCTGGCCCTGAG

AGAGTTGCAGCAAGCGGTCCACGCTGGTTTGCCCCAGCAGGCGAAAATCC

TGTTTGATGGTGGTTGACGGCGGGATATAACATGAGCTGTCTTCGGTATCGT

CGTATCCCACTACCGAGATATCCGCACCAACGCGCAGCCCGGACTCGGTAA

TGGCGCGCATTGCGCCCAGCGCCATCTGATCGTTGGCAACCAGCATCGCAG

TGGGAACGATGCCCTCATTCAGCATTTGCATGGTTTGTTGAAAACCGGACA

TGGCACTCCAGTCGCCTTCCCGTTCCGCTATCGGCTGAATTTGATTGCGAGT

GAGATATTTATGCCAGCCAGCCAGACGCAGACGCGCCGAGACAGAACTTAA

TGGGCCCGCTAACAGCGCGATTTGCTGGTGACCCAATGCGACCAGATGCTC

CACGCCCAGTCGCGTACCGTCTTCATGGGAGAAAATAATACTGTTGATGGG

TGTCTGGTCAGAGACATCAAGAAATAACGCCGGAACATTAGTGCAGGCAG

CTTCCACAGCAATGGCATCCTGGTCATCCAGCGGATAGTTAATGATCAGCCC

ACTGACGCGTTGCGCGAGAAGATTGTGCACCGCCGCTTTACAGGCTTCGAC

GCCGCTTCGTTCTACCATCGACACCACCACGCTGGCACCCAGTTGATCGGC

GCGAGATTTAATCGCCGCGACAATTTGCGACGGCGCGTGCAGGGCCAGAC

TGGAGGTGGCAACGCCAATCAGCAACGACTGTTTGCCCGCCAGTTGTTGT

GCCACGCGGTTGGGAATGTAATTCAGCTCCGCCATCGCCGCTTCCACTTTTT

CCCGCGTTTTCGCAGAAACGTGGCTGGCCTGGTTCACCACGCGGGAAACG

GTCTGATAAGAGACACCGGCATACTCTGCGACATCGTATAACGTTACTGGTT

TCATCAAAATCGTCTCCCTCCGTTTGAATATTTGATTGATCGTAACCAGATG

AAGCACTCTTTCCACTATCCCTACAGTGTTATGGCTTGAACAATCACGAAAC

AATAATTGGTACGTACGATCTTTCAGCCGACTCAAACATCAAATCTTACAAA

TGTAGTCTTTGAAAGTATTACATATGTAAGATTTAAATGCAACCGTTTTTTCG

GAAGGAAATGATGACCTCGTTTCCACCGGAATTAGCTTGGTACCAGCTATT

GTAACATAATCGGTACGGGGGTGAAAAAGCTAACGGAAAAGGGAGCGGAA

AAGAATGATGTAAGCGTGAAAAATTTTTTATCTTATCACTTGAAATTGGAAG

GGAGATTCTTTATTATAAGAATTGTGGAATTGTGAGCGGATAACAATTCCCA

ATTAAAGGAGGAAGGATCAATGCGTGAACAAATCATGAAGATCAACAAGG

TTAACATCTGCACAGAATCATTCGGAAATAGCGAAGATCCGGCAATTCTTCT

GATTATGGGAGCAATGACATCACTGGATTGGTGGGATGAAGATTTTTGCCTT

AGACTTGCAGATCAGGGAAGATTTGTTATTAGATACGACCATCGCGATCTTG

GAAGAAGCACAACATATGAACCGGGCACATCAAATTATACAATCACAGATC

TGGCGGATGATGCAGCAGGAGTGCTGGATGCATATCATATTGGACAAGCGC

ATATTGTGGGAATGAGCATGGGCGGACTGACAGGACAAATTCTTGCACTGA

GATATCCGGATAGAGTGCTGACACTGACACTGATTGCGAGCTCAGTTTTTG

GCACAGAAATGGAAAAACTGCCGCCGATGGATCAAAATATTCTGGATTATC

ACGCGAAGTCAGCAAGCATTGATTGGACAAATAGAGATGCGGCAATTCCGT

ATCTGGCAGGCGGCTGGAAAACACTTGCGGGATCAAAACCGTTTGAACAA

GAAAGAATCTACAAGCTTGCGGAAAGAGAAGCAGATAGAGCGAATCATCT

TCCGAGCAGATTCAATCATGCGCTGCTGCAGGGCGGCGATGTGTATTTTGAT

AGAATGAATGAGATCAGCGCGCCGGTGCTTATTATTCATGGAACAGAAGAT

CCGGCGCTTCCGTATGAACATGGACTGGCGCTTAAAAAAGCGATTCCGCAT

TCAGAACTGGTTACACTGGAAGGAACAGGACATGAAATTCATTCAGAAGA

TTGGAACCAGATCATCGATAGCGTTGTGAAACTTAGCAGCAGACTTGAAGA

ATAAGGATCCTCTAGAGTCGACGTCCCCGGGGCAGCCCGCCTAATGAGCGG

GCTTTTTTCACGTCACGCGTCCATGGAGATCTTTGTCTGCAACTGAAAAGT

TTATACCTTACCTGGAACAAATGGTTGAAACATACGAGGCTAATATCGGCTT

ATTAGGAATAGTCCCTGTACTAATAAAATCAGGTGGATCAGTTGATCAGTAT

ATTTTGGACGAAGCTCGGAAAGAATTTGGAGATGACTTGCTTAATTCCACA

ATTAAATTAAGGGAAAGAATAAAGCGATTTGATGTTCAAGGAATCACGGAA

GAAGATACTCATGATAAAGAAGCTCTAAAACTATTCAATAACCTTACAATGG

AATTGATCGAAAGGGTGGAAGGTTAATGGTACGAAAATTAGGGGATCTACC

TAGAAAGCCACAAGGCGATAGGTCAAGCTTAAAGAACCCTTACATGGATCT

TACAGATTCTGAAAGTAAAGAAACAACAGAGGTTAAACAAACAGAACCAA

AAAGAAAAAAAGCATTGTTGAAAACAATGAAAGTTGATGTTTCAATCCATA

ATAAGATTAAATCGCTGCACGAAATTCTGGCAGCATCCGAAGGGAATTCATA

TTACTTAGAGGATACTATTGAGAGAGCTATTGATAAGATGGTTGAGACATTA

CCTGAGAGCCAAAAAACTTTTTATGAATATGAATTAAAAAAAAGAACCAAC

AAAGGCTGAGACAGACTCCAAACGAGTCTGTTTTTTTAAAAAAAATATTAG

GAGCATTGAATATATATTAGAGAATTAAGAAAGACATGGGAATAAAAATATT

TTAAATCCAGTAAAAATATGATAAGATTATTTCAGAATATGAAGAACTCTGT

TTGTTTTTGATGAAAAAACAAACAAAAAAAATCCACCTAACGGAATCTCAA

TTTAACTAACAGCGGCCAAACTGAGAAGTTAAATTTGAGAAGGGGAAAAG

GCGGATTTATACTTGTATTTAACTATCTCCATTTTAACATTTTATTAAACCCCA

TACAAGTGAAAATCCTCTTTTACACTGTTCCTTTAGGTGATCGCGGAGGGA

CATTATGAGTGAAGTAAACCTAAAAGGAAATACAGATGAATTAGTGTATTAT

CGACAGCAAACCACTGGAAATAAAATCGCCAGGAAGAGAATCAAAAAAG

GGAAAGAAGAAGTTTATTATGTTGCTGAAACGGAAGAGAAGATATGGACA

GAAGAGCAAATAAAAAACTTTTCTTTAGACAAATTTGGTACGCATATACCTT

ACATAGAAGGTCATTATACAATCTTAAATAATTACTTCTTTGATTTTTGGGGC

TATTTTTTAGGTGCTGAAGGAATTGCGCTCTATGCTCACCTAACTCGTTATGC

ATACGGCAGCAAAGACTTTTGCTTTCCTAGTCTACAAACAATCGCTAAAAA

AATGGACAAGACTCCTGTTACAGTTAGAGGCTACTTGAAACTGCTTGAAAG

GTACGGTTTTATTTGGAAGGTAAACGTCCGTAATAAAACCAAGGATAACAC

AGAGGAATCCCCGATTTTTAAGATTAGACGTAAGGTTCCTTTGCTTTCAGAA

GAACTTTTAAATGGAAACCCTAATATTGAAATTCCAGATGACGAGGAAGCA

CATGTAAAGAAGGCTTTAAAAAAGGAAAAAGAGGGTCTTCCAAAGGTTTT

GAAAAAAGAGCACGATGAATTTGTTAAAAAAATGATGGATGAGTCAGAAA

CAATTAATATTCCAGAGGCCTTACAATATGACACAATGTATGAAGATATACTC

AGTAAAGGAGAAATTCGAAAAGAAATCAAAAAACAAATACCTAATCCTAC

AACATCTTTTGAGAGTATATCAATGACAACTGAAGAGGAAAAAGTCGACA

GTACTTTAAAAAGCGAAATGCAAAATCGTGTCTCTAAGCCTTCTTTTGATAC

CTGGTTTAAAAACACTAAGATCAAAATTGAAAATAAAAATTGTTTATTACTT

GTACCGAGTGAATTTGCATTTGAATGGATTAAGAAAAGATATTTAGAAACAA

TTAAAACAGTCCTTGAAGAAGCTGGATATGTTTTCGAAAAAATCGAACTAA

GAAAAGTGCAATAAACTGCTGAAGTATTTCAGCAGTTTTTTTATTTAGAAA

TAGTGAAAAAAATATAATCAGGGAGGTATCAATATTTAATGAGTACTGATTT

AAATTTATTTAGACTGGAATTAATAATTAACACGTAGACTAATTAAAATTTAA

TGAGGGATAAAGAGGATACAAAAATATTAATTTCAATCCCTATTAAATTTTA

ACAAGGGGGGGATTAAAATTTAATTAGAGGTTTATCCACAAGAAAAGACCC

TAATAAAATTTTTACTAGGGTTATAACACTGATTAATTTCTTAATGGGGGAGG

GATTAAAATTTAATGACAAAGAAAACAATCTTTTAAGAAAAGCTTTTAAAA

GATAATAATAAAAAGAGCTTTGCGATTAAGCAAAACTCTTTACTTTTTCATT

GACATTATCAAATTCATCGATTTCAAATTGTTGTTGTATCATAAAGTTAATTC

TGTTTTGCACAACCTTTTCAGGAATATAAAACACATCTGAGGCTTGTTTTAT

AAACTCAGGGTCGCTAAAGTCAATGTAACGTAGCATATGATATGGTATAGCT

TCCACCCAAGTTAGCCTTTCTGCTTCTTCTGAATGTTTTTCATATACTTCCAT

GGGTATCTCTAAATGATTTTCCTCATGTAGCAAGGTATGAGCAAAAAGTTTA

TGGAATTGATAGTTCCTCTCTTTTTCTTCAACTTTTTTATCTAAAACAAACAC

TTTAACATCTGAGTCAATGTAAGCATAAGATGTTTTTCCAGTCATAATTTCAA

TCCCAAATCTTTTAGACAGAAATTCTGGACGTAAATCTTTTGGTGAAAGAA

TTTTTTTATGTAGCAATATATCCGATACAGCACCTTCTAAAAGCGTTGGTGAA

TAGGGCATTTTACCTATCTCCTCTCATTTTGTGGAATAAAAATAGTCATATTC

GTCCATCTACCTATCCTATTATCGAACAGTTGAACTTTTTAATCAAGGATCAG

TCCTTTTTTTCATTATTCTTAAACTGTGCTCTTAACTTTAACAACTCGATTTG

TTTTTCCAGATCTCGAGGGTAACTAGCCTCGCCGATCCCGCAAGAGGCCCG

GCAGTCAGGTGGCACTTTTCGGGGAAATGTGCGCGGAACCCCTATTTGTTT

ATTTTTCTAAATACATTCAAATATGTATCCGCTCATGAGACAATAACCCTGAT

AAATGCTTCAATAATATTGAAAAAGGAAGAGTATGAGTATTCAACATTTCCG

TGTCGCCCTTATTCCCTTTTTTGCGGCATTTTGCCTTCCTGTTTTTGCTCACC

CAGAAACGCTGGTGAAAGTAAAAGATGCTGAAGATCAGTTGGGTGCACGA

GTGGGTTACATCGAACTGGATCTCAACAGCGGTAAGATCCTTGAGAGTTTT

CGCCCCGAAGAACGTTTTCCAATGATGAGCACTTTTAAAGTTCTGCTATGT

GGCGCGGTATTATCCCGTATTGACGCCGGGCAAGAGCAACTCGGTCGCCGC

ATACACTATTCTCAGAATGACTTGGTTGAGTACTCACCAGTCACAGAAAAG

CATCTTACGGATGGCATGACAGTAAGAGAATTATGCAGTGCTGCCATAACCA

TGAGTGATAACACTGCGGCCAACTTACTTCTGACAACGATCGGAGGACCG

AAGGAGCTAACCGCTTTTTTGCACAACATGGGGGATCATGTAACTCGCCTT

GATCGTTGGGAACCGGAGCTGAATGAAGCCATACCAAACGACGAGCGTGA

CACCACGATGCCTGTAGCAATGGCAACAACGTTGCGCAAACTATTAACTGG

CGAACTACTTACTCTAGCTTCCCGGCAACAATTAATAGACTGGATGGAGGC

GGATAAAGTTGCAGGACCACTTCTGCGCTCGGCCCTTCCGGCTGGCTGGTT

TATTGCTGATAAATCTGGAGCCGGTGAGCGTGGGTCTCGCGGTATCATTGCA

GCACTGGGGCCAGATGGTAAGCCCTCCCGTATCGTAGTTATCTACACGACG

GGGAGTCAGGCAACTATGGATGAACGAAATAGACAGATCGCTGAGATAGG

TGCCTCACTGATTAAGCATTGGTAACTGTCAGACCAAGTTTACTCATATATA

CTTTAGATTGATTTAAAACTTCATTTTTAATTTAAAAGGATCTAGGTGAAGAT

CCTTTTTGATAATCTCATGACCAAAATCCCTTAACGTGAGTTTTCGTTCCAC

TGAGCGTCAGACCCCGTAGAAAAGATCAAAGGATCTTCTTGAGATCCTTTT

TTTCTGCGCGTAATCTGCTGCTTGCAAACAAAAAAACCACCGCTACCAGCG

GTGGTTTGTTTGCCGGATCAAGAGCTACCAACTCTTTTTCCGAAGGTAACT

GGCTTCAGCAGAGCGCAGATACCAAATACTGTCCTTCTAGTGTAGCCGTAG

TTAGGCCACCACTTCAAGAACTCTGTAGCACCGCCTACATACCTCGCTCTG

CTAATCCTGTTACCAGTGGCTGCTGCCAGTGGCGATAAGTCGTGTCTTACCG

GGTTGGACTCAAGACGATAGTTACCGGATAAGGCGCAGCGGTCGGGCTGA

ACGGGGGGTTCGTGCACACAGCCCAGCTTGGAGCGAACGACCTACACCGA

ACTGAGATACCTACAGCGTGAGCTATGAGAAAGCGCCACGCTTCCCGAAG

GGAGAAAGGCGGACAGGTATCCGGTAAGCGGCAGGGTCGGAACAGGAGA

GCGCACGAGGGAGCTTCCAGGGGGAAACGCCTGGTATCTTTATAGTCCTGT

CGGGTTTCGCCACCTCTGACTTGAGCGTCGATTTTTGTGATGCTCGTCAGG

GGGGCGGAGCCTATGGAAAAACGCCAGCAACGCGGCCTTTTTACGGTTCC

TGGCCTTTTGCTGGCCTTTTGCTCACATGTTCTTTCCTGCGTTATCCCCTGAT

TCTGTGGATAACCGTATTACCGCCTTTGAGTGAGCTGATACCGCTCGCCGCA

GCCGAACGACCGAGCGCAGCGAGTCAGTGAGCGAGGAAGCGGAAGAGCG

CCCAATACGCATGC

pHT43-PBA-1:

TTAAGTTATTGGTATGACTGGTTTTAAGCGCAAAAAAAGTTGCTTTTTCGTA

CCTATTAATGTATCGTTTTAGAAAACCGACTGTAAAAAGTACAGTCGGCATT

ATCTCATATTATAAAAGCCAGTCATTAGGCCTATCTGACAATTCCTGAATAGA

GTTCATAAACAATCCTGCATGATAACCATCACAAACAGAATGATGTACCTGT

AAAGATAGCGGTAAATATATTGAATTACCTTTATTAATGAATTTTCCTGCTGT

AATAATGGGTAGAAGGTAATTACTATTATTATTGATATTTAAGTTAAACCCAG

TAAATGAAGTCCATGGAATAATAGAAAGAGAAAAAGCATTTTCAGGTATAG

GTGTTTTGGGAAACAATTTCCCCGAACCATTATATTTCTCTACATCAGAAAG

GTATAAATCATAAAACTCTTTGAAGTCATTCTTTACAGGAGTCCAAATACCA

GAGAATGTTTTAGATACACCATCAAAAATTGTATAAAGTGGCTCTAACTTAT

CCCAATAACCTAACTCTCCGTCGCTATTGTAACCAGTTCTAAAAGCTGTATT

TGAGTTTATCACCCTTGTCACTAAGAAAATAAATGCAGGGTAAAATTTATAT

CCTTCTTGTTTTATGTTTCGGTATAAAACACTAATATCAATTTCTGTGGTTATA

CTAAAAGTCGTTTGTTGGTTCAAATAATGATTAAATATCTCTTTTCTCTTCCA

ATTGTCTAAATCAATTTTATTAAAGTTCATTTGATATGCCTCCTAAATTTTTAT

CTAAAGTGAATTTAGGAGGCTTACTTGTCTGCTTTCTTCATTAGAATCAATC

CTTTTTTAAAAGTCAATATTACTGTAACATAAATATATATTTTAAAAATATCCC

ACTTTATCCAATTTTCGTTTGTTGAACTAATGGGTGCTTTAGTTGAAGAATA

AAGACCACATTAAAAAATGTGGTCTTTTGTGTTTTTTTAAAGGATTTGAGCG

TAGCGAAAAATCCTTTTCTTTCTTATCTTGATAATAAGGGTAACTATTGCCGA

TCGTCCATTCCGACAGCATCGCCAGTCACTATGGCGTGCTGCTAGCGCCATT

CGCCATTCAGGCTGCGCAACTGTTGGGAAGGGCGATCGGTGCGGGCCTCT

TCGCTATTACGCCAGCTGGCGAAAGGGGGATGTGCTGCAAGGCGATTAAGT

TGGGTAACGCCAGGGTTTTCCCAGTCACGACGTTGTAAAACGACGGCCAG

TGAATTCGAGCTCAGGCCTTAACTCACATTAATTGCGTTGCGCTCACTGCCC

GCTTTCCAGTCGGGAAACCTGTCGTGCCAGCTGCATTAATGAATCGGCCAA

CGCGCGGGGAGAGGCGGTTTGCGTATTGGGCGCCAGGGTGGTTTTTCTTTT

CACCAGTGAGACGGGCAACAGCTGATTGCCCTTCACCGCCTGGCCCTGAG

AGAGTTGCAGCAAGCGGTCCACGCTGGTTTGCCCCAGCAGGCGAAAATCC

TGTTTGATGGTGGTTGACGGCGGGATATAACATGAGCTGTCTTCGGTATCGT

CGTATCCCACTACCGAGATATCCGCACCAACGCGCAGCCCGGACTCGGTAA

TGGCGCGCATTGCGCCCAGCGCCATCTGATCGTTGGCAACCAGCATCGCAG

TGGGAACGATGCCCTCATTCAGCATTTGCATGGTTTGTTGAAAACCGGACA

TGGCACTCCAGTCGCCTTCCCGTTCCGCTATCGGCTGAATTTGATTGCGAGT

GAGATATTTATGCCAGCCAGCCAGACGCAGACGCGCCGAGACAGAACTTAA

TGGGCCCGCTAACAGCGCGATTTGCTGGTGACCCAATGCGACCAGATGCTC

CACGCCCAGTCGCGTACCGTCTTCATGGGAGAAAATAATACTGTTGATGGG

TGTCTGGTCAGAGACATCAAGAAATAACGCCGGAACATTAGTGCAGGCAG

CTTCCACAGCAATGGCATCCTGGTCATCCAGCGGATAGTTAATGATCAGCCC

ACTGACGCGTTGCGCGAGAAGATTGTGCACCGCCGCTTTACAGGCTTCGAC

GCCGCTTCGTTCTACCATCGACACCACCACGCTGGCACCCAGTTGATCGGC

GCGAGATTTAATCGCCGCGACAATTTGCGACGGCGCGTGCAGGGCCAGAC

TGGAGGTGGCAACGCCAATCAGCAACGACTGTTTGCCCGCCAGTTGTTGT

GCCACGCGGTTGGGAATGTAATTCAGCTCCGCCATCGCCGCTTCCACTTTTT

CCCGCGTTTTCGCAGAAACGTGGCTGGCCTGGTTCACCACGCGGGAAACG

GTCTGATAAGAGACACCGGCATACTCTGCGACATCGTATAACGTTACTGGTT

TCATCAAAATCGTCTCCCTCCGTTTGAATATTTGATTGATCGTAACCAGATG

AAGCACTCTTTCCACTATCCCTACAGTGTTATGGCTTGAACAATCACGAAAC

AATAATTGGTACGTACGATCTTTCAGCCGACTCAAACATCAAATCTTACAAA

TGTAGTCTTTGAAAGTATTACATATGTAAGATTTAAATGCAACCGTTTTTTCG

GAAGGAAATGATGACCTCGTTTCCACCGGAATTAGCTTGGTACCAGCTATT

GTAACATAATCGGTACGGGGGTGAAAAAGCTAACGGAAAAGGGAGCGGAA

AAGAATGATGTAAGCGTGAAAAATTTTTTATCTTATCACTTGAAATTGGAAG

GGAGATTCTTTATTATAAGAATTGTGGAATTGTGAGCGGATAACAATTCCCA

ATTAAAGGAGGAAGGATCAATGAGCGAAAAGCTGATTAAGATCAACGGAA

TTGACAACGGCATTGATATTTGCACAGAATCATTCGGCAACCCGAATAATCC

GGCAATTCTTCTGATTATGGGAGCAATGTGTTCAATGGTTTACTGGGATGAA

GAATTCTGTCAGAGACTTGCAGATACAGGCAGATATGTTATTAGATACGACA

ACAGAGACGTCGGCAGATCAATTGCATATGAACCGGGAAATAGCCAATATA

CAGTGGAAGATATGGCAGATGATGCAATTGGAGTGCTTGATGCATATTCAAT

TGACGAAGCACATATCGTTGGCATGTCACTTGGCGGAATGATTGCGCAAAT

TATTGCGCTTAGACATCCGCAGAGAGTGCTGACAATTACAATGATTGCGTCA

AGCATTTTCGGAAGCGATGATAATAACAGAGACCTTCCGCCGATGGATGAA

AATATTCTTGCGTATCATGCGAACGGAGCGACAGTGAATTGGAGCGATGAA

GAAAGCGTTGCAAATTATCTGGTGGCGGGCTCAGGCCTTCTTTGCGGATCT

AAACATAAATTTGACGAGAAGCGCGTTTACAAACAAGTGAGAAAAGAAAT

CAAGCGCGCGAATAATCTGCTGTCAATGTTTAATCACGCGATTCTGAAAGG

AGATGCAAGCTATGAAGGAAAAATCAAACGCATTAAGGTGCCGGCACTTG

TTATTCATGGAACAGAAGATACAGTCCTGCCGTATGAACATGGACTTGCGCT

GGCGAATGAAATTCCGAATGCGTCACTGCTTACACTGGATGGAACAGGCCA

TGAAATTCATTTTGATGATTGGGATAACATCATCAACGCGATTAGCAATCATA

CATCAGTTGTGTAAGGATCCTCTAGAGTCGACGTCCCCGGGGCAGCCCGCC

TAATGAGCGGGCTTTTTTCACGTCACGCGTCCATGGAGATCTTTGTCTGCAA

CTGAAAAGTTTATACCTTACCTGGAACAAATGGTTGAAACATACGAGGCTA

ATATCGGCTTATTAGGAATAGTCCCTGTACTAATAAAATCAGGTGGATCAGTT

GATCAGTATATTTTGGACGAAGCTCGGAAAGAATTTGGAGATGACTTGCTT

AATTCCACAATTAAATTAAGGGAAAGAATAAAGCGATTTGATGTTCAAGGA

ATCACGGAAGAAGATACTCATGATAAAGAAGCTCTAAAACTATTCAATAAC

CTTACAATGGAATTGATCGAAAGGGTGGAAGGTTAATGGTACGAAAATTAG

GGGATCTACCTAGAAAGCCACAAGGCGATAGGTCAAGCTTAAAGAACCCTT

ACATGGATCTTACAGATTCTGAAAGTAAAGAAACAACAGAGGTTAAACAA

ACAGAACCAAAAAGAAAAAAAGCATTGTTGAAAACAATGAAAGTTGATGT

TTCAATCCATAATAAGATTAAATCGCTGCACGAAATTCTGGCAGCATCCGAA

GGGAATTCATATTACTTAGAGGATACTATTGAGAGAGCTATTGATAAGATGG

TTGAGACATTACCTGAGAGCCAAAAAACTTTTTATGAATATGAATTAAAAA

AAAGAACCAACAAAGGCTGAGACAGACTCCAAACGAGTCTGTTTTTTTAA

AAAAAATATTAGGAGCATTGAATATATATTAGAGAATTAAGAAAGACATGGG

AATAAAAATATTTTAAATCCAGTAAAAATATGATAAGATTATTTCAGAATATG

AAGAACTCTGTTTGTTTTTGATGAAAAAACAAACAAAAAAAATCCACCTA

ACGGAATCTCAATTTAACTAACAGCGGCCAAACTGAGAAGTTAAATTTGAG

AAGGGGAAAAGGCGGATTTATACTTGTATTTAACTATCTCCATTTTAACATTT

TATTAAACCCCATACAAGTGAAAATCCTCTTTTACACTGTTCCTTTAGGTGA

TCGCGGAGGGACATTATGAGTGAAGTAAACCTAAAAGGAAATACAGATGA

ATTAGTGTATTATCGACAGCAAACCACTGGAAATAAAATCGCCAGGAAGAG

AATCAAAAAAGGGAAAGAAGAAGTTTATTATGTTGCTGAAACGGAAGAGA

AGATATGGACAGAAGAGCAAATAAAAAACTTTTCTTTAGACAAATTTGGTA

CGCATATACCTTACATAGAAGGTCATTATACAATCTTAAATAATTACTTCTTT

GATTTTTGGGGCTATTTTTTAGGTGCTGAAGGAATTGCGCTCTATGCTCACC

TAACTCGTTATGCATACGGCAGCAAAGACTTTTGCTTTCCTAGTCTACAAAC

AATCGCTAAAAAAATGGACAAGACTCCTGTTACAGTTAGAGGCTACTTGAA

ACTGCTTGAAAGGTACGGTTTTATTTGGAAGGTAAACGTCCGTAATAAAAC

CAAGGATAACACAGAGGAATCCCCGATTTTTAAGATTAGACGTAAGGTTCC

TTTGCTTTCAGAAGAACTTTTAAATGGAAACCCTAATATTGAAATTCCAGAT

GACGAGGAAGCACATGTAAAGAAGGCTTTAAAAAAGGAAAAAGAGGGTC

TTCCAAAGGTTTTGAAAAAAGAGCACGATGAATTTGTTAAAAAAATGATGG

ATGAGTCAGAAACAATTAATATTCCAGAGGCCTTACAATATGACACAATGTA

TGAAGATATACTCAGTAAAGGAGAAATTCGAAAAGAAATCAAAAAACAAA

TACCTAATCCTACAACATCTTTTGAGAGTATATCAATGACAACTGAAGAGGA

AAAAGTCGACAGTACTTTAAAAAGCGAAATGCAAAATCGTGTCTCTAAGCC

TTCTTTTGATACCTGGTTTAAAAACACTAAGATCAAAATTGAAAATAAAAAT

TGTTTATTACTTGTACCGAGTGAATTTGCATTTGAATGGATTAAGAAAAGAT

ATTTAGAAACAATTAAAACAGTCCTTGAAGAAGCTGGATATGTTTTCGAAA

AAATCGAACTAAGAAAAGTGCAATAAACTGCTGAAGTATTTCAGCAGTTTT

TTTTATTTAGAAATAGTGAAAAAAATATAATCAGGGAGGTATCAATATTTAAT

GAGTACTGATTTAAATTTATTTAGACTGGAATTAATAATTAACACGTAGACTA

ATTAAAATTTAATGAGGGATAAAGAGGATACAAAAATATTAATTTCAATCCC

TATTAAATTTTAACAAGGGGGGGATTAAAATTTAATTAGAGGTTTATCCACA

AGAAAAGACCCTAATAAAATTTTTACTAGGGTTATAACACTGATTAATTTCTT

AATGGGGGAGGGATTAAAATTTAATGACAAAGAAAACAATCTTTTAAGAAA

AGCTTTTAAAAGATAATAATAAAAAGAGCTTTGCGATTAAGCAAAACTCTTT

ACTTTTTCATTGACATTATCAAATTCATCGATTTCAAATTGTTGTTGTATCATA

AAGTTAATTCTGTTTTGCACAACCTTTTCAGGAATATAAAACACATCTGAGG

CTTGTTTTATAAACTCAGGGTCGCTAAAGTCAATGTAACGTAGCATATGATAT

GGTATAGCTTCCACCCAAGTTAGCCTTTCTGCTTCTTCTGAATGTTTTTCATA

TACTTCCATGGGTATCTCTAAATGATTTTCCTCATGTAGCAAGGTATGAGCA

AAAAGTTTATGGAATTGATAGTTCCTCTCTTTTTCTTCAACTTTTTTATCTAA

AACAAACACTTTAACATCTGAGTCAATGTAAGCATAAGATGTTTTTCCAGTC

ATAATTTCAATCCCAAATCTTTTAGACAGAAATTCTGGACGTAAATCTTTTG

GTGAAAGAATTTTTTTATGTAGCAATATATCCGATACAGCACCTTCTAAAAG

CGTTGGTGAATAGGGCATTTTACCTATCTCCTCTCATTTTGTGGAATAAAAAT

AGTCATATTCGTCCATCTACCTATCCTATTATCGAACAGTTGAACTTTTTAAT

CAAGGATCAGTCCTTTTTTTCATTATTCTTAAACTGTGCTCTTAACTTTAACA

ACTCGATTTGTTTTTCCAGATCTCGAGGGTAACTAGCCTCGCCGATCCCGCA

AGAGGCCCGGCAGTCAGGTGGCACTTTTCGGGGAAATGTGCGCGGAACCC

CTATTTGTTTATTTTTCTAAATACATTCAAATATGTATCCGCTCATGAGACAAT

AACCCTGATAAATGCTTCAATAATATTGAAAAAGGAAGAGTATGAGTATTCA

ACATTTCCGTGTCGCCCTTATTCCCTTTTTTGCGGCATTTTGCCTTCCTGTTT

TTGCTCACCCAGAAACGCTGGTGAAAGTAAAAGATGCTGAAGATCAGTTG

GGTGCACGAGTGGGTTACATCGAACTGGATCTCAACAGCGGTAAGATCCTT

GAGAGTTTTCGCCCCGAAGAACGTTTTCCAATGATGAGCACTTTTAAAGTT

CTGCTATGTGGCGCGGTATTATCCCGTATTGACGCCGGGCAAGAGCAACTC

GGTCGCCGCATACACTATTCTCAGAATGACTTGGTTGAGTACTCACCAGTC

ACAGAAAAGCATCTTACGGATGGCATGACAGTAAGAGAATTATGCAGTGCT

GCCATAACCATGAGTGATAACACTGCGGCCAACTTACTTCTGACAACGATC

GGAGGACCGAAGGAGCTAACCGCTTTTTTGCACAACATGGGGGATCATGTA

ACTCGCCTTGATCGTTGGGAACCGGAGCTGAATGAAGCCATACCAAACGA

CGAGCGTGACACCACGATGCCTGTAGCAATGGCAACAACGTTGCGCAAAC

TATTAACTGGCGAACTACTTACTCTAGCTTCCCGGCAACAATTAATAGACTG

GATGGAGGCGGATAAAGTTGCAGGACCACTTCTGCGCTCGGCCCTTCCGGC

TGGCTGGTTTATTGCTGATAAATCTGGAGCCGGTGAGCGTGGGTCTCGCGG

TATCATTGCAGCACTGGGGCCAGATGGTAAGCCCTCCCGTATCGTAGTTATC

TACACGACGGGGAGTCAGGCAACTATGGATGAACGAAATAGACAGATCGC

TGAGATAGGTGCCTCACTGATTAAGCATTGGTAACTGTCAGACCAAGTTTA

CTCATATATACTTTAGATTGATTTAAAACTTCATTTTTAATTTAAAAGGATCTA

GGTGAAGATCCTTTTTGATAATCTCATGACCAAAATCCCTTAACGTGAGTTT

TCGTTCCACTGAGCGTCAGACCCCGTAGAAAAGATCAAAGGATCTTCTTGA

GATCCTTTTTTTCTGCGCGTAATCTGCTGCTTGCAAACAAAAAAACCACCG

CTACCAGCGGTGGTTTGTTTGCCGGATCAAGAGCTACCAACTCTTTTTCCG

AAGGTAACTGGCTTCAGCAGAGCGCAGATACCAAATACTGTCCTTCTAGTG

TAGCCGTAGTTAGGCCACCACTTCAAGAACTCTGTAGCACCGCCTACATAC

CTCGCTCTGCTAATCCTGTTACCAGTGGCTGCTGCCAGTGGCGATAAGTCGT

GTCTTACCGGGTTGGACTCAAGACGATAGTTACCGGATAAGGCGCAGCGGT

CGGGCTGAACGGGGGGTTCGTGCACACAGCCCAGCTTGGAGCGAACGAC

CTACACCGAACTGAGATACCTACAGCGTGAGCTATGAGAAAGCGCCACGCT

TCCCGAAGGGAGAAAGGCGGACAGGTATCCGGTAAGCGGCAGGGTCGGA

ACAGGAGAGCGCACGAGGGAGCTTCCAGGGGGAAACGCCTGGTATCTTTA

TAGTCCTGTCGGGTTTCGCCACCTCTGACTTGAGCGTCGATTTTTGTGATGC

TCGTCAGGGGGGCGGAGCCTATGGAAAAACGCCAGCAACGCGGCCTTTTT

ACGGTTCCTGGCCTTTTGCTGGCCTTTTGCTCACATGTTCTTTCCTGCGTTAT

CCCCTGATTCTGTGGATAACCGTATTACCGCCTTTGAGTGAGCTGATACCGC

TCGCCGCAGCCGAACGACCGAGCGCAGCGAGTCAGTGAGCGAGGAAGCG

GAAGAGCGCCCAATACGCATGC
